# European Stroke Organisation and European Academy of Neurology joint guidelines on post-stroke cognitive impairment

**DOI:** 10.1177/23969873211042192

**Published:** 2021-10-08

**Authors:** Terence J Quinn, Edo Richard, Yvonne Teuschl, Thomas Gattringer, Melanie Hafdi, John T O’Brien, Niamh Merriman, Celine Gillebert, Hanne Huyglier, Ana Verdelho, Reinhold Schmidt, Emma Ghaziani, Hysse Forchammer, Sarah T Pendlebury, Rose Bruffaerts, Milija Mijajlovic, Bogna A Drozdowska, Emily Ball, Hugh S Markus

**Affiliations:** 1 Institute of Cardiovascular and Medical Sciences, 3526 University of Glasgow, Glasgow, UK; 2 Department of Neurology, Donders Institute for Brain, Behaviour and Cognition, 2152 Radboud University Medical Centre, Nijmegen, The Netherlands; 3 Department for Clinical Neurosciences and Preventive Medicine, 31227 Danube University Krems, der Donau, Austria; 4 Department of Neurology and Division of Neuroradiology, Vascular and Interventional Radiology, Department of Radiology, 31475 Medical University of Graz, Graz, Austria; 5 Academic Medical Center, 1234 University of Amsterdam, Amsterdam, The Netherlands; 6 Department of Psychiatry, 1234 University of Cambridge School of Clinical Medicine, Cambridge, UK; 7 Deptartment of Health Psychology, Division of Population Health Sciences, 162838 Royal College of Surgeons in Ireland, Dublin, Ireland; 8 Department Brain & Cognition, Leuven Brain Institute, KU Leuven, Leuven, Belgium; 9 TRACE, Centre for Translational Psychological Research (TRACE), KU Leuven – Hospital East-Limbourgh, Genk, Belgium; 10 Department of Neurosciences and Mental Health, Hospital de Santa Maria, Lisbon, Portugal; 11 Department of Neurology, 31475 Medical University of Graz, Graz, Austria; 12 Department of Physical and Occupational Therapy, Bispebjerg and Frederiksberg Hospital, Copenhagen, Denmark; 13 The Danish Stroke Association, 553791 Hjernesagen, Denmark, Denmark; 14 Departments of Medicine and Geratology and NIHR Oxford Biomedical Research Centre, John Radcliffe Hospital, 6397 Oxford University Hospitals NHS Foundation Trust, Oxford, UK; 15 Biomedical Research Institute, 54496 Hasselt University, Hasselt, Belgium; 16 Neurosonology Unit, Neurology Clinic, 119081 University Clinical Center of Serbia and Faculty of Medicine University of Belgrade, Belgrade, Serbia; 17 Centre for Clinical Brain Sciences, 215746 University of Edinburgh, Edinburgh, Scotland; 18 Stroke Research Group, Department of Clinical Neurosciences, 12204 University of Cambridge, Cambridge, UK

**Keywords:** Cognition, dementia, diagnosis, guidelines, stroke, prognosis

## Abstract

The optimal management of post-stroke cognitive impairment remains controversial. These joint European Stroke Organisation (ESO) and European Academy of Neurology (EAN) guidelines provide evidence-based recommendations to assist clinicians in decision making around prevention, diagnosis, treatment and prognosis. These guidelines were developed according to ESO standard operating procedure and the Grading of Recommendations, Assessment, Development and Evaluation (GRADE) methodology. The working group identified relevant clinical questions, performed systematic reviews and, where possible, meta-analyses of the literature, assessed the quality of the available evidence and made specific recommendations. Expert consensus statements were provided where insufficient evidence was available to provide recommendations based on the GRADE approach. There was limited randomised controlled trial evidence regarding single or multicomponent interventions to prevent post-stroke cognitive decline. Interventions to improve lifestyle and treat vascular risk factors may have many health benefits but a beneficial effect on cognition is not proven. We found no evidence around routine cognitive screening following stroke but recognise the importance of targeted cognitive assessment. We described the accuracy of various cognitive screening tests but found no clearly superior approach to testing. There was insufficient evidence to make a recommendation for use of cholinesterase inhibitors, memantine nootropics or cognitive rehabilitation. There was limited evidence on the use of prediction tools for post-stroke cognitive syndromes (cognitive impairment, dementia and delirium). The association between post-stroke cognitive impairment and most acute structural brain imaging features was unclear, although the presence of substantial white matter hyperintensities of presumed vascular origin on acute MRI brain may help predict cognitive outcomes. These guidelines have highlighted fundamental areas where robust evidence is lacking. Further, definitive randomised controlled trials are needed, and we suggest priority areas for future research.

## Introduction

Cognitive impairment is a common and potentially disabling effect of stroke.^1^ Post-stroke cognitive impairment is a collective term for differing pathological processes, but regardless of the underlying aetiology, stroke survivors and their caregivers consistently rate problems of memory and thinking as their greatest concern.^2^ Despite the importance of post-stroke cognitive problems, this is an area of stroke care where there are substantial rates of underdiagnosis in clinical practice and a disproportionate lack of research activity. As a result, there is substantial variation in management of post-stroke cognitive issues across Europe. It is noticeable that post-stroke cognitive impairment is mentioned in only a small number of the many national and international guidelines available for stroke care. The apparent disconnect between clinical relevance and available evidence is thankfully changing, large cohorts and other studies are underway which should help us better understand and manage post-stroke cognitive impairment.^3^ In the meantime, clinicians may benefit from a synthesis of the available research that allows evidence-based, or expert informed, guidance on post-stroke cognitive impairment.

In this context, the European Stroke Organisation (ESO) commissioned a guideline, in agreement with the Stroke Scientific Panel of the European Academy of Neurology (EAN), with a focus on post-stroke cognitive impairment. The intention with this guideline was to provide a useful resource for health professionals and researchers from multiple disciplines, as well as policy makers. Recognising that the potential scope of this guideline was broad, we chose to focus on four specific areas of clinical importance: prevention, diagnosis, management and prognosis.

The guideline followed best practice and adhered to the standard operating procedure of the ESO Guideline Group.^4^ The methods that informed the formulation of our recommendations and consensus statements are described later in the text. However, there are certain aspects of our approach that are worthy of mention early in the guideline and will be discussed here.

In planning the work, we were keen that we represent all the clinical disciplines involved in managing people living with stroke and subsequent post-stroke cognitive issues. Thus, we stipulated that our core guideline writing group would comprise expertise in geriatric medicine, psychology, psychiatry, neuropsychology, neurology and occupational therapy in addition to a representative of a stroke society.

Arguably a barrier to progress in the broad field of vascular cognitive impairment is the lack of consensus definitions for the syndromes of interest.^5^ In this guideline, we took an inclusive approach, defining the concept of post-stroke cognitive impairment, as all problems in cognitive function that occur following a stroke, irrespective of the aetiology. We make a deliberate distinction between the broad construct of cognitive impairment and the more defined concept of dementia (or major neurocognitive disorder), and we consider the two constructs separately in the guideline. For many of our questions, we consider the concept of cognitive decline, that is, change in cognitive function over time.

It would be almost impossible to cover every important clinical question that is relevant to the field of post-stroke cognitive impairment.^6^ We did not restrict our remit to those areas where we knew we would find high-quality trials. Rather, we turned our attention to those aspects of stroke care where we felt the need for clinical guidance was most pressing. To achieve this, we used relatively novel approaches to evidence synthesis. We were aware that for some topics definitive answers could not be achieved with this methodology. We planned that where an evidence-based recommendation was not possible, we would provide an expert opinion taking in consideration all the available information and drawing on the experience and knowledge of our multidisciplinary writing group.

The stroke dementia research space has been criticised for having too many small studies with inherent methodological limitations.^6^ To ensure our recommendations did not suffer from the same biases, for many of our PICO questions, we pre-specified strict inclusion criteria around study method (randomised controlled trials–RCTs), population size, duration of follow-up and study design. Applying these criteria necessarily means that certain well-known articles would not be included in the evidence that informed our recommendations. We felt that post-stroke cognitive impairment was too important to allow the inclusion of potentially misleading studies. Anticipating that some areas may have few included studies, as a final part of the guideline writing process, we used the available evidence to select key research questions that should be a priority for future studies.

## Methods

### Composition of the writing group

These guidelines were jointly initiated by the ESO and EAN. A Module Working Group (MWG) was established, consisting of 15 experts (TQ, HSM and co-chairs). The MWG was joined by four fellows (MH, HH, BAD and EB) who assisted with abstract and full text screening, data extraction and drafting the text. Fellows were all either trainee neurologists or post-doctoral fellows interested in stroke or neuro-epidemiology. The composition of the MWG was designed to include those disciplines involved in the care of people living with post-stroke cognitive issues and comprised multidisciplinary expertise. Attention was given to achieving diversity in terms of sex and geography. The group included the Chief Executive Officer of the Danish Stroke Association to facilitate stroke survivor views. The composition of this group was approved by the ESO Guidelines Board and the ESO Executive Committee, based on a review of the intellectual and financial disclosures of the proposed members.

### Selection of population, intervention, comparator and outcome

The guidelines were developed using the Grading of Recommendations, Assessment, Development and Evaluation (GRADE) methodology^7^ and the ESO Standard Operating Procedure.^4^

The MWG developed a list of topics and corresponding outcomes of clinical interest. The outcomes were rated as critical, important or of limited importance according to GRADE criteria. The MWG voted in a closed ballot to identify which questions were highest priority.

After initial scoping meetings, four subgroups were formed to develop recommendations in thematic areas of prevention, diagnosis, treatment and prognosis. Each subgroup had a chair and at least two other members (see contribution section for details of each subgroup).

These subgroups formulated three to five main PICO (Population, Intervention, Comparator and Outcome) questions. The outcomes chosen for each PICO favoured those rated as ‘critical’ by the MWG. These were subsequently approved by the ESO Guidelines Board and the ESO Executive Committee.

For each PICO question, search terms were identified, tested, refined and agreed by each writing subgroup. Search terms were developed in partnership with the Cochrane Dementia Group. Where a validated search strategy was available, this was used or adapted. Where there was a recent relevant systematic review on the question of interest, the corresponding search strategy and results were used and updated as necessary. Each search strategy is described in the Supplementary Materials.

### Identification and selection relevant studies

At least two members of each writing subgroup independently screened the titles and abstracts of publications and assessed the full text of potentially relevant studies. We focused on randomised controlled trials but considered other types of study such as health registry data analyses and large observational studies since we anticipated a lack of high-quality RCTs. We noted potentially relevant ongoing studies for future reference. All disagreements were resolved by discussion between the two authors or by a third MWG author. We searched reference lists of review articles, the authors own reference libraries and previous guidelines for additional relevant material.

Recognising the potential limitations in the post-stroke cognition field, we made a series of a-priori decisions around inclusion, considering study methodology, sample size and duration of follow-up. These are detailed in the corresponding PICO sections.

For each question, the writing subgroup, assisted by one or more fellows, evaluated the available evidence. The risk of selection, performance, detection, attrition and reporting biases in each randomised trial was assessed. For randomised controlled trials, the assessment used the standard Cochrane tool.^8^ This guideline was not restricted to interventional RCTs and we adapted our assessment of risk of bias and quality of evidence to suit the component data.^9^ Where the assessment did not use the standard approach outlined in the ESO guideline Standard Operating Procedure, any modification, and the relevant tools employed, are described in the relevant PICO section. In the evidence synthesis, we did not use an overall quality ‘score’ as such an approach is now discouraged.^9^ The classification of low or high risk of bias was performed by the assessors at individual study level.

For each PICO question, the quality of evidence was rated using the GRADEpro Guideline Development Tool (McMaster University, 2015; developed by Evidence Prime, Inc.) using guidelines for non-pooled data as necessary.^7^ Final quality ratings were categorised as high, moderate, low or very low. GRADE assessment was performed within writing subgroups and then shared with the complete MWG for discussion and consensus. Text was discussed in open forum through monthly team calls, members of the complete MWG then voted on the text using a Delphi approach. Complete consensus was required for the Recommendation statements, and text was revised until consensus was reached. For Expert Consensus Statements, complete consensus was not mandated, but where there was disagreement in the group this was described as part of the Statement.

The writing subgroups analysed the available primary and any additional data, prepared tables and figures and drafted three sections of text: ‘analysis of current evidence’ which focused on relevant primary studies and/or systematic reviews, ‘additional information’ to summarise indirect evidence and provide context and ‘expert consensus statement’ which allowed for practical guidance where the available evidence was not sufficient to support a recommendation. Here the processes of ESO and EAN have certain differences. The EAN collates indirect evidence under a heading ‘good clinical practice statements’, whereas ESO collates additional relevant information and expertise under a heading of ‘Expert Consensus Statement’. We followed the ESO process and terminology in formulating our text.

The Expert Consensus Statements are based on voting by all expert MWG members. Importantly, these Expert Consensus Statements should not be regarded as evidence-based recommendations, since they only reflect the opinion of the MWG. Where there was not complete consensus across all members of the MWG, this is described as part of the Consensus Statement.

The Guidelines document was reviewed several times by all MWG members. Modifications to the wording of Recommendations and Expert Consensus used a Delphi approach. We required consensus for the Recommendations text. The final draft was reviewed by the Chairs of the ESO Guideline Committee and the EAN Guideline Production Group. The document was subsequently reviewed and approved by two external reviewers, members of the ESO executive committee, and the Editor and peer reviewers of the *European Stroke Journal*.

## Results

### Prevention

#### **PICO 1:** In people with a history of stroke, do monitored lifestyle-based interventions (exercise, dietary change, alcohol moderation, weight loss and smoking cessation), alone or in combination, compared to care as usual, prevent: future cognitive decline or dementia?

##### Analysis of current evidence

The intervention of interest was non-pharmacological lifestyle interventions that are prescribed and monitored. We pre-specified that we would only include randomised controlled trials (RCTs) as observational data in the field are prone to many biases. We also pre-specified that trials would require a minimum of 6 months follow-up and 50 participants per arm because we felt as a writing group that smaller, short-term follow-up, studies should be considered proof of concept and are more prone to publication bias.

The literature search identified five relevant RCTs comparing monitored lifestyle-based interventions with care as usual for the prevention of future cognitive decline and dementia.

*Multidomain interventions.* Three studies examined the effects of an intervention on multiple lifestyle domain simultaneously (Austrian Polyintervention Study to prevent cognitive decline after ischaemic stroke [*ASPIS*];^10^ blood pressure, lipid and glycaemic control, healthy diet, physical activity and cognitive training, *Ihle-Hansen* et al.*;*^11^ advice on risk factor management, smoking cessation courses, physical activity and healthy diet, *Cheng* et al.;^12^ cognitive and rehabilitation training). These trials recruited, respectively, 202, 195 and 168 patients (*n* = 565 in total), with a history of stroke. All participants were directly recruited after their initial diagnosis of stroke; two studies (Ihle-Hansen et al. and Cheng et al.) only included patients with a first ever stroke. The risk of bias in each trial was considered low (Supplementary Materials). There was no blinding of patients or staff due to the nature of the interventions, but outcome assessment was blinded. One study (Ihle-Hansen et al.) reported dementia incidence and found no effect of the intervention after 12 months (OR: 0.65 (95% CI: 0.24–1.48); the ASPIS study had no cases of incident dementia. Assessment instruments for cognitive decline varied widely between studies. No study reported significant change in cognitive outcomes between the intervention and control groups.

*Physical activity interventions*. Two studies investigated the effect of physical activity on cognitive decline. In total, these trials recruited 500 patients with a history of stroke, 240 patients received an exercise programme delivered by physiotherapists, and 254 participants received care as usual. Intervention periods ranged from 12 to 18 months and follow-up from 18 to 24 months. The Life After Stroke Trial (LAST)^13^ recruited patients 3 months post-stroke, the MoveIT trial^14^ within 1 month. Overall, the risk of bias in these trials was low (Supplementary Materials). There was no blinding of patients or staff due to the nature of the interventions, but outcome assessment was blinded. The LAST study found no effect of a physical activity intervention on Mini Mental State Examination (MMSE) score or Trail Making Test B (TMT-B) (between group differences: −0.1 (95% CI: −0.8 to 0.6) and 8.6 (95% CI: −16.5 to 33.6), respectively. There was a significant difference in Trail Making Test A scores (TMT-A) in favour of the intervention group (between group difference 8.6 (95% CI: −16.5 to 33.6)). The MoveIT trial did not find an effect on global cognitive functioning after 2 years (between group difference in Montreal Cognitive Assessment (MoCA) score −0.3, *p* = 0.66).

Findings are summarised in Table 1. In making our recommendations we considered the strength of evidence for preventing cognitive decline and dementia and limited our recommendation to those outcomes only. We recognise that lifestyle interventions have many other physical and mental health benefits and would not dissuade clinicians from trying to improve lifestyle factors for other non-cognitive reasons. We downgraded the evidence to very low-quality evidence for imprecision, as confidence intervals included both potentially beneficial and harmful effects and imprecision, as the cognitive outcome measures used were very heterogeneous and not all validated to assess cognitive decline over time.

**Table 1.** Summary of findings for PICO 1. Monitored lifestyle-based interventions (exercise, dietary change, alcohol moderation, weight loss and smoking cessation), alone or in combination, compared to care as usual, for prevention of future cognitive decline or dementia.

Certainty assessment							Number of patients		Effect		Quality of evidence	Importance
Number of studies	Study design	Risk of bias	Inconsistency	Indirectness	Imprecision	Other	Lifestyle interventions	Usual care	Relative (95% CI)	Absolute (95% CI)		
**Dementia**												
1	Randomised trials	Not serious	Not serious	Not serious	Very serious	None	11/85 (12.9%)	17/91 (18.7%)	**OR 0.65** (0.28 to 1.48)	**57 fewer per 1.000** (from 126 fewer to 67 more)	⊕○○○ Ver low	Important
**Cognitive decline (assessed with: various tools)**												
5	Randomised trials	Not serious	Serious	Serious	Serious	None	The heterogeneity in interventions and outcomes precluded quantitative meta-analysis. None of the included studies found a significant effect of their lifestyle intervention on cognitive decline.				⊕○○○ Very low	Important

CI: confidence interval; OR: odds ratio.

##### Additional information

Our literature search found unpublished RCTs that could be relevant to the PICO question. We reached out to the authors of three unpublished trials that could reasonably be finished at the time of data extraction but did not get a response (Vitality (NCT01916486), AFIVASC (NCT03578614) and Bai). For the MoveIT study, we could only obtain part of the results in a conference abstract; we have contacted the study authors but did not receive a response. We found reviews of exercise interventions for preventing cognitive decline that included stroke survivors,^15,16^ but the included studies did not meet our inclusion criteria. The reviews concluded a possible beneficial cognitive effect of increasing physical activity but recognised methodological limitations in the studies.

##### Vitamin suppletion

Two studies (VITATOPS and VISP)^17,18^ were not included as we did not regard vitamin suppletion as a monitored lifestyle intervention. Both studies investigated the effect of B-vitamin suppletion on cognitive decline and did not find an effect of this daily suppletion on cognitive decline as measured by the MMSE.

Although we found no consistent evidence that lifestyle interventions are beneficial for the prevention of post-stroke cognitive decline or dementia, there are other reasons why lifestyle changes after stroke may still be warranted, such as secondary stroke prevention, future cardiovascular disease prevention and better physical health in general.^19^

#### **PICO 2:** In people with a history of stroke, does **monitored intensive management of vascular risk factors**, compared to usual care, prevent: future cognitive decline or dementia?

##### Analysis of current evidence

The intervention of interest was ‘intensive’ management of traditional cardiovascular risk factors. Intensive management was defined as treatment of cardiovascular risk factors beyond what would be expected as standard practice at the time of the study. The two likely models of intervention we anticipated were intervention(s) to reach treatment targets that are more aggressive than described in contemporary guidelines and/or intervention(s) to reach guideline targets in populations where these targets are not reached. As with other PICOs in this section, we pre-specified that we would only include randomised controlled trials (RCTs) and required a minimum of 50 participants per arm.

The literature search identified five RCTs, comparing the management of three different vascular risk factors. In our Summary of Findings table (Table 2), we assess the evidence for intensive treatment in aggregate. In the text below, we also consider three pharmacological interventions individually.

**Table 2.** Summary of findings for PICO 2. Monitored intensive management of vascular risk factors compared to usual care for the prevention of post-stroke cognitive decline or dementia.

Certainly assessment	Number of patients	Effect					
Number of studies	Study design	Intensive management	Usual care	Relative (95% CI)	Absolute (95% CI)		
**Dementia**							
3	Randomised trials	633/12455 (5.1%)	659/12485 (5.3%)	**OR 0.96** (0.86 to 1.07)	**2 fewer per 1.000** (from 7 fewer to 3 more)	⊕○○○ Very low	Important
**Cognitive decline (assessed with: various tools)**							
5	Randomised trials	The heterogeneity in interventions and outcomes precluded quantitative meta-analysis.				⊕○○○ Very low	Important

CI: confidence interval; OR: odds ratio.

Abbreviated for space, full PICO in Supplementary Material.

*Hypertension.* Four RCTs investigated the effect of intensive vascular management of hypertension on dementia and cognitive decline; three studies compared antihypertensive treatment: nimodipine in preventing cognitive impairment in ischaemic cerebrovascular event^20^ (NICE, 30 mg three times daily), Prevention Regimen for Effectively Avoiding Second Strokes^21^ (PRoFESS, telmisartan 80 mg daily) and Perindopril Protection Against Recurrent Stroke Study^22^ (PROGRESS, perindopril 4 mg daily ± indapamide 2.5 mg daily)) with placebo. One study compared two different blood pressure targets (Secondary Prevention of SubCortical Stroke Study^23^ (SPS3; <130 mmHg vs 130–149 mmHg, open-label)) in patients with recent lacunar stroke. These trials recruited, respectively, 654, 3020, 20.332 and 6105 patients (30,111 in total; 15,018 intervention and 15,093 control group), with a history of stroke. Three studies only included participants with a recent ischaemic stroke (NICE <7 days, SPS3, <6 months and PRoFESS, <90 days), one study included participants with a history of stroke (ischaemic and haemorrhagic, no subarachnoid haemorrhage) in the previous 5 years (PROGRESS). The risk of bias in each trial was considered low (Supplementary Materials).

**Figure 1.**
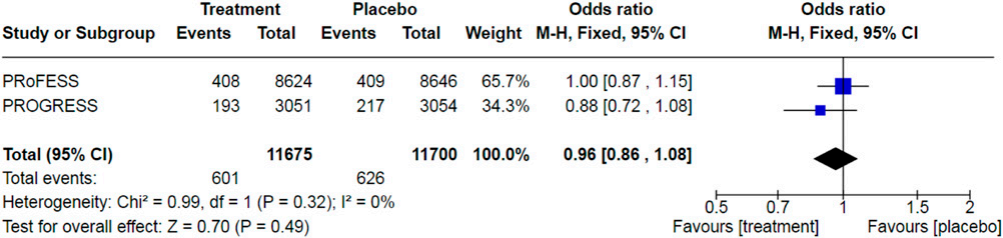
Pooled odds ratio for dementia incidence in post-stroke patients treated antihypertensive medication. Fixed-effects meta-analysis.

**Figure 2.**
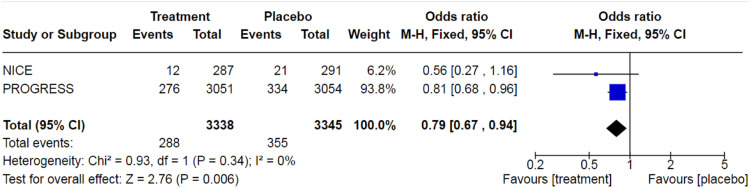
Pooled odds ratio for cognitive decline (drop in MMSE ≥3 points since baseline) in post-stroke patients treated antihypertensive medication. Fixed-effects meta-analysis.

There was no effect of antihypertensive treatment versus placebo on dementia incidence (pooled OR: 0.96 (95% CI: 0.86–1.08); two studies (PRoFESS and PROGRESS); 23,375 participants; Figure 1) nor was there an effect of blood pressure reduction on incident mild cognitive impairment (MCI) (OR: 0.94 (95% CI: 0.80–1.10); one study). Operationalisation of cognitive decline was heterogeneous. Three studies did not find an effect of intensive blood pressure management on cognitive decline (NICE, ADAS-Cog ≥4 point decrease since baseline OR: 0.93(95% CI: 0.52–1.66); SPS3, between group mean difference (MD) 0.12 Cognitive Assessment Screening Instrument (CASI), *p* = 0.520; PRoFESS, MMSE <25 OR: 0.95 (95% CI: 0.86–1.05)). For two studies only (NICE, PROGRESS; 6683 participants), there was a modest effect of antihypertensive treatment on prevention of cognitive decline, when operationalised as ≥3 points drop in MMSE score at end of study follow-up (pooled OR: 0.79 (95% CI: 0.67–0.94); Figure 2). While this result is encouraging, it is not completely aligned with our specified outcomes and the lack of treatment effect for dementia and MCI leads to serious concerns over inconsistency.

*Antithrombotic therapy.* One RCT investigated the effect of short-term dual antiplatelet treatment on cognitive function in patients with a recent (<6 months) lacunar infarction (SPS3,^24^ aspirin 325 mg plus clopidogrel 75 mg vs aspirin 325 mg plus placebo), including 3020 participants in total. The risk of bias in this study was considered low (Supplementary Materials). This study did not find an effect of dual antiplatelet therapy on MCI incidence (OR: 0.94 (95% CI: 0.81–1.10)) or cognitive decline (between group mean difference (MD) 0.14 CASI points, *p* = 0.858). However, risk of bleeding was increased.

*Statin treatment.* One RCT investigated the effect of 10 mg pravastatin versus placebo on dementia incidence and cognitive impairment assessed by the clinical dementia rating (CDR) and MMSE in 1578 participants.^25^ As statin therapy is now considered standard following ischaemic stroke, it is debatable whether this intervention represents intensive risk factor modification. The risk of bias in this study was considered low (Supplementary Materials). In this study, there was no effect of the intervention on dementia incidence (risk difference 0.10%, *p* = 0.94) or cognitive decline (CDR between group mean difference (MD) −0.1, *p* = 0.53; MMSE between group MD: 0.2 (*p* = 0.18)).

##### Additional information

Consensus on the management of vascular risk factors in secondary prevention has been adapted many times over the past decades and is still continuously evolving. Treatments considered ‘intensive’ at one time are now considered routine practice. Although not included in our synthesis due to the numbers included being less than our pre-specified threshold, the Prevention of Decline in Cognition after Stroke Trial’ (PODCAST)^26^ and Screening and Enhanced Risk factor management to prevent Vascular Event related Decline in Memory (SERVED-Memory)^27^ RCTs serve as good examples of the ‘moving target’ of stroke secondary prevention. In both trials recruitment and retention was challenging, partly because the intensive treatment arm was considered best practice by some clinicians. This potential lack of equipoise needs to be considered if designing future trials in this area.

Although we found no consistent evidence that intensive treatment of vascular risk factors is beneficial for the prevention of post-stroke cognitive decline or dementia, management of these risk factors is still warranted in stroke patients for the prevention of secondary stroke or concurring cardiovascular disease.

#### **PICO 3:** In people with a history of stroke, **do monitored multicomponent interventions (lifestyle and pharmacological)**, compared to usual care, prevent: future cognitive decline or dementia?

##### Analysis of current evidence

The intervention of interest was multicomponent interventions, defined as intervention that include more than one potentially active treatment and that are not limited to drug therapy alone. As with other PICOs in this section, we pre-specified that we would only include randomised controlled trials (RCTs) as observational data in the field are prone to many biases. We also pre-specified that trials would require a minimum of 50 participants per arm, because we felt as a writing group that smaller trials are unlikely to show an effect. At the time of setting the PICO questions, we anticipated that multicomponent intervention RCTs would be distinct from the lifestyle or vascular risk factor intervention studies reviewed in previous sections. However, there was considerable overlap.

The literature search identified one relevant RCT comparing a monitored multicomponent intervention with care as usual for the prevention of cognitive decline after stroke. This study also met criteria for PICO 1 and is fully assessed in that section. We did not identify any literature on the prevention of dementia.

The ASPIS study^10^ included 202 participants (101 intervention and 101 control group) aged 40 to 80 years with a clinical diagnosis of ischaemic stroke within the previous 3 months. The intervention consisted of intensive management and motivation for compliance with clinical therapy, adequate blood pressure, lipid and glycaemic control, healthy diet, regular physical activity and cognitive training. This study found no benefit of 24-month multidomain intervention on the incidence of post-stroke cognitive decline in comparison with standard stroke care (RR (95% CI) 0.87 (0.36–2.10)). There were no data on the clinical outcome of incident dementia and so we felt there were issues with indirectness and this is reflected in the GRADE assessment.

Findings are summarised in Table 3. We downgraded the evidence on prevention of cognitive decline to low-quality evidence for imprecision, as the effect came from one single study and the confidence intervals included both beneficial as harmful effects.

**Table 3.** Summary of findings for PICO 3. Monitored multicomponent interventions (lifestyle and pharmacological), compared to usual care for prevention of future cognitive decline or dementia?

Certainty assessment							Number of patients		Effect		Quality of evidence	Importance
Number of studies	Study design	Risk of bias	Inconsistency	Indirectness	Imprecision	Other considerations	Monitored multicomponent interventions	Usual care	Relative (95% CI)	Absolute (95% CI)		
**Cognitive decline (statistically significant decrease of function in at least 2 of 5 cognitive domains)**												
1	Randomised trials	Not serious	Not serious	Serious	Very serious	None	8/76 (10.5%)	10/83 (12.0%)	**OR 0.86** (0.32 to 2.30)	**15 fewer per 1.000** (from 78 fewer to 119 more)	⊕○○○ Very low	Important

CI: confidence interval; OR: odds ratio. PICO: Population, Intervention, Comparator and Outcome.

##### Additional Information

We found limited evidence on the effectiveness of multicomponent interventions for the prevention of cognitive decline and dementia in post-stroke patients. The evidence is in line with several large multicomponent intervention studies in the general population that did not find an effect on dementia incidence or cognitive decline.^28,29^ However, there are other reasons why risk factor modification (both lifestyle and pharmacological) is still warranted after stroke, such as secondary stroke and cardiovascular disease prevention.

#### **PICO 4:** In people with a history of stroke**, does cognitive training,** compared to usual care prevent: future cognitive decline or future dementia?

##### Analysis of current evidence

The intervention of interest was cognitive training, which could include both electronic/computerised training and more traditional pen and paper-based training platforms. We used the definition of cognitive training developed for Cochrane reviews in the field: ‘Cognitive training involves guided practice on a set of standardised tasks designed to reflect particular cognitive functions, such as memory, attention, or problem solving’.^30^ As with other PICOs in this section, we pre-specified that we would only include randomised controlled trials (RCTs) and required a minimum of 50 participants per arm. Finally, we pre-specified that duration of follow-up should be at least 6 months to demonstrate convincing sustained cognitive benefit.

The literature search did not identify any suitable RCT directly addressing this PICO question, that is, we found no RCT investigating cognitive training as the sole intervention and including more than 50 participants per group over a period longer than 6 months.

##### Additional information

A number of trials of cognitive training with sample sizes and intervention periods less than our pre-specified thresholds are available and are summarised in various reviews.^31,32^ In general, trials of cognitive training in stroke have reported low-quality evidence for small beneficial effects. Trials generally investigated the effects of cognitive training for remediation of cognitive impairments, rather than our outcomes of interest of cognitive decline or dementia. In general, outcomes were assessed shortly after intervention and benefits demonstrated may be smaller than a minimal clinically important difference. Trials mainly targeted single cognitive domain deficits such as aphasia and neglect and are less relevant to our PICO question of global prevention of cognitive decline. We refer to the section of this guideline on treatment for a discussion of the evidence on cognitive rehabilitation for prevalent cognitive impairments.

Several recent reviews have investigated the effect of cognitive training in healthy older adults or in people with mild cognitive impairment and have been summarised in an overview by Gavelin.^33^ Meta-analysis reported effect sizes ranging from Hedges’ g = 0.13 to 0.64 in healthy adults (19 reviews) and from g = 0.32 to 0.60 in people with mild cognitive impairment (5 reviews), favouring cognitive training compared to active or passive control groups. The quality of evidence ranged from critically low-to-medium. Sample sizes of most studies were small-to-medium, and only few trials had follow-up periods longer than 6 months or reported dementia incidence. It is unclear if these benefits translate into a sustained effect of prevention of dementia. It is also debatable whether evidence from healthy older adults can inform post-stroke care. People living with stroke, especially those with stroke related impairments, may need more adaptations of cognitive training interventions.

Observational studies suggest that education, cognitively stimulating activity and social interactions can protect against cognitive decline and dementia.^34-36^ These associations have also been observed in stroke cohorts.^37,38^ However, we must be wary of making causal inferences. Although not within the scope of our PICO, an RCT of 103 patients admitted to a neurorehabilitation ward (51% stroke) reported that patients offered enriched activities had larger improvements in cognitive scores at discharge and 3 months than a control group offered usual ward-based activities.^39^

#### **PICO 5:** In people with a history of post-stroke dementia does**, stopping pharmacological management of vascular risk factors (de-prescribing),** compared to continuing these medications prevent: future cognitive decline or improve health related quality of life?

##### Analysis of current evidence

For this PICO the population of interest and focus are different to the other PICO questions in this section. Here, we are concerned with people living with a post-stroke cognitive syndrome and the intervention is stopping existing medication rather that starting a new medication. We separately considered blood pressure management and statins. As with other PICOs in this section, we pre-specified that we would only include randomised controlled trials (RCTs) and required a minimum of 50 participants per arm.

Pharmacological treatment of vascular risk factors is an important strategy to prevent recurrent stroke and cardiovascular disease following stroke. As vascular risk factors and associated (cerebro-) vascular disease are related to cognitive impairment/dementia, control of hypertension and dyslipidaemia is generally recommended for dementia prevention. A recent European Academy of Neurology guideline on medical management of dementia suggested this advice should also apply to people living with mild–moderate dementia.^40^ For people with severe dementia and anticipated short life expectancy the risk-benefit of managing vascular risk is less clear. Pharmacological treatment of vascular risk factors is associated with adverse effects and could potentially have a detrimental impact on cognition. For example, antihypertensive drugs hypothetically increase the risk of cerebral hypoperfusion that could worsen cognition.

Our literature search did not identify any RCT on the cognitive effect of withdrawal of antihypertensive medication in people with post-stroke dementia. There were RCTs describing antihypertensive withdrawal in people living with dementia and stroke and these are considered in the Additional Information section below. The literature search did not identify any RCT on the cognitive effect of statin withdrawal in people with post-stroke dementia or undifferentiated dementia.

##### Additional information

**Antihypertensive withdrawal:** We found two RCTs describing antihypertensive drug withdrawal and cognitive effects, and these did not fulfil our selection criteria. One trial only investigated stopping of pre-existing antihypertensives in the acute phase (first 7 days) of stroke.^41^ The other trial recruited older adults with mild cognitive impairment but free of stroke.^42^ Both studies assessed only short-term cognitive outcomes (three months and 16 weeks, respectively). A Cochrane meta-analysis on antihypertensive de-prescribing concluded that there is insufficient evidence regarding the effect of antihypertensive drug withdrawal on cognitive function and prevention of dementia.^43^

A prospective observational study evaluated whether discontinuation of antihypertensive medication was associated with memory complaints or incident dementia in community-dwelling older people (70–78 years) during 6–8 years of follow-up.^44^ Of 1451 participants with available follow-up information, 85 stopped antihypertensive medication. Dementia occurred more often in the discontinuation group (13.4% vs 6.2%, *p* = 0.02), while mortality was similar (16.5% vs 13.9%, *p* = 0.52). Antihypertensive discontinuation was not associated with change in subjective memory complaints. Notably, around roughly 15% of included participants had a history of stroke. The theoretical concern over antihypertensives causing harmful cerebral hypoperfusion is not consistently proven, for example in an RCT of 62 people with cerebrovascular small vessel disease intensive blood pressure lowering did not significantly reduce cerebral perfusion.^45^

**Statin withdrawal:** There is a very limited literature on the effects of statin withdrawal. A 2016 Cochrane review on statin withdrawal in patients with dementia found no suitable studies addressing this question.^46^ Notably, in an RCT on statin withdrawal in patients with a short life expectancy of less than 1 year, without a recent history of cardiovascular disease (22% were cognitively impaired), patients in the discontinuation group had slightly improved quality of life.^47^

### Diagnosis

#### **PICO 6:** In patients with stroke, **does routine use of cognitive screening**, compared to no routine screening, improve stroke care?

##### Analysis of the current evidence

In this PICO, we consider cognitive assessment, in particular short screening tests, following stroke as an intervention, that is, does routine screening of stroke survivors improve outcomes? For the purposes of this PICO, we considered any point in the stroke pathway. However, we were particularly interested in cognitive screening performed in the acute setting as such screening is recommended in many international stroke best practice statements and clinical guidelines.^48^ Our intention was not to assess the benefits of clinician directed, targeted cognitive assessment, but rather to assess policies of routine, standardised screening of all stroke survivors. For consistency of language, we differentiate screening from more comprehensive assessments or diagnostic formulations.

We pre-specified three questions with separate outcomes of interest: (1) does cognitive screening increase the detection of later cognitive syndromes in clinical practice? (2) does cognitive screening change subsequent care pathways? and (3) does cognitive screening translate into health economic benefits? For this PICO, we only considered studies that used randomised or quasi-randomised trial designs.

Although there are many articles describing the diagnostic properties of cognitive screening tools in stroke, we found relatively few articles that assessed whether this cognitive screening made a difference to patient care pathways or outcomes. We found no trials that described outcomes relating to diagnosis or the components of stroke care. One study (Forster 2009)^49^ assessed resource use as a secondary outcome and is considered further in the additional information section, but as this study used a multicomponent assessment strategy that could include, but did not mandate, cognitive screening, it does not meet our PICO inclusion criteria.

##### Additional Information

We found four trials that were relevant to the topic but not completely aligned with our original question. The trials had differing populations, interventions and outcomes, so we did not attempt a quantitative summary. The trials had similar methodological limitations and highlight the difficulty in trials of cognitive screening. As stroke survivor participants had to provide informed consent and had to be able to complete the relevant assessments, included populations were not representative of unselected stroke survivors. There were issues with attrition, for example in the OCS CARE trial,^50^ 821 were randomised but outcomes were only available for 467 (57%). All the trials were under-powered to detect small but meaningful differences in important secondary outcomes like caregiver burden or satisfaction with care.

The OCS CARE trial^50^ randomised post-acute stroke survivors to domain-specific cognitive screening using the Oxford Cognitive Screen (OCS) or general cognitive screening using the Montreal Cognitive Assessment (MoCA). At 6 months, there was no difference in stroke impairments or health related quality of life.

McKinney et al.^51^ randomised 228 4-week, stroke survivors to a bespoke, staged neuropsychological battery or usual cognitive screening. At 6 months, there was no difference in function, mental health or satisfaction with care, although there was a trend towards reduced caregiver strain.

Forster et al.^49^ randomised 265 stroke survivors at 3 months to a bespoke assessment package that was not exclusively focused on cognition but could include cognitive assessment where indicated. At 1 year follow-up, there was no improvement in function, but a trend towards improvement in secondary outcomes of caregiver strain, satisfaction with care and healthcare costs.

Arts et al.^52^ described a pilot of an outpatient physical and cognitive testing programme for minor stroke. Of 42 recruited, 38 received the intervention and reported increased satisfaction but no difference in measures of function, mood or quality of life.

We found a protocol for an ongoing trial (ECO-stroke)^53^ of a multicomponent assessment administered when stroke survivors return home. The study will include measures of clinical effectiveness, cost-effectiveness and process evaluation.

In assessing the evidence for this PICO question and for the other diagnosis themed PICO questions in this guidance, there are certain contextual factors that require consideration. When cognitive testing is used it can have differing purposes. For example, in acute stroke care a brief assessment can inform whether a person is at risk of cognitive problems and likely to require more detailed cognitive assessment later in the admission. This could be termed cognitive triage, or screening and screening is the term preferred in this guidance. A more detailed assessment may be used to inform a diagnostic formulation, this process is often referred to as cognitive assessment. In research, cognitive tests may be used as outcome measures, a process that is neither screening nor assessment.

This PICO did not consider neuropsychological assessment which allows for a comprehensive characterisation of cognitive strengths and weaknesses, emotional and behavioural changes post-stroke, and biopsychosocial case formulation to inform a range of management recommendations and treatment pathways.

For our PICO, we included those outcomes rated as critical by the writing group. As cognitive screening is a system based intervention, we prioritised outcomes at the population level. We recognise that we did not include directly patient focussed outcome measures such as acceptability and feasibility, but these would be important considerations for any cognitive screening programme.

The preferred properties of a cognitive test will differ depending on the purpose of that test. For example, in the case of a brief screening tool where a positive result may trigger a more detailed assessment, it could be argued that the imperative is to detect as many people with possible cognitive problems as possible even if this risks unnecessary additional testing for some. In this case sensitivity may be preferred over specificity.

Related to this point, the potential consequences of a false positive and false negative diagnosis should also be considered. The implications of missing prevalent cognitive issues (false negative) could include not being referred for treatment. Whereas wrongly labelling a person as having cognitive issues risks worry and further unnecessary testing. The balance of harms will vary in differing healthcare settings and it is difficult to be prescriptive when offering general guidance.

#### **PICO 7:** In patients with stroke (acute or post-acute), what is the accuracy of **Montreal Cognitive Assessment** for contemporaneous diagnosis of post-stroke cognitive impairment or dementia?

##### Analysis of the current evidence

For this PICO, and subsequent PICOs in this Diagnosis section, we will describe accuracy of tests rather than efficacy, and we will focus on those cognitive screening tools prioritised by the module writing group. We will use the terminology favoured in test synthesis literature,^54^ that is, ‘diagnostic test accuracy’, but we recognise that the tools we describe are not diagnostic in their own right. While we refer to these questions using the PICO terminology, our questions on screening tools are considering accuracy rather than comparative efficacy of interventions, so in formulating these questions our concepts of interest were the index test (screening tool), reference standard and condition of interest (in this case post-stroke cognitive impairment or dementia).

In clinical practice, a cognitive screening tool is usually used, directly or indirectly, to inform a management decision. For example, a person with recent stroke who scores poorly on a multidomain screening tool may be referred for more detailed assessment that will guide subsequent rehabilitation.^55^ However, PICO 6 has shown that there is limited evidence around the test-treatment-outcome paradigm for cognitive testing in stroke. Therefore, to help the clinician choose the most appropriate assessment for a given clinical context, an analysis of the test’s properties with a focus on metrics such as sensitivity and specificity can be useful.^56^

The methods underpinning the test accuracy synthesis differ in some regards from the standard synthesis of trials. In particular, the application of GRADE to diagnostic test accuracy is not as well developed as it is for synthesis of intervention studies. In our GRADE assessment, we considered risk of bias and applicability using the QUADAS-2 tool,^57^ we considered internal consistency through visual inspection of forest plots and considered the precision of the summary estimate. More detailed descriptions of test accuracy synthesis and reporting are available from Cochrane^58^ and others.

The Montreal Cognitive Assessment (MoCA) is a brief screening tool used to detect mild cognitive impairment and dementia and has been used extensively across research settings and clinical groups, including stroke survivors.^59^ MoCA assesses a number of cognitive domains, including executive function, memory, attention, language and orientation to provide a test score of global cognitive function. However, the MoCA has been criticised due to the necessity of intact visuospatial and language function to complete the assessment.^60^

We identified 17 studies^61-77^ that assessed the diagnostic test accuracy of the MoCA across a number of settings (e.g. acute, rehabilitation, outpatient and community) in a stroke population. Stroke aetiology was mixed (9 studies), ischaemic (7 studies) or not reported (1 study). The time since stroke onset varied considerably across studies, from <2 days to >12 months. The reference standard was clinical diagnosis of post-stroke cognitive impairment/dementia in five studies, cognitive impairment as defined by a neuropsychological test battery (11 studies) or both (1 study).

We performed meta-analyses to give summary estimates of the sensitivity and specificity, using bespoke software.^78^ It should be noted that across studies, test properties were described at varying cut-offs of the assessment scale, and our summary estimates are for those cut-off points that were most common across studies. The majority of articles had a high risk of bias. Limitations included non-consecutive sampling of stroke survivors, study heterogeneity and unblinded interpretation of either the index test or reference standard. Similarly, little information was provided on incomplete or missing data (Supplementary Materials).

We recognise that using screening tool threshold scores to make a cognitive classification is a reductionist approach. At the individual patient level, scores should be interpreted in the context of education, cultural background, language and many other factors. However, the threshold score approach is commonly used in practice and research and so we assessed the test properties of MoCA at varying thresholds.

**Figure 3.**
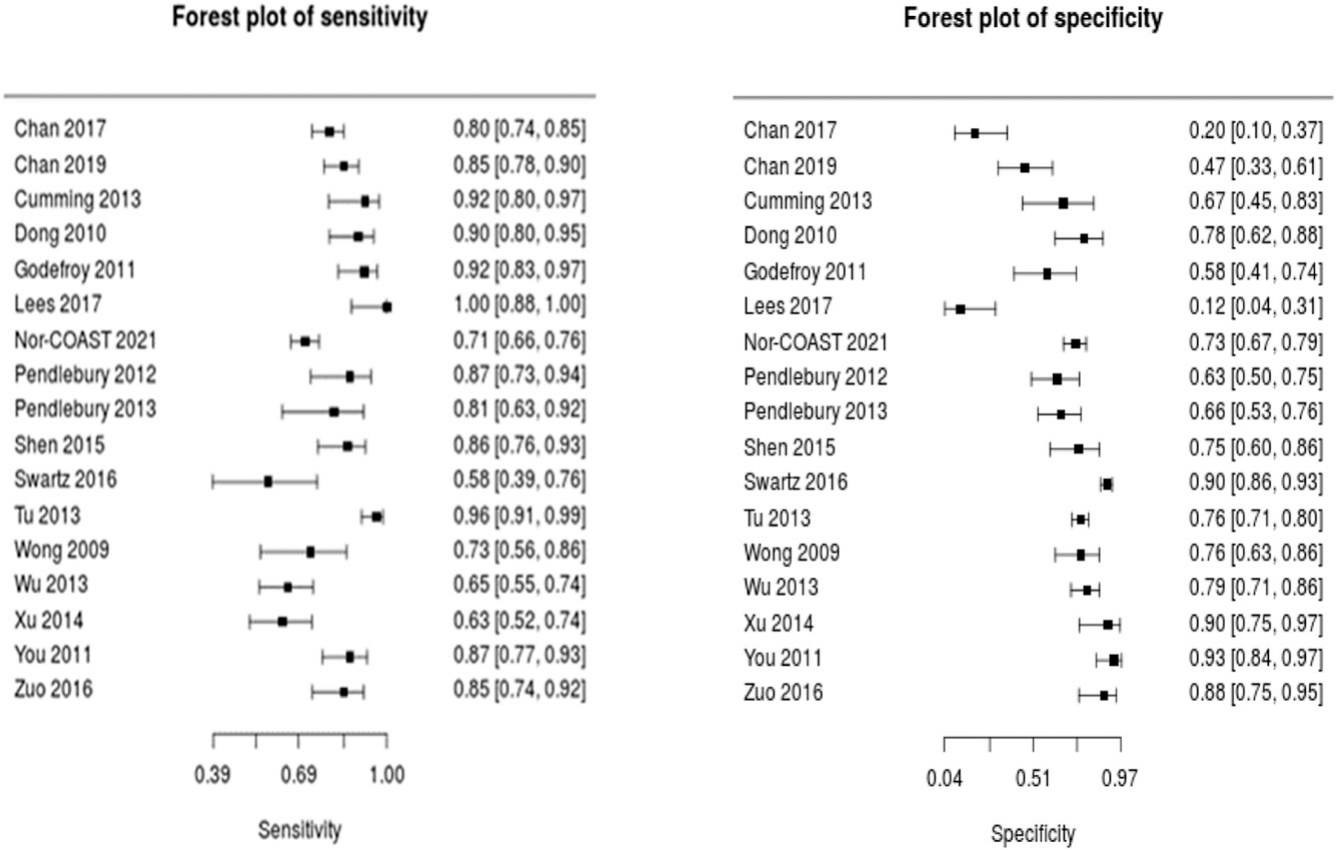
Forest plots describing test accuracy (sensitivity and specificity) studies of Montreal Cognitive Assessment. Summary estimates (random effects model) and corresponding 95% confidence intervals are given in the Summary of Findings table.

Our summary analyses suggest a common pattern of test properties for the MoCA when used in a stroke population with sensitivity favoured over specificity. Table 4 shows our GRADE assessment of the diagnostic accuracy of MoCA for contemporaneous diagnosis of post-stroke cognitive impairment. Across 17 studies, using the best fit sensitivity and specificity threshold if more than one threshold was reported and irrespective of the timeframe of cognitive screening, sensitivity was 0.84 and specificity was 0.71 (see Figure 3). At the lower MoCA, threshold of 21–23 sensitivity was 0.84 and specificity 0.78. A higher cut-off of 24–26 has similar sensitivity of 0.86 but somewhat lower specificity of 0.59. For initial screening of cognition, these properties could be considered acceptable; however, the MoCA is not a substitute for clinical diagnostic assessment.

**Table 4.** Summary of findings for PICO 7. Assessment of the diagnostic accuracy of Montreal Cognitive Assessment for contemporaneous diagnosis of post-stroke cognitive impairment or dementia.

Participants: Stroke survivors Settings: Variety (acute and post-acute) Intervention: Montreal Cognitive Assessment Reference standard: Clinical dementia diagnosis or multidomain impairment				
Test	Summary sensitivity Summary specificity	N participants/N with dementia	Risk of bias	GRADE
MoCA at best performing reported threshold	Sens: 0.84 (0.78–0.89) Spec: 0.71 (0.59–0.81)	Seventeen studies 2999 participants 1428 PSCI	High	Low^a^
MoCA ‘acute’ time period	Sens: 0.86 (0.80–0.90) Spec: 0.61 (0.43–0.76)	Ten studies 1518 participants 991 PSCI	High	Low^a^
MoCA ‘post-acute’ time period	Sens: 0.86 (0.74–0.94) Spec: 0.80 (0.66–0.89)	Five studies 885 participants 318 PSCI	High	Low^a^
MoCA threshold 22 (+/−1)	Sens: 0.84 (0.72–0.92) Spec: 0.78 (0.64–0.88)	Ten studies 1327 participants 541 PSCI	High	Low^a^
MoCA threshold 25 (+/−1)	Sens: 0.86 (0.78–0.92) Spec: 0.59 (0.46–0.72)	Seven studies 1672 participants 887 PSCI	High	Low^a^

^a^ Downgraded due to risk of bias and limited precision.

Acute refers to less than 3 months since stroke. PSCI = Post-stroke cognitive impairment (including post-stroke dementia), PICO: Population, Intervention, Comparator, and Outcome, MoCA: Montreal Cognitive Assessment (MoCA).

While sensitivity was consistent across the reported cut-off points, specificity was lower for the higher cut-off of 24–26, suggesting that the lower MoCA cut-off of 21–23 has improved overall test properties for post-stroke cognitive impairment. Similarly, our analysis suggests that the MoCA has better diagnostic test accuracy when used in the post-acute (>3 months post-stroke) than acute phase. However, there was a common issue across studies of inappropriate exclusion of patients with moderate/severe aphasia or of those who lack the ability to consent, which leaves potential for bias. Therefore, we recommend due caution in the interpretation of these findings.

##### Additional information

Diagnostic test accuracy of the MoCA in stroke has been the subject of a number of systematic reviews. Lees et al.^79^ reviewed the test accuracy of various cognitive screening tools for dementia or multidomain cognitive impairment after stroke. In examining the MoCA, pooled data from six studies which used the cut-off <22/30 reported sensitivity 0.84 and specificity 0.78. A higher cut-off (<26/30) had a lower specificity of 0.45 but a higher sensitivity of 0.95. These results are broadly in keeping with our synthesis, albeit our more contemporary review has a greater number of studies included.

Reviews of MoCA in non-stroke settings are available, and the pattern of higher sensitivity and lower specificity is consistent across studies.^80^ It should be remembered that the MoCA was developed to assess for mild cognitive impairment in community-dwelling older adults and was not originally intended for use in acute stroke. There is a literature describing issues with feasibility of assessment when MoCA is applied in the acute stroke setting.^81^ Non-cognitive impairments can compromise completion of the MoCA, and research teams have adopted various approaches for handling partial or fully incomplete MoCA assessments.^74^ A recent development with application of MoCA is the need for mandatory training with associated training costs. It remains to be seen whether this will change the patterns of MoCA use in practice and research.

#### **PICO 8:** In patients with stroke (acute or post-acute), what is the accuracy of **Folstein’s Mini-Mental State Examination** for contemporaneous diagnosis of dementia?

##### Analysis of current evidence

In this PICO question, we describe the accuracy of Folstein’s Mini-Mental State Examination (MMSE)^82^ when used in the stroke context. The synthesis of test accuracy data is different to that of the standard intervention review. A discussion of the methods that underpin our approach is provided in PICO 7.

MMSE was developed as a screening test for dementia over 40 years ago and has also been widely used as an outcome measure in therapeutic studies. It consists of a number of items, with total possible score of 30, covering domains of orientation, memory and praxis. MMSE has been criticised because it does not assess executive function or language in detail.^83^

We found 16^62-64,70,71,74,75,84-92^ studies that had assessed the test accuracy of MMSE, six against a clinical diagnosis and 10 against a neuropsychological test battery with the reference standard being dementia (4 studies), cognitive impairment (9 studies) or both (2 studies). Stroke aetiology was mixed (9 studies), ischaemic (5 studies) or not reported (2 study). Study setting varied and included acute inpatient, outpatient, community and rehabilitation services. Time since stroke was also variable between studies, ranging from less than 7 days to over a year, and study size ranged from 51 to 300.

Using the QUADAS-2 tool,^57^ we found that all articles had a high risk of bias. Limitations included non-consecutive sampling of stroke survivors, study heterogeneity, handling of missing data and unblinded interpretation of either the index test or reference standard (Supplementary Materials).

We performed meta-analyses to give summary estimates of the sensitivity and specificity. It should be noted that across studies, test properties were described at varying cut-offs of the assessment scale, and our summary estimates are for those cut-offs points that were most common across studies. The need for caution in applying standardised thresholds at the individual patient level were discussed in PICO 7 and also applied here.

**Figure 4.**
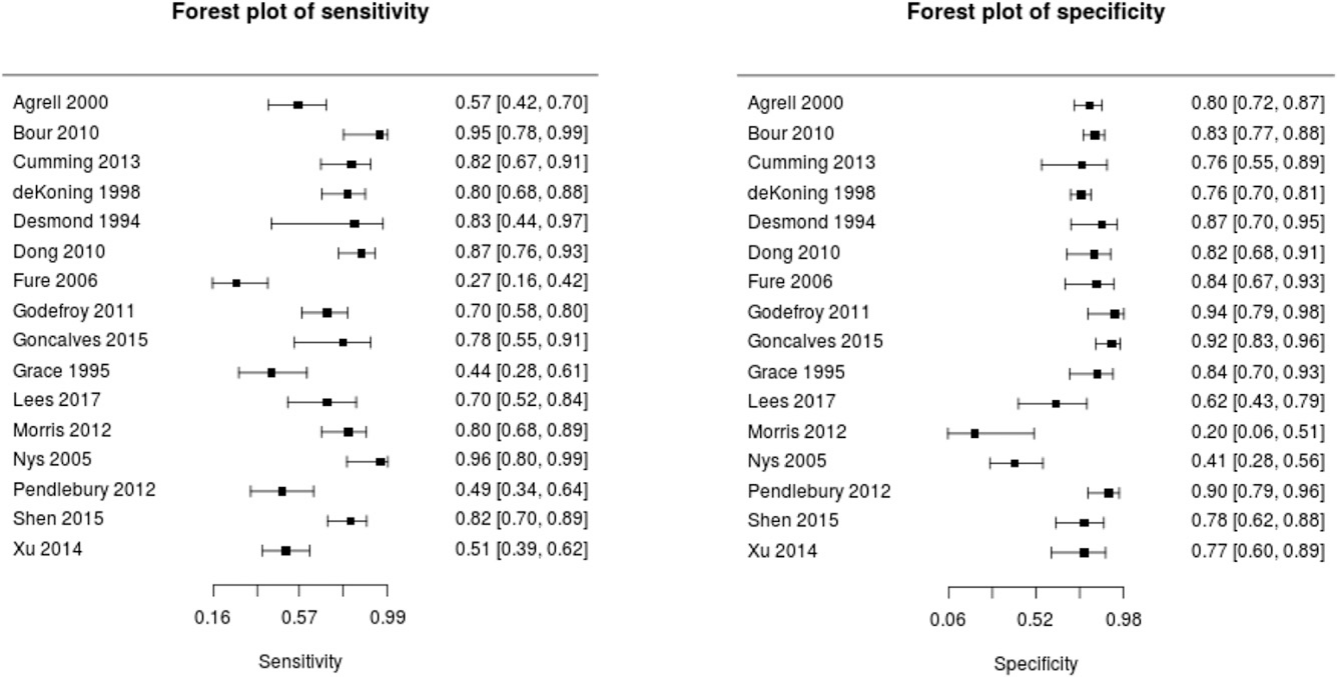
Forest plots describing test accuracy (sensitivity and specificity) studies of Folstein’s Mini-Mental State Examination. Summary estimates (random effects model) and corresponding 95% confidence intervals are given in the Summary of Findings table.

Table 5 shows the summary estimates of sensitivity and specificity. Across 16 studies, using the best fit sensitivity and specificity threshold if more than one threshold was reported and irrespective of the timeframe of cognitive screening, sensitivity was 0.73 and specificity was 0.62 (see Figure 4). At the standard MMSE thresholds of 22–24 sensitivity was 0.68 and specificity 0.82. Higher cut-offs of 25–27 had similar performance with marginally lower specificity (sensitivity 0.70 and specificity 0.76).

**Table 5.** Summary of findings for PICO 8. Assessment of the diagnostic accuracy of Folstein’s Mini-Mental State Examination for contemporaneous diagnosis of post-stroke cognitive impairment or dementia.

Participants: Stroke survivors Settings: Variety (acute and post-acute) Intervention: Mini-Mental State Examination (MMSE) Reference standard: Clinical dementia diagnosis or multidomain impairment				
Test	Summary sensitivity Summary specificity	N participants/N with dementia	Risk of bias	GRADE
MMSE all studies at ‘best’ performing reported threshold	Sens: 0.73 (0.62–0.82) Spec: 0.79 (0.72–0.85)	Sixteen studies 1655 participants 660 PSCI	High	Low^a^
MMSE ‘acute’ time period	Sens: 0.80 (0.66–0.89) Spec: 0.74 (0.59–0.85)	Nine studies 806 participants 393 PSCI	High	Low^a^
MMSE ‘chronic’ time period	Sens 0.60 (0.46–0.72) Spec 0.81 (0.75–0.86)	Four studies 651 participants 211 PSCI	High	Low^a^
MMSE threshold 23 (+/−1)	Sens: 0.74 (0.58–0.85) Spec: 0.82 (0.78–0.86)	Seven studies 704 participants 257 PSCI	High	Low^a^
MMSE threshold 26 (+/−1)	Sens: 0.72 (0.56–0.84) Spec: 0.76 (0.62–0.86)	Nine studies 951 participants 403 PSCI	High	Low^a^

^a^Downgraded due to risk of bias and precision.

Acute refers to less than 3 months since stroke.

PSCI = Post-stroke cognitive impairment (including post-stroke dementia).

Sensitivity and specificity clearly varied according to the cut-off chosen, but there was a consistent picture of generally higher specificity but lower sensitivity, with sensitivity slightly higher and specificity lower for acute rather than chronic time periods. Despite the clinical heterogeneity and potential bias issues, studies gave consistent findings across several settings.

##### Additional information

The MMSE has been the focus of previous reviews, for example, Lees et al. (2014)^79^ reviewed cognitive screening tests for dementia or multidomain cognitive impairment after stroke, based on a literature search in Jan 2014. They pooled data from 12 studies which used the MMSE and with cut-off <27/30 reported sensitivity 0.88 and specificity 0.62. A lower cut-off (<25/30) had lower sensitivity but higher specificity (0.71 and specificity 0.85).

Diagnostic test accuracy reviews and meta-analyses of MMSE are available for non-stroke populations.^93,94^ Test accuracy metrics are broadly similar to those reported in our stroke analysis. These reviews conclude that MMSE may have utility for assessing possible dementia but is less useful for assessing for mild cognitive impairment. Even for the assessment of dementia, MMSE is imperfect and not a substitute for detailed clinical assessment.

Most test accuracy analyses have considered screening tools in isolation. This is partly because of the lack of studies comparing two test strategies in the same population. For the clinician faced with multiple test options, the question of importance is often ‘which test is better’. A recent review used a network approach to indirectly rank the test properties of MoCA and MMSE in the stroke setting. Using this approach, MoCA at threshold <26/30 appeared to have the best true positive rate, whereas MMSE at threshold <25/30 appeared to have the best true negative rate.^95^ The most appropriate test in a particular situation will depend on the relative consequences of false positive and false negative screening results.

The MMSE has similar feasibility issues as described for the MoCA, particularly with regards to acute assessment when a patient is unwell or has stroke related impairments.^60^ MMSE has copyright restrictions and is not free to use for all, some centres no longer use the test routinely for this reason.

#### **PICO 9:** In patients with stroke (acute or post-acute), what is the accuracy of **Addenbrooke’s Cognitive Examination (ACE)** for contemporaneous diagnosis of dementia?

##### Analysis of the current evidence

In this PICO question, we describe the accuracy of the various iterations of the Addenbrooke’s Cognitive Examination (ACE)^96^ when used in the stroke context. The synthesis of test accuracy data is different to that of the standard intervention review. A discussion of the methods that underpin our approach is provided in PICO 7.

The Addenbrooke’s Cognitive Examination (ACE) was originally developed to overcome some of the recognised limitations of the MMSE by being more sensitive to mild dementia and able to differentiate between dementia subtypes, specifically Alzheimer’s disease and frontotemporal dementia. Subsequent adaptations of the ACE include the Addenbrooke’s Cognitive Examination-Revised (ACE-R) and ACE-III).^97,98^ The ACE has 21 questions, covering five different cognitive domains: attention/orientation, memory, language, verbal fluency and visual perceptual/visuospatial skills. The total score is 100, and the thresholds used to diagnosis dementia are typically 82/83 or 88.

We found four studies^65,74,88,90^ that assessed the accuracy of versions of the ACE in stroke, two used clinical diagnosis and two used a neuropsychological test battery with reference standard being dementia (1 study) or cognitive impairment (3 studies). The four studies identified varied in study setting and included acute inpatient, community and rehabilitation services. Time since stroke was variable between studies, ranging from less than 18 days to >12 months, and study size ranged from 18 to 91.

Using the QUADAS-2 tool,^57^ we found that all studies had high risk of bias. Limitations included study heterogeneity, unblinded interpretation of either the index test or reference standard and handling of missing data (Supplementary Materials).

Given the heterogeneity in test content, application, scoring and setting, we did not attempt a meta-analysis of ACE test accuracy data. Table 6 describes the sensitivity and specificity of the four studies for a range of thresholds. Sensitivity and specificity varied across studies and according to the threshold chosen, with sensitivity being higher and specificity lower for higher thresholds. The need for caution in applying standardised thresholds at the individual patient level were discussed in PICO 7 and also applied here. Our overall GRADE assessment was of very low-quality of evidence due to heterogeneity, inconsistency, imprecision and risk of bias.

**Table 6.** Summary of findings for PICO 9. Assessment of the diagnostic accuracy of iterations of the Addenbrooke’s Cognitive Examination (ACE) for contemporaneous diagnosis of post-stroke cognitive impairment or dementia.

Participants: Stroke survivors Settings: Variety (acute and post-acute) Intervention: Addenbrookes Cognitive Examination Reference standard: Clinical dementia diagnosis or multidomain impairment						
Study	ACE version Diagnostic cut-off	Setting	N with PSCI	Accuracy	Risk of bias	GRADE
Morris et al. (2012)	ACE-R <75 ACE-R <82 ACE-R <88	Acute inpatient	51/61 (84%)	Sens 0.59 Spec 0.40 Sens 0.80 Spec 0.40 Sens 0.90 Spec 0.20	High	Very low^a^
Pendlebury et al. (2012)	ACE-R <88 ACE-R <90 ACE-R <92	Community (stroke and TIA)	39/91 (42%)	Sens 0.56 Spec 100 Sens 0.67 Spec 0.98 Sens 0.72 Spec 0.79	High	
Goncalves et al. (2015)	ACE-R <72–73	Neurology department	18/18 (100%)	Sens 100 Spec 0.92	High	
Lees et al. (2017)	ACE-III <82	Rehabilitation unit	27/51 (53%)	Sens 0.93 Spec 0.11	High	

^a^Downgraded due to risk of bias, inconsistency and imprecision.

ACE-R: Addenbrooke’s Cognitive Examination-Revised.

##### Additional information

There are reviews of the test properties of various iterations of ACE in non-stroke settings. The most recent review reports a limited literature on the accuracy of the newer versions of the test.^99^ Where data are available, there is a pattern of sensitivity and specificity varying across studies and thresholds used to define test positive results, with sensitivity being higher and specificity lower for higher thresholds. These results are similar to those seen in our stroke accuracy synthesis.

There is less published literature on feasibility and acceptability of ACE based assessment in stroke settings. The ACE is a longer test than MMSE and MoCA although offers a more detailed assessment, thus it would not seem suitable for use in a time pressured acute environment. In one of the articles that included both ACE and MoCA, the ACE had a longer administration time, but this did not improve the accuracy compared to MoCA.^74^ ACE is available for use at no cost to the user. Free to access training is available, for example: https://www.mvls.gla.ac.uk/aceiiitrainer/register.aspx, but no particular training programme is mandated by the test developers.

#### **PICO 10.** In patients with stroke (acute or post-acute), what is the accuracy of the **Oxford Cognitive Screen (OCS)** for contemporaneous diagnosis of dementia?

##### Analysis of the current evidence

In this PICO question, we describe the accuracy of the Oxford Cognitive Screen (OCS)^100^ when used in the stroke context. The synthesis of test accuracy data is different to that of the standard intervention review. A discussion of the methods that underpin our approach is provided in PICO 7.

The Oxford Cognitive Screen (OCS) has been specifically developed to screen for domain-specific cognitive impairments after stroke. The OCS consists of 10 subtests that screen for impairments in five domains: language, attention, memory, praxis and numeric cognition. As the primary aim of the OCS is to detect domain-specific post-stroke impairments and not dementia, the OCS has been validated for this specific purpose.

We did not identify any studies that were aligned with our test accuracy paradigm of comparing OCS to a reference standard diagnostic formulation based on clinical assessment and/or detailed neuropsychological battery. The lack of published data may reflect the rationale that motivated development of the OCS, to move away from dichotomous assessments of impaired/non-impaired and offer clinicians a domain-by-domain summary of the presence and severity of cognitive impairments.

##### Additional information

We identified three studies that investigated the sensitivity and specificity of the OCS subtests relative to single-test reference standards for domain-specific impairment.^100-102^ In addition, we identified two studies investigated the ability of the OCS to discriminate stroke patients from healthy controls.^103,104^ All these data suggest that OCS can offer valid domain-specific assessment. However, while these methods of validation are appropriate, they do not answer our question of interest around test accuracy for cognitive syndromes. In particular, the accuracy of the reference standards used in these studies are debatable and discrimination of stroke survivors and healthy controls is not necessarily a good proxy for discriminating presence and absence of domain-specific cognitive impairment.

The OCS was designed to be inclusive for stroke patients. Multiple choice options are provided so that patients with expressive language difficulties can provide responses whenever possible. Executive function is evaluated with a trail making test that does not require intact alphanumeric knowledge. In addition, stimuli are presented centrally in the visual field as much as possible so that patients with visuospatial difficulties can complete the test. Two studies have suggested that this inclusive design translates into better completion rates relative to the MoCA and MMSE.^101,105^ For example, in an Italian study of sequential admissions to stroke rehabilitation, OVCS could not be fully completed in three of 325 patients, while MMSE was not possible in six.^101^ It should be noted that compared to the other tests considered (MoCA, MMSE and ACE) the studies describing properties of OCS are less biased by exclusion of stroke survivors with deficits that may interfere with testing. The OCS is available free of charge for all clinical use and publicly funded research. Online, free to access training in administration is available (https://www.ocs-test.org/how-to/).

#### **PICO 11.** In patients with stroke (acute or post-acute), what is the accuracy of **remote assessment** for contemporaneous diagnosis of dementia?

##### Analysis of the current evidence

In this PICO question, we describe the accuracy of remote (not in-person) cognitive assessment when used in the stroke context. Remote assessment could include telephone, video-based or real time online assessment. We did not include postal questionnaires in the remit. The synthesis of test accuracy data is different to that of the standard intervention review. A discussion of the methods that underpin our approach is provided in PICO 7. We used the search strategy and synthesis of a recent review on the topic of telephone cognitive screening and extracted the articles specific to stroke.^106^

Various cognitive screening tools have been described that can be used over the telephone or video conferencing platforms. We found four articles describing the accuracy of three different telephone-based tests in a stroke population.^87,107-109^ We found no suitable papers describing video-based cognitive assessment for diagnosis of dementia following stroke.

In general, the articles had low risk of bias, but the varying proportions with dementia suggest that not all the populations studied are applicable to real world stroke practice.

We did not perform meta-analysis to give a summary estimate of test accuracy, due to the small number of studies and heterogeneity in the tests. Importantly, even when tests are described by the same name, they may have differing content. This is not unique to telephone assessment, for example, tests described as ‘short-form MoCA’ differ in the component items across the included studies.^110^

Pendlebury et al. described the performance of three telephone screening tools—the Telephone Interview for Cognitive Status (TICS), the telephone-based Montreal Cognitive Assessment (t-MoCA) and a shortened version of the t-MoCA.^107^ Across 68 stroke survivors, there was a pattern of high sensitivity for detection of multidomain cognitive problems, but lower specificity. Zietemann et al.^108^ described the performance of the TICS and t-MOCA in 105 participants of the DEDEMAS (Determinants of dementia after stroke) cohort. Both tests had reasonable sensitivity, but the t-MOCA had better specificity. Wong et al. assessed the short form t-MoCA on 104 participants of the STRIDE (Stroke Registry Investigating Cognitive Decline) cohort.^109^ Desmond et al. assessed the TICS in 72 stroke survivors. In both studies there was reasonable accuracy with sensitivity better than specificity.^87^

Our summary analyses suggest a common pattern of test properties for the telephone-based screening tools when used in stroke. Sensitivity tends to be high, with lower specificity and no clearly superior test. This implies that telephone assessment using these tools will detect most stroke survivors with dementia but at the cost of false positive screening tests. The relative risks and benefits of false positive and false negative diagnoses need to be considered for the person being assessed. Patients with a false positive test may require further, more detailed cognitive assessment. Patients with a false negative diagnosis may miss early intervention, but at present there is no proven intervention. For initial screening or triage, these properties are acceptable, but the telephone assessment is not a substitute for clinical diagnostic assessment (Table 7).

**Table 7.** Summary of findings for PICO 11. Assessment of the diagnostic accuracy of iterations of remote (telephone) assessment for contemporaneous diagnosis of post-stroke cognitive impairment or dementia.

Participants: Stroke survivors Settings: Variety (mostly post-acute) Intervention: Telephone-based cognitive screening Reference standard: Clinical dementia diagnosis or multidomain impairment				
Test	Summary sensitivity Summary specificity	N participants/N with dementia	Risk of bias	Quality
Telephone Interview for Cognitive Status	Sens: 0.92 (0.59–0.99) Spec: 0.67 (0.49–0.81)	Three studies 242 participants 26 dementia	High	Very low^a^
Telephone-based Montreal Cognitive Assessment	Sens: 0.98 (0.25–1.00) Spec: 0.73 (0.43–0.91)	Two studies 169 participants 20 dementia	High	Very low^a^
Short form of t-MoCA	Sens: 0.93 (0.59–0.99) Spec: 0.63 (0.46–0.78)	Two studies 172 participants 63 dementia	Unclear	Very low^a^

^a^Downgraded due to risk of bias, inconsistency, imprecision and indirectness.

t-MoCA: Telephone-based Montreal Cognitive Assessment.

##### Additional information

With the social distancing and other restrictions imposed by the Covid-19 viral pandemic, remote assessment of stroke survivors is increasingly used in research and in clinical practice. While the literature on stroke-specific remote cognitive assessment is limited, there is a more robust evidence base for telephone assessment of general and older adult populations. A recent review found 34 articles describing 15 different telephone-based cognitive assessments.^106^ TICS was the most studied assessment tool and properties in older adults were similar to those seen in stroke, with high sensitivity and lower specificity. However, properties could be altered by changing the threshold that defines a ‘positive’ test. This review identified limitations of telephone assessment that are relevant to stroke populations. Telephone testing makes assessment of visual-spatial function more difficult than in-person, pencil and paper testing. In addition, the feasibility of telephone testing may be reduced when used with people who have hearing impairment.

There is less supporting literature around video-based cognitive assessment. A recent review found 12 studies that included mixed populations and compared video to standard in-person assessment.^111^ The review authors reported that performance on certain tests was different when using a video-based platform, although differences were modest and may not have clinical importance. They concluded that best practice guidance is needed for video-based cognitive screening. A study of stroke survivors comparing in-person and video-based MoCA performance reached similar conclusions.^112^

### Treatment

#### **PICO 12:** In people with post-stroke cognitive impairments, do **cholinesterase inhibitors**, compared to placebo, delay cognitive decline or progression to dementia; improve behavioural and psychological symptoms, decrease caregiver burden and/or cause adverse effects?

##### Analysis of the current evidence

In this section we consider treatments for stroke survivors with an established cognitive syndrome, either post-stroke cognitive impairment or dementia. Currently, there is no pharmacological treatment approved for post-stroke cognitive impairment. Efficacy of cholinesterase inhibitors in mild to moderate Alzheimer’s disease is established, and donepezil, galantamine and rivastigmine are approved for symptomatic treatment in Alzheimer’s and other dementia types.^113-115^ Here, we aimed to evaluate the potential utility of cholinesterase inhibitors in post-stroke cognitive impairment. We pre-specified outcomes of interest relating to cognitive decline, behavioural and psychological symptoms (BPSD), caregiver burden and adverse effects (AEs).

We found several trials of cholinesterase inhibitor in vascular dementia but only one trial with a specific focus on post-stroke cognitive impairment. Narasimhalu et al.^116^ described the effect of oral rivastigmine titrated up to 9 mg/day (4.5 mg oral twice daily) in 50 participants with a history of recent stroke (25 patients in each arm) who had evidence of post-stroke cognitive impairment without criteria of dementia at randomisation. There was no benefit of rivastigmine across the primary outcomes (executive functions). There were no differences concerning global cognitive evaluation, function and activities of daily living, behavioural and psychological symptoms. There were no relevant adverse events reported. Impact on caregiver outcomes was not studied. The study was low risk of bias across all domains but with a single, under-powered study there were serious concerns over precision and publication bias (Table 8).

**Table 8.** Summary of findings for PICO 12. Assessment of cholinesterase inhibitors for post-stroke dementia.

Participants: Post-stroke cognitive impairment Settings: At least 6/12 following stroke Intervention: Rivastigmine Comparator: Placebo				
Outcome	No of participants	Effect placebo (*n* = 25)	Effect intervention (*n* = 25)	Quality of evidence (GRADE)
Cognition (ADAS-Cog)	One trial, *n* = 50 (25 in each arm)	Mean change from baseline: −2.8 (−5.1 to 0.3)	Mean change from baseline: −0.6 (−3.1 to 1.9)	Very low^a^
BPSD (neuropsychiatric inventory)	One trial, *n* = 50 (25 in each arm)	Mean change from baseline: 0.1 (−2.6 to 2.9)	Mean change from baseline: −0.31 (−0.9 to 0.9)	Very low^a^
Adverse events	One trial, *n* = 50 (25 in each arm)	N (%) with AE 10 (40%)	N (%) with AE 9 (36%)	Very low^a^

^a^Downgraded due to serious imprecision; publication bias.

##### Additional information

We found seven randomised trials describing the use of cholinesterase inhibitors in vascular dementia (donepezil, three trials *n* = 2193^117-119^; rivastigmine, two trials *n* = 750^120,121^ and galantamine, two trials *n* = 1380^122,123^). While most of the trials assessed adverse events and cognitive outcomes, very few evaluated behavioural effects, and none assessed the impact on caregiver related outcomes. Some of those studies included patients with previous stroke,^118,119,120,123^ so these data are relevant to our PICO question, but subgroup analysis restricted to participants with stroke was not possible. Precise subtyping of dementia is difficult and in older adults mixed pathologies are common, so the interpretation of data in a ‘vascular’ dementia review needs to be mindful of this. One open trial with 73 patients studied caregiver reported outcomes in multi-infarct dementia, but the outcomes of interest for our analysis were not evaluated.^124^ A recent Cochrane review performed network meta-analysis of trials using cholinesterase inhibitors (including the Narasimhalu trial of a post-stroke population) and found varying quality evidence that donepezil and galantamine may improve cognition compared to placebo, but the effect may not be sufficiently large to be clinically important.^125^ There was low certainty evidence that rivastigmine had no significant effect on cognition. There was moderate certainty evidence that donepezil at higher dose and galantamine may increase adverse events but not serious adverse events.

We found one trial of donepezil used in the monogenic condition cerebral autosomal dominant arteriopathy with subcortical infarcts and leucoencephalopathy (CADASIL) (168 participants, a proportion had previous stroke or transient ischaemic attack).^126^ This condition offers a model of pure vascular dementia, due to cerebral small vessel disease, in a younger population unlikely to have co-existent age-related Alzheimer’s pathology. There was no significant difference in the primary cognitive endpoint of vascular AD assessment scale cognitive subscale (V-ADAS-cog) at 18 weeks. There were small but significant improvements in executive function, but these had no impact on instrumental activities of daily living. This suggested that even though there may be a small biological effect, treatment had no clinically meaningful effect.

Although with a lower degree of evidence compared to Alzheimer’s disease (based on a single study or in post-hoc analyses of Alzheimer’s disease or vascular dementia subgroups trials), utility of cholinesterase inhibitors has been reported for mixed dementia (Alzheimer’s disease plus vascular dementia).^127^

#### **PICO 13:** In people with post-stroke cognitive impairments, does **memantine** compared to placebo, delay cognitive decline or progression to dementia, improve behavioural and psychological symptoms, decrease caregiver burden and/or cause adverse effects?

##### Analysis of the current evidence

Memantine, a glutamate NMDA receptor antagonist is approved for use as a symptomatic treatment in moderate to severe dementia due to Alzheimer’s disease and can be used alone or added to cholinesterase inhibitors.^128^ We were interested in the potential utility of memantine in post-stroke cognitive impairment and we specified outcomes relating to cognitive decline, BPSD, caregiver burden and AE.

We found no study specifically describing the effect of memantine in post-stroke cognitive impairment without dementia.

##### Additional information

We found three studies of memantine in vascular dementia (*n* = 928). Two studies did not specifically consider post-stroke populations,^129,130^ and the third evaluated only language deficits.^131^ A recent Cochrane review found a probable small clinical benefit among patients with vascular dementia,^128^ it was not possible to assess the subgroup of participants with previous stroke. The review reported moderate to low-quality evidence that memantine may improve cognition and behaviour, but the differences were unlikely to be clinically important. There was high-quality evidence of an increase in total adverse events, but not serious adverse events, with memantine. Another meta-analysis considering, both memantine and cholinesterase inhibitors, focussed on cognitive outcomes specifically the Mini-Mental State Examination (MMSE) and described low potential efficacy of memantine when considering vascular dementia as subgroup.^132^

### **PICO 14:** In people with post-stroke cognitive impairments, do the nootropics **actovegin or cerebrolysin**, compared to placebo improve cognitive decline, improve behavioural and psychological symptoms, reduce caregiver burden and/or increase adverse events?

#### Analysis of the current evidence

Actovegin and cerebrolysin are animal-derived nootropics, that may have potential efficacy in the treatment of neurodegenerative disease.^133^ These agents are used in many countries for conditions such as dementia, stroke and traumatic brain injury, but unlike other drugs considered in this guideline (cholinesterase inhibitors and memantine), the nootropics do not have international approval for use in dementia. The mechanisms of action of the nootropics are not clear, but putative vascular effects have been described, so there is an assumption of a potential efficacy in vascular cognitive syndromes.^134^ We were interested in the potential effect of these agents in post-stroke cognitive impairment and specified outcomes relating to cognitive decline, BPSD, caregiver burden and AE.

We found one double blinded RCT of actovegin used in a post-stroke population exploring cognitive outcomes.^135^ The ARTEMIDA trial randomised 503 participants within 7 days after ischaemic stroke. The intervention consisted of daily infusions of actovegin for 20 days followed by oral actovegin for 6 months. Primary outcome was defined as a change in cognitive function measured through ADAS-Cog. A beneficial effect of actovegin compared to placebo was reported, but the effect size described may be less than the minimal clinically important difference. Other related outcomes (change in a global cognitive test, rates of incident dementia) did not show significant between group differences. The intervention involved daily intravenous infusions for up to 20 days, with associated cost and burden. More participants taking actovegin had to discontinue study drug (4.7% versus 8.4%). The most frequent adverse event was recurrent ischaemic stroke and there were higher absolute numbers of recurrent stroke events in those taking actovegin (14 vs 7 [absolute numbers]). While these differences were not ‘significant’ we felt there was sufficient signal of concern for these data to inform our Expert Consensus statement. The trial was low risk of bias, but as a single study we noted imprecision, inconsistency across the included cognitive outcomes and potential for publication bias. In formulating our recommendation, we considered efficacy, potential for harm and costs (Table 9).

**Table 9.** Summary of findings for PICO 14. Assessment of actovegin for post-stroke dementia.

Population: Recent ischaemic stroke Intervention: Actovegin daily intravenous then oral Comparator: placebo			
Outcome	No of participants	Difference in mean change from baseline at 6 months	Quality of evidence (GRADE)
Cognition (ADAS-Cog)	One trial, *n* = 196 (actogevin) vs 202 (placebo)	Mean change from baseline: −2.3 (−3.9, −0.7); *p* = 0.005	Very low^a^
Adverse events	One trial, *n* = 250 (actogevin) vs 253 (placebo)	% Discontinuing due to AE actovegin: 8.4%; placebo: 6.6%	Very low^b^
BPSD	No data		
Caregiver strain	No data		

^a^Downgraded due to imprecision; publication bias and inconsistency.

^b^Downgraded due to serious imprecision; publication bias and inconsistency.

We found reviews of trials of cerebrolysin when used in stroke and vascular dementia populations, but no trials with an exclusive focus on post-stroke cognitive impairment.^136,137^

##### Additional information

We found six trials (*n* = 597 participants) describing the use of cerebrolysin in vascular dementia. These data were summarised in a recent Cochrane review.^136^ This review included people living with post-stroke dementia, and so these data are relevant to our PICO question. The review found very low-quality evidence that cerebrolysin may improve cognition compared to placebo, but the effect may not be sufficiently large to be clinically important. There was very low-quality evidence that rates of serious adverse events were not different between cerebrolysin and placebo. Factors such as economic and opportunity cost (cerebrolysin needs to be administered as a frequent intravenous infusion) and longer term effects (most studies followed participants for weeks to months only) were not considered in the Cochrane review but are important for decision making.

We found seven trials (*n* = 1601 participants) describing cerebrolysin in acute stroke and these were described in a recent review.^137^ The review found moderate quality evidence that cerebrolysin had no effect on mortality, but the intervention was associated with possible increased adverse event rates. We are aware of trials of cerebrolysin as an adjunct to motor rehabilitation following stroke, but we considered these out of the scope of this review.^138^

We found a recent review describing actovegin in acute stroke, but with exception to the trial described above, the remaining information was mainly derived from laboratory studies and no included articles considered cognitive impairment after stroke.^139^

#### **PICO 15:** In people with post-stroke cognitive impairments, does **cognitive rehabilitation (cognitive skill training or compensation strategies)** compared to no rehabilitation, delay cognitive decline or progression to dementia, improve behavioural and psychological symptoms, improve performance in activities of daily living or decrease caregiver burden?

##### Analysis of the current evidence

For the purpose of the present guidelines, we define cognitive rehabilitation as an individualised, structured set of therapeutic activities designed to restore domain-specific cognitive impairments (e.g. attention, visuospatial processing, memory and executive functions) or global cognitive impairment, or overcome these cognitive impairments by means of compensation (e.g. adaptive strategies and assistive devices).^140^ Generally, cognitive rehabilitation includes a combination of restorative and compensatory approaches. The ultimate goal of cognitive rehabilitation is minimising the impact of cognitive impairments on personally relevant aspects of everyday functioning for both the affected individuals and their families.

Given the potential variation in the activities that could be considered as relevant to the cognitive rehabilitation rubric, we pre-specified a list of non-pharmacological interventions that could be considered in the management of post-stroke cognitive impairments but were not considered in this cognitive rehabilitation review. This is an approach that has been used in previous systematic reviews of cognitive rehabilitation.^141^ We considered that interventions exclusively targeting communication, reading, writing and calculation disorders fall outside the scope of the present guidelines, and they are not considered here. Furthermore, we decided to exclude disease self-management/coping interventions, cognitive-motor dual task training, physical training, community reintegration, vocational rehabilitation, patient and caregiver education, neurosensory stimulation (i.e. Snoezelen therapy), nutritional supplements, music-based therapy/instrument playing, art therapy, mindfulness-based interventions, yoga, qigong, acupuncture, non-invasive brain stimulation and cognitive behavioural therapy delivered in isolation or as part of multimodal interventions. We acknowledge that some or all of these interventions might – directly or indirectly – benefit cognitive functioning and therefore could be considered in future versions of these guidelines.

For this PICO, we pre-specified that we would only include randomised controlled trials (RCTs) as observational data in the field are prone to many biases. We also pre-specified that trials would require a minimum of 50 stroke survivors per arm, because we felt as a writing group that smaller studies should be considered proof of concept and their inclusion would make recommendation more prone to publication bias.

We identified a substantial number of controlled clinical trials on cognitive rehabilitation. However, only one trial fulfilled our eligibility criteria. Donkervoort et al.^142^ investigated the efficacy of strategy training for improving functioning in activities of daily living (ADL and primary outcome) and reducing cognitive impairment following left hemisphere stroke with apraxia. One hundred and 13 subacute stroke survivors (mean time since stroke: 100 days) were randomised to an intervention group (*n* = 56) receiving 15 h (SD: 7.7 hours) of strategy training integrated into usual occupational therapy, and a control group (*n* = 57) receiving 19 hours (SD: 15.0 hours) usual occupational therapy alone over an 8-week period. The intervention used compensatory strategies that can be internal (e.g. self-verbalisation) or external (e.g. using pictures of the correct task sequence). Outcomes included observation in four tasks undertaken at baseline, after the 8-week intervention period and at 5 months after baseline. The trial had several methodological limitations including, selected sample, ceiling effect of the ADL observations and 25% drop-out in each trial arm. Strategy training did not influence the apraxic impairment. Regarding ADL functioning, the trial suggested a potential improvement of 0.13 (90%CI: 0.00–0.25) in favour of strategy training post-intervention, corresponding to a small-to-medium effect size. This beneficial effect was not maintained at follow-up (Table 10).

**Table 10.** Summary of findings for PICO 15. Assessment of cognitive rehabilitation for post-stroke dementia.

Population: Left hemisphere stroke patients with apraxia. Mean time since stroke at baseline: 100 days Intervention: Strategy training integrated into usual occupational therapy Comparator: Usual occupational therapy alone				
Outcome	No of trials (No. of participants)	Difference in mean change from baseline to post-intervention	Difference in mean change from baseline to follow-up	Quality of evidence (GRADE)
Apraxia (Apraxia test)	1 (113)	2.00; (90% CI: –1.54, 5.53)	2.24; (90% CI: –2.45, 6.92)	Very low^a^
ADL functioning (ADL observations)	1 (113)	0.13; (90% CI: 0.00, 0.25) effect size = 0.37 (small-to-medium)	−0.01; (90% CI: –0.17, 0.16)	Very low^a^
BPSD	No data			
Caregiver strain	No data			

^a^Downgraded due to imprecision, inconsistency and publication bias.

##### Additional information

Currently, there is an urgent need for methodologically robust trials to support recommendations for clinical practice in cognitive rehabilitation. Despite an increased focus on the importance of cognitive rehabilitation in recent decades, the evidence base is generally characterised by trials with limited methodological quality, for example, inadequate sample size to detect clinically important intervention effects, study designs without control groups and lacking consensus on optimal outcome measures.^143,144^

There is an emerging evidence for beneficial effect of cognitive rehabilitation based on re-learning of compensatory strategies, particularly in the context of meaningful functional tasks for the individual. Although it is established that learning processes require long-term and intensive efforts, existing trials have provided only short periods of cognitive therapy, possibly delivered at insufficient dose to produce a meaningful benefit. Furthermore, trials often lack long-term follow-up and fail to demonstrate evidence of long-lasting intervention effect and transfer effects to untrained cognitive domains and/or functional tasks. Evaluation of strategies to maintain (e.g. booster sessions) and transfer effects is consequently warranted. Little is known about the spontaneous recovery of cognitive impairments over time, this representing a considerable challenge when assessing the true effect of the interventions.

Our choice of outcomes followed the standardised GRADE process, and we reached consensus on the critical outcomes. Choice of outcomes was, in part, to maintain consistency with the other PICO questions in this guideline. Many of the studies returned on our literature search were designed to understand if the intervention improves everyday cognitive function. This is clearly an important outcome and should be considered in future iterations of this guideline.

A final issue we encountered when reviewing the literature is that most trials include populations with mixed diagnoses of stroke and traumatic brain injury. Currently, there is insufficient knowledge on how people recover with similar cognitive impairments, but different aetiology, and so we made the decision to exclude trials with mixed populations from the present guidelines. We appreciate that there is debate on this issue, some argue that given the difficulty in recruiting to cognitive rehabilitation trials future trials may need to be pragmatic and include various brain injuries and adjust for age, psychological and medical comorbidity; while others argue that we should strive for purity in case-mix.

### Prognosis

#### **PICO 16.** In people with a history of stroke, do **multi-item prognostic tools** performed soon after stroke, predict future cognitive decline or dementia?

##### Analysis of the current evidence

In this PICO, we consider multi-item prognostic or prediction tools, that is, assessments that apply scores to a combination of demographic, clinical, radiological or other data to determine the likelihood of a potential outcome, in this case cognitive decline or dementia. We focussed our attention on tools applied in the acute stroke period (first days to weeks). Prognostic tools have been developed and validated for many aspects of stroke care,^145^ for example risk of stroke in a person with atrial fibrillation is assessed using the CHADSVaSC tool,^146^ risk of poor outcome can be assessed with the ASTRAL and other tools.^147^ A similar tool for predicting cognitive outcomes could be useful for ongoing management and discussions with patients and families. However, if such tools are inaccurate in their predictions this could lead to inappropriate treatment decisions or erroneous and potentially harmful discussions regarding future health with the patient and family.

The methods underpinning prognosis evidence synthesis differ in some regards from the standard analysis of trial data. In particular, the application of GRADE to prognosis tools is not as well developed as it is for synthesis of intervention studies. In our GRADE assessment we used the approach of Cochrane prognostic reviews^148^ and considered risk of bias and applicability using the Prediction model Risk Of Bias Assessment Tool (PROBAST) tool,^149^ we considered internal consistency through visual inspection of study level estimates and considered the precision of the summary estimate. More detailed descriptions and examples of prognosis evidence synthesis and reporting are available from Cochrane and others. While we refer to these questions using the PICO terminology, our questions are considering prognostic utility rather than comparative efficacy of interventions, so in formulating these questions our concepts of interest were the population, prognostic factor, outcome and timing of outcome.

Our literature review was based on a recent systematic review^150^ and found seven prognostic tools^151-157^ that had been applied in an acute stroke population and were designed to predict a variety of future cognitive outcomes. Eligible studies were from Europe and Asia and included a variety of stroke types. Five studies assessed for cognitive decline (change in a cognitive score) and two studies assessed for a future diagnosis of dementia (clinical diagnosis). Studies were generally of modest size (range 92 to 283 participants). Variables included in the prognostic tools were items relating to demographics (age and education); stroke severity (NIHSS and GCS); imaging features (atrophy and white matter disease) and scores on cognitive screening tests performed in the acute period.

We assessed methodological quality of the included studies using the PROBAST tool and judged all the included studies at risk of bias (Supplementary Materials). Common limitations of the studies were issues of sample size, handling missing data and lack of external validation. Our intention was to limit our recommendations to those studies that assessed for cognitive outcomes later than 1 year after index stroke. However, none of the included studies had this length of follow-up and most assessed outcomes at three–six months. We included this shorter follow-up for our PICO recommendation but recognise that post-stroke cognition is dynamic and may still be evolving at three and even 6 months post-event. Most included studies presented prognostic utility as an area under an ROC curve. There was a range of scores and most studies had values that would be considered reasonable. However, given the low-quality of evidence for the tools, we could not recommend one over another (Table 11).

**Table 11.** Summary of findings for PICO 16. Assessment of the prognostic utility of multi-item prediction tools for the future diagnosis of post-stroke cognitive impairment or dementia.

Participants: Patients with acute stroke Settings: Acute stroke settings				
Outcome	Effect (AUROC)	No of participants/outcomes	Risk of bias	Quality of evidence (GRADE)
Dementia	No AUROC data available	Two studies 558 participants 216 outcomes	High	Very low^a^
Cognitive decline	AUROC range (0.75 to 0.91)	Five studies 853 participants 379 outcomes	High	Very low^a^

^a^Downgraded due to risk of bias; imprecision; publication bias and inconsistency.

##### Additional information

In addition to the studies looking at post-stroke cognitive change, we also found four articles describing prediction tools for post-stroke delirium.^158-161^ These tools considered similar factors to the tools looking at cognitive decline and dementia. Common factors included demographics (age), stroke severity (NIHSS), stroke type (ischaemia or haemorrhage) and laboratory results (inflammatory markers). Similar to the tools for predicting future cognitive outcomes, and indeed similar to much of the stroke prognosis literature,^162^ the delirium prediction tools had methodological issues around sample size, missing data and lack of external validation.

Many prediction tools have been developed for all-cause or Alzheimer’s dementia. A recent review identified over 70 such tools.^163^ Here again most of the studies have methodological limitations that preclude recommending one tool over any of the others. However, the authors noted that design, conduct and interpretation of studies looking at dementia prediction tools were improving over time.

Studies to date have considered the prognostic accuracy of multi-item prediction tools. We found no trials that described whether the use of a cognitive outcomes prediction tool improved outcomes or changed care pathways.

#### **PICO 17:** In people with a history of stroke, **do structural features on acute brain CT imaging**, predict (at least 1 year from index stroke event) future cognitive decline or dementia?

##### Analysis of the current evidence

In this PICO question, we describe the accuracy of neuroimaging features seen on CT brain scans performed as part of acute stroke care. Although, increasingly sophisticated approaches to brain imaging are available, CT brain remains the most used imaging modality in International acute stroke care and so we felt that an assessment of cognitive prognosis was warranted. The synthesis of prognosis data is different to that of the standard intervention review. A discussion of the methods that underpin our approach is provided in PICO 16. In this analysis, we are describing prognosis in relation to a single prognostic factor (CT imaging finding), rather than a collection of different factors. Thus, for quality assessment, we used the QUIPS tool (quality in prognostic factor studies).^164^

Our literature review found 13 studies examining associations between CT-brain imaging variables and post-stroke dementia or post-stroke cognitive impairment (PSCI) ascertained at least 12 months after stroke (Supplementary Materials).^165-177^ Six studies reported on post-stroke dementia^167,168,170,172,174,175^ and six reported PSCI, one study reported both.^176^ All seven dementia studies excluded patients with prior dementia/cognitive impairment and three excluded patients with prior stroke. Five of seven PSCI studies excluded prior dementia/cognitive impairment and three excluded prior stroke. Reported associations were therefore largely with new post-stroke dementia/PSCI rather than pre-existing dementia. Studies were generally of modest size (range 47 to 445 participants). CT variables examined included atrophy (presence and or severity of generalised atrophy, medial temporal lobe atrophy), white matter hyperintensity (leukoaraiosis) (WMH, presence and/or severity), silent brain infarcts (SBI) and acute stroke lesion characteristics although not all features were reported in every study. There was considerable heterogeneity in the way variables were measured.

We assessed methodological quality using the QUIPS tool^164^ and judged all the included studies at risk of bias (Supplementary Materials). Common limitations were small sample sizes, attrition and handling of missing data. In addition, few studies adjusted associations for important covariates.

Given the small number of studies per imaging variable and the heterogeneity between studies, we did not create summary estimates, full details of the included articles and their study level results are in Supplementary Materials. Two studies reported on presence versus absence of atrophy and dementia. One showed an association with dementia (OR = 5.86, 95% CI = 1.73–19.87)^175^; the other suggested a possible association, but with substantial uncertainty in the estimate (OR = 7.7, 95% CI = 0.9–65.2)^176^. Three studies examined atrophy and PSCI, of which only one reported a positive association with PSCI (*p* < 0.001, no size of effect),^166^ one approached a positive association (OR = 2.2, 95% CI: 0.9–5.1)^176^ and one found no association.^177^ Three studies examined severity of atrophy^170,172,175,^ only one of which reported significant associations between post-stroke dementia and severe generalised atrophy (RR = 2.19, 95% CI = 1.5–3.17) and between post-stroke dementia and medial temporal lobe atrophy (RR = 2.3, 95% CI: 1.1–4.7).^170^

All studies examining presence versus absence of WMH reported positive associations with dementia^174-176^ e.g. OR = 3.9, 95% CI = 1.2–12.0 (unadjusted),^175^ but relationships between WMH and PSCI were less certain. Severity of WMH was associated with dementia in three^167,175,170^ of five studies^172,177^ (e.g. RR = 2.09, 95% CI: 1.05–4.13). Two^170,176^ of three studies^168^ found associations between SBI and post-stroke dementia: OR = 5.6, 95% CI: 1.4–22.5 and RR = 2.09, 95% CI: 1.05–4.13. Acute stroke features were too heterogeneous to draw conclusions regarding their associations with post-stroke cognitive outcomes (Table 12).

**Table 12.** Summary of findings for PICO 17. Assessment of the prognostic utility of lesions on acute CT brain imaging for predicting future diagnosis of post-stroke cognitive impairment or dementia.

Participants: Patients with acute stroke Prognostic factor: Acute stroke CT brain imaging Timing of follow-up: At least 12 months from index stroke Settings: Acute stroke settings				
Outcome	CT finding	No of participants/outcomes	Risk of bias	Quality of evidence (GRADE)
Dementia	Atrophy	Two studies 558 participants 216 outcomes	High	Low^a^
PSCI		Five studies 853 participants 379 outcomes	High	Very low^a^
Dementia	White matter hyperintensity	Two studies 558 participants 216 outcomes	High	Low^a^
PSCI		Five studies 853 participants 379 outcomes	High	Very low^a^
Dementia	Silent brain infarction	Two studies 558 participants 216 outcomes	High	Very low^a^
PSCI		Five studies 853 participants 379 outcomes	High	Very low^a^

^a^Downgraded due to risk of bias; imprecision.

^b^Downgraded due to risk of bias; imprecision; publication bias and inconsistency.

##### Additional information

There are many studies of CT-brain imaging in relation to all-cause dementia and specifically for Alzheimer’s dementia.^178^ These studies show associations between WMH and cognitive function (and also gait and balance and functional disability) including prediction of cognitive decline and dementia. Similar associations have been demonstrated between generalised cerebral atrophy^179^ and temporal lobe atrophy^180^ and Alzheimer’s dementia.

It would seem intuitive that the presence of findings such as atrophy and WMH on CT brain imaging performed for acute stroke would indicate a prevalent neurodegenerative process and so would be associated with future cognitive outcomes. However, in our PICO analysis described above we found only a limited published literature. Thus, the prognostic utility of these CT imaging biomarkers, in particular their utility over and above the basic clinical and demographic factors already known to be associated with future dementia, remains to be described with adequate certainty and precision.

The clinical–radiological correlations described in the stroke and general dementia themed articles are not perfect. In older adults in particular, the relationship between neuroimaging features and the clinical phenotype can be weak.^181^ It seems possible that single factors alone may never be sufficiently predictive to alter clinical pathways.

In this review, we have considered only the prognostic properties of the imaging features. A more complex but more clinically relevant question is whether knowledge of the likely cognitive prognosis makes a difference to patient outcomes. With no proven acute interventions to arrest or delay potential post-stroke cognitive consequences, it could be argued there is no value in acute prognostication. To study this question would require a different study paradigm where patients or centres are randomised to using a prediction tool and patient pathways and outcomes are described. We found no studies that used this approach.

#### **PICO 18** In people with a history of stroke, **do structural features on acute brain MR imaging**, predict (at least 1 year from index stroke event) future cognitive decline or dementia?

##### Analysis of the current evidence

In this PICO question, we describe the accuracy of neuroimaging features seen on standard MRI brain scans performed as part of acute stroke care. The synthesis of prognosis data is different to that of the standard intervention review. A discussion of the methods that underpin our approach is provided in PICO 16 and 17. Brain imaging is invariably performed in acute stroke for diagnostic purposes and to guide treatment decisions. Although CT is standard practice in acute stroke, MRI is used frequently, especially in regional centres in the developed world, so a better understanding of the prognostic value of routinely acquired MR-brain imaging findings for future cognitive prognosis is required.

We found 10 relevant studies of consecutive stroke patients examining associations between MR-brain imaging variables and cognition. Nine^182-190^ described PSCI outcomes and were included in our GRADE table assessment, a single study described post-stroke dementia defined using NIA-AA criteria^175^ (Supplementary Materials). Studies used a variety of methods to define PSCI (multidomain cognitive screening tools and differing neuropsychological batteries). Two studies did not exclude patients with prior dementia/cognitive impairment^182,187^ and five excluded patients with prior stroke.^182,185,187,188,190^ Reported associations were therefore largely but not exclusively with new post-stroke PSCI rather than pre-existing dementia/PSCI. Studies were generally of small or modest size (range 55 to 451 participants). MR variables examined included white matter hyperintensities of presumed vascular origin (WMH), global atrophy, stroke lesion volume, cerebral microbleeds, perivascular spaces, stroke lesion related factors including stroke location and an aggregate small vessel disease score (combining different features of SVD). Not all features were reported in every study. There was considerable heterogeneity in the way variables were measured.

Common study limitations were small sample sizes, attrition, handling of missing data, lack of standardisation of measures and adjustment for important covariates. In addition, outcome measures for PSCI were heterogeneous and the predominant use of cognitive screening tools may have missed subtle yet important changes.

Given the small number of studies per imaging variable and the heterogeneity between studies, we did not create summary estimates, Full details of the included articles and their study level results are in Supplementary Materials.

The article that reported post-stroke dementia outcomes^175^ included 218 participants and described positive association with WMH (Fazekas score), HR = 1.80, 95% CI: 1.17–2.75 (*p* = 0.007, adjusted for age) and positive association with cortical atrophy score, HR = 2.02, 95% CI: 1.28–3.19 (*p* = 0.002, adjusted for age).

For PSCI, most evidence was available for WMH although again there was heterogeneity in measurement method as well as outcome assessment. Overall, six^184-187^ of eight studies examining WMH reported positive association with PSCI, and this was robust to adjustment at least for demographic factors (e.g. OR = 1.58 (95% CI: 1.15–2.44), adjusted, total Fazekas score; OR = 1.52 (95% CI: 1.01–2.29), Fazekas 0–3, unadjusted). Only two studies examined atrophy (global),^186,189^ one of which showed associations in unadjusted but not adjusted analyses.^189^ Lesion volume findings were conflicting with associations reported with a number of cognitive domains including spatial memory, recall but not global cognitive impairment by the MMSE. Acute stroke features were variably examined and too heterogeneous to draw conclusions.

Many of the articles described various small vessel disease features including cerebral microbleeds^183-187^ and perivascular spaces.^185-187^ Findings for cerebral microbleeds were conflicting and no associations were seen with perivascular spaces. Three studies examined a global small vessel disease score combining different imaging features of small vessel disease.^185-187^ Two^185,186^ of three found associations in adjusted analyses, and the use of combination measures is promising but at present there are too few data to draw conclusions about their clinical utility in this context (Table 13).

**Table 13.** Summary of findings for PICO 18. Assessment of the prognostic utility of lesions on acute MR-brain imaging for predicting future diagnosis of post-stroke cognitive impairment or dementia.

Participants: Patients with acute stroke Prognostic factor: Acute stroke MR-brain imaging Timing of follow-up: At least 12 months from index stroke Settings: Acute stroke settings				
Outcome	MRI abnormality	No of participants	Risk of bias	Quality of evidence (GRADE)
PSCI	White matter hyperintensity	Eight studies 1781 participants	High	Moderate
PSCI	Atrophy	Two studies 415 participants	High	Very low^a^
PSCI	Lesion volume	Four studies 895 participants	High	Low^b^
PSCI	Small vessel disease score	Three studies 925 participants	High	Low^b^
PSCI	Cerebral microbleeds	Four studies 980 participants	High	Very low^a^
PSCI	Perivascular spaces	Three studies 925 participants	High	Very low^a^

^a^Downgraded due to risk of bias; imprecision; publication bias and inconsistency.

^b^Downgraded due to risk of bias and imprecision.

PSCI: post-stroke cognitive impairment.

##### Additional information

There are many studies of MR-brain imaging in relation to all-cause dementia and specifically for Alzheimer’s dementia. These studies show associations between WMH and cognitive function (and also gait and balance and functional disability) including prediction of cognitive decline and dementia.^191^ Similar associations have been demonstrated between generalised cerebral atrophy and all-cause dementia^192^ and between temporal lobe atrophy and Alzheimer’s dementia,^193^ although specificity for Alzheimer’s disease is not 100%.

The predictive value of baseline brain imaging findings for dementia at more than 1 year post-stroke has also been examined in large cohorts in which brain imaging variables were obtained using either CT or MRI (*n* = 919, Mok et al. and *n* = 2305 Pendlebury et al.).^194,195^ Both these studies, which excluded pre-stroke dementia, showed strong associations with WMH (MRI) and leukoaraiosis (CT) and late post-stroke dementia (OR = 1·49 (95% CI: 1·22–1·82) adjusted for age, sex, education and stroke severity, Pendlebury et al.) and presence of ≥3 lacunes and confluent WMH (OR = 2.6 (95% CI: 1.3–4.9) adjusted age, sex and education, Mok et al.).

We also reviewed the evidence for MR-brain imaging features based on non-structural MRI modalities to predict the cognitive outcomes after stroke: the most commonly used modalities were Diffusion Tensor Imaging (DTI), Diffusion Weighted Imaging (DWI) and functional MRI. The evidence was inconclusive as most studies used small sample sizes (*n* = 1–148), combined with a maximal follow-up of 6 months, or focused exclusively on aphasia, which is less relevant to our PICO.

## Discussion

Despite the importance of post-stroke cognitive impairment and dementia, we found a marked paucity of high-quality data from RCTs. In some areas, such as pharmacological secondary prevention, there were some, but limited, data, while in other areas, such as cognitive rehabilitation after stroke, there were no data from definitive multi-centre studies. Finally, for some areas, such as the effectiveness of a policy of cognitive screening, there were no trial data at all. This evidence–practice research gap is seen in many areas of dementia work, but seems especially problematic in the field of post-stroke cognitive impairment.^196^

Many high-quality trials have demonstrated that treating cardiovascular risk factors such as hypertension reduces recurrent stroke risk. In view of the known association between stroke and dementia, one might expect such treatments to also reduce future dementia. Lifestyle interventions, medical risk factor modification and cognitive stimulation have all been mentioned as potential preventive strategies after stroke. Our review of the literature suggests that there is no convincing evidence that any of these interventions can prevent cognitive decline or dementia. A similar situation was found for antithrombotic therapy. Of note, recent observational data from large population datasets has suggested that treatment of atrial fibrillation with anticoagulation markedly reduced dementia risk, but these results need confirmed in a prospective randomised trial.^197,198^

How intensively cardiovascular risk factors should be treated, particularly blood pressure, has also been debated. Again, there were limited high-quality data from post-stroke dementia to address this question. However, for a non-stroke cohort, the recent SPRINT-MIND study suggested intensive blood pressure lowering to a systolic of 120 mmHg, compared with standard lowering to 140 mmHg, was associated with a reduced incidence of mild cognitive decline and the combined endpoint of MCI and dementia.^199^ There has been concern that intensive blood pressure lowering may have risks in people living with extensive small vessel disease and impaired cerebral autoregulation, but the recent PRESERVE study showed no reduction in cerebral blood flow, increased white matter damage or difference on cognition associated with blood pressure lowering to 125 mmHg compared with 140 mmHg.^200^ Consistent with this finding, the SPS3 cognitive sub-study reported no adverse consequences of lowering blood pressure to this level.^23^

Cognitive performance after stroke differs greatly and identifying participants at increased risk may increase the potential effect of a preventive intervention. Currently, there are no validated instruments to reliably identify those at highest risk of developing post-stroke cognitive impairment, although single characteristics including stroke severity, low education and age are associated with a higher risk. Whether high risk individuals can benefit more from interventions aiming to prevent cognitive decline and dementia should be focus of future research. A limitation of preventive strategies in patients with a history of stroke, especially lifestyle interventions, is the high drop-out rate. Improving adherence to these interventions, may contribute to better cognitive outcomes. After stroke, barriers for participating in rehabilitation and in health programmes, such as social isolation, depression and inactivity, are frequently seen. Moreover, these are all risk factors for developing (post-stroke) cognitive decline and dementia.

The evidence around prevention of post-stroke cognitive decline remains imperfect, and unfortunately, the same was true for trials of interventional treatments including cognitive training and medications such as cholinesterase inhibitors. We found few RCTs which investigated cognitive interventions after stroke, included more than 50 participants per group and assessed clinical outcomes over a period of longer than 6 months. We noted an increased amount of research within this area, generating emerging evidence that cognitive rehabilitation, in particular compensatory strategies in the context of individually relevant functional tasks, may be beneficial for people with post-stroke cognitive impairments. However, this evidence has relied primarily on trials with methodological limitations such as inadequate sample size to detect clinically important intervention effects, study designs without control groups, lack of consensus on optimal outcome measures, insufficient treatment dose and lack of long-term follow-up. There is an urgent need for methodologically robust trials on cognitive rehabilitation.

Similarly, we found no robust data that pharmacological interventions including cholinesterase inhibitors and memantine improved symptoms or delayed progression to dementia. There has been debate as to whether effects reported with cholinesterase inhibitors in vascular dementia trials are due to a true effect on vascular dementia, or an effect on concurrent Alzheimer’s pathology. Mixed pathology becomes increasingly common with increasing age. To address this question, a randomised controlled trial examined donepezil in a model of pure vascular dementia, CADASIL. Although there was a significant effect on the secondary endpoint of executive dysfunction, there was no improvement in the primary cognitive endpoint or activities of daily living.^126^ Therefore we concluded that, in predominantly vascular cognitive impairment, the effect of these drugs is minimal. However, older adults with stroke who have other co-existent neurodegenerative diseases responsive to cholinesterase inhibitors may benefit from a trial of these drugs. Our conclusions with memantine were similar. In contrast, although there was again limited data, we could find no evidence for the use of actovegin and cerebrolysin following stroke and noted concerns around safety and cost.

The first step to effective management of post-stroke cognitive impairment is identification of the problem. While some recommend cognitive screening of all suspected stroke admissions in the acute stroke setting, we found no robust evidence to support this approach. We were able to give estimates of the accuracy of various cognitive screening tools, but there were less data for newer tools such as the Oxford Cognitive Screen. Variation in the choice of cognitive assessment is apparent in stroke research and practice. Our data did not suggest a single ‘best’ screening tool for post-stroke cognition, and there were few studies that compared differing test strategies. Articles focussed on accuracy metrics, but the choice of tool should also be based on aspects such as feasability, availability of training and cost.

We evaluated whether multi-item prognostic tools, as well as structural features on CT and/or MRI imaging, obtained in the acute stroke period (days to weeks) were able to contribute to the prediction of dementia and PSCI after 12 months. Multi-item prognostic tools combined variables such as patient demographics, stroke severity, neuropsychological scores and imaging data. We concluded that there is currently a lack of evidence to support the clinical implementation of such tools. Although there is evidence that white matter hyperintensities on both CT and MRI may predict dementia risk, there is insufficient evidence for the routine use of CT or MRI parameters to inform prognosis decision making. This is an area which requires further work. A recent study in 2950 stroke patients found that infarcts in the left frontotemporal lobes, left thalamus, and right parietal lobe were strongly associated with PSCI, and suggested that quantitative mapping of the stroke lesion may provide useful prognostic information.^201^ Overall, we encountered numerous issues of sample size, attrition bias, adjustment for covariates and a lack of external validation, which need to be addressed in future studies. In particular, it should be noted that quantification of the severity and location of structural brain imaging abnormalities including atrophy and WMH require the application of visual rating scales by trained observers or at least the application of semi-automated software programmes. This limits the clinical utility of imaging variables for dementia prediction in routine clinical practice and highlights the need to determine their independent predictive value over and above other, more easily acquired clinical factors. An additional consideration is how useful prognostic screening for dementia is, in the absence of a specific preventative treatment. However, we concluded that it is important to develop robust methods of identifying future dementia risk so that when treatments are available those likely to benefit can be identified.

Post-stroke cognitive impairment has been consistently identified as a major area of concern for stroke survivors and their families, and a high priority area for future research. Despite this, our comprehensive review identified a paucity of high-quality data informing optimal management in this area. Many studies have been small, single centre and with inadequate control arms. In all areas large adequately powered randomised controlled trials with robust endpoints are required. These need to be multi-centre to increase generalisability. We would strongly encourage cognitive endpoints to be added to ongoing secondary prevention trials, adopting a model similar to the addition of cognitive endpoints to the SPRINT-MIND sub-study of the SPRINT RCT.^199^

Although cognitive issues have not featured as prominantly in stroke Guidelines as may be expected based on their prevalance and importance, there have been some recent publications relevant to the field. The White Paper on cognitive impairment and cerebrovascular disease from ESO^202^ complements the content of this Guideline. The White Paper emphasis the need to consider cognitive effects in all people living with stroke and highlights the importance of vascular secondary prevention. The Canadian Stroke Best Practice Recommendations (CSBPR) for mood, cognition and fatigue^48^ has a broader remit than our Guideline but covers many similar topics. The CSBPR have more detailed recommendations on many aspects of cognitive rehabilitation and offer guidance on specific rehabilitation strategies. The Australian Stroke Foundation have a ‘living’ Guideline (https://informme.org.au/Guidelines) that updates in response to new evidence. This Guideline is not specific to cognition but has sections on assessment and management of cognitive issues across domains of perception, attention, memory, executive function, apraxia and neglect.

Completing large, multi-centre trials in the field of post-stroke cognition is difficult. The lack of evidence to make strong guideline recommendations should not be construed as lack of enthusiasm or lack of will to tackle this problem. We found many examples of pilot or phase II trials with data that were promising but did not meet our pre-specified criteria for inclusion. We have offered suggestions to trialists around design and conduct of trials, but we also make an appeal to research funders to support definitive phase III trials. For clinicians, although we can offer few strong recommendations, we hope our Expert Consensus Statements are helpful. It would be wrong to take a nihilistic view and use the lack of evidence-based recommendations in this Guideline as a tool to reduce or remove clinical and research activity in the post-stroke cognition space. Quite the opposite, we would hope that this guideline acts as a catalyst to support future research and service development.

### Priorities for future research

Based on their review of the evidence for the PICO questions, and drawing on their own experience and knowledge of the research landscape, each of the writing groups suggested priorities for future research in the field of post-stroke cognitive impairment.

### Prevention

1. Investigate who is at the highest risk of post-stroke dementia using widely available clinical parameters, including availability in low- and middle-income countries
2. Determine barriers and facilitators to adherence to preventive interventions including lifestyle and medication
3. Include long-term outcomes related to cognitive impairment and dementia in secondary prevention trials in stroke

### Diagnosis

4. Assess the efficacy (impact on outcomes important to stroke survivors), costs and harms of routine cognitive screening of all hospital admissions with suspected stroke.
5. Determine the comparative utility of cognitive screening tools for use in stroke, including assessment of feasibility, burden and associated costs.
6. Determine the optimal methods for conducting remote assessments of cognition.

### Treatment

7. Robust randomised controlled trials of de-prescribing, nootropics and cognitive rehabilitation strategies, with longer term outcomes and consideration of safety and cost benefit.
8. Research should consider the similarities and differences between treatments for post-stroke dementia and treatments for other dementia subtypes or other brain injuries.

### Prognosis

9. Validate any potential prognostic tool in independent cohorts with suitable sample size and consideration of additional prognostic benefit beyond standard assessments.
10. Evaluate the effect of the implementation of prediction tools on clinical outcomes.

## Plain Language Summary

Problems with memory and thinking are common following stroke. Thankfully, for many stroke survivors these problems improve over time, but for some people the problems persist and can have a major effect on independence and quality of life. When memory and thinking problems are severe, we may use the term post-stroke dementia.

There are lots of potential interventions for the memory and thinking problems that can follow stroke. Across Europe healthcare professionals use differing approaches to treatment with little consensus on the optimal strategy. In this situation, a guideline that makes recommendations on best practice can be useful.

In this guideline, we collected relevant scientific studies that looked at post-stroke memory and thinking. We divided the guideline into four sections: prevention, diagnosis, treatment and prediction (prognosis). Each section was written by a team of experts who reviewed all the available research. Where possible we combined the results of studies and compared different treatments. If the published studies couldn’t give a definitive answer, we used the knowledge and experience of our expert writing group of healthcare professionals and researchers to offer practical guidance.

For the **prevention** section, we found very few studies that described the effects of medications or lifestyle on memory and thinking following a stroke. Actions such as taking medications for high blood pressure and getting more exercise seem to have lots of health benefits and are generally recommended. However, we don’t know if these actions also prevent dementia and other thinking problems following a stroke.

There is no doubt that accurate **diagnosis** of dementia is important where there is a concern regarding memory and thinking. Some stroke services screen every new stroke patient for dementia. We found no studies that have tested this approach. We did find several different pencil and paper tests that can be used for the assessment of memory and thinking problems. Many of these tests have been used in stroke survivors. Looking at the accuracy of the tests, there was no clearly superior option. In choosing an assessment for a stroke survivor, it is important to consider the whole person – can they hold a pen and, do they have the energy to complete a long test. With COVID-19 restrictions, many services have started using telephone or video call assessments. Despite the increasing use of these technologies, we found very few studies on the topic.

We looked at **treatment** of post-stroke dementia using those medications that are often prescribed to people with Alzheimer’s dementia – cholinesterase inhibitors and memantine. There were very few studies that assessed these medications in stroke survivors. We concluded that having a stroke should not be a barrier to prescribing these medications to a person with dementia who otherwise would be suitable for treatment. However, we could not make a recommendation around using these medications for all people with post-stroke dementia. In some parts of Europe, animal-derived compounds (nootropics) have been used to help brain recovery following stroke. Again, there were few studies with a specific focus on memory and thinking. Where studies were available, we had concerns around the potential burden, cost and safety of these treatments. A large part of the treatment of memory and thinking issues involves rehabilitation. Although we found many studies looking at methods of rehabilitation, most had too few participants or did not look at longer term effects. So, we are still uncertain as to the best methods of rehabilitation for memory and thinking problems following stroke.

If we could **predict** who would develop important and persisting memory and thinking problems following a stroke, we could target our treatments accordingly. There are lots of individual factors that are associated with risk of dementia following a stroke. We looked at whether combining these factors into a prediction score could identify those people who would develop problems. We found various examples of dementia prediction tools, but no tool was good enough to be used in clinical practice. Finally, we looked at whether brain scans, performed as part of usual stroke care, could help identify people who will develop memory and thinking issues. Results of studies were mixed and often conflicting. One feature seen on MRI brain scans, abnormal signals in the deep structures of the brain, did consistently seem to be associated with future risk of dementia and related issues. However, it is not clear if using this MRI feature improves prediction over and above standard clinical judgement.

Although we reviewed a lot of scientific studies, for many of the questions in our guideline we concluded that there simply isn’t enough information to give a definitive answer. This is frustrating for researchers and clinicians, but it also allows us to select priority areas to target future research studies. We would hope that updated versions of this guideline can properly address these important aspects of stroke care.

## Supplemental Material

sj-pdf-1-eso-10.1177_23969873211042192 – Supplemental Material for European Stroke Organisation and European Academy of Neurology joint guidelines on post-stroke cognitive impairment Click here for additional data file. Supplemental Material, sj-pdf-1-eso-10.1177_23969873211042192 for European Stroke Organisation and European Academy of Neurology joint guidelines on post-stroke cognitive impairment by Terence J Quinn, Edo Richard, Yvonne Teuschl, Thomas Gattringer, Melanie Hafdi, John T O’Brien, Niamh Merriman, Celine Gillebert, Hanne Huyglier, Ana Verdelho, Reinhold Schmidt, Emma Ghaziani, Hysse Forchammer, Sarah T Pendlebury, Rose Bruffaerts, Milija Mijajlovic, Bogna A Drozdowska, Emily Ball and Hugh S Markus in European Stroke Journal

sj-pdf-2-eso-10.1177_23969873211042192 – Supplemental Material for European Stroke Organisation and European Academy of Neurology joint guidelines on post-stroke cognitive impairment Click here for additional data file. Supplemental Material, sj-pdf-2-eso-10.1177_23969873211042192 for European Stroke Organisation and European Academy of Neurology joint guidelines on post-stroke cognitive impairment by Terence J Quinn, Edo Richard, Yvonne Teuschl, Thomas Gattringer, Melanie Hafdi, John T O’Brien, Niamh Merriman, Celine Gillebert, Hanne Huyglier, Ana Verdelho, Reinhold Schmidt, Emma Ghaziani, Hysse Forchammer, Sarah T Pendlebury, Rose Bruffaerts, Milija Mijajlovic, Bogna A Drozdowska, Emily Ball and Hugh S Markus in European Stroke Journal

sj-pdf-3-eso-10.1177_23969873211042192 – Supplemental Material for European Stroke Organisation and European Academy of Neurology joint guidelines on post-stroke cognitive impairment Click here for additional data file. Supplemental Material, sj-pdf-3-eso-10.1177_23969873211042192 for European Stroke Organisation and European Academy of Neurology joint guidelines on post-stroke cognitive impairment by Terence J Quinn, Edo Richard, Yvonne Teuschl, Thomas Gattringer, Melanie Hafdi, John T O’Brien, Niamh Merriman, Celine Gillebert, Hanne Huyglier, Ana Verdelho, Reinhold Schmidt, Emma Ghaziani, Hysse Forchammer, Sarah T Pendlebury, Rose Bruffaerts, Milija Mijajlovic, Bogna A Drozdowska, Emily Ball and Hugh S Markus in European Stroke Journal

sj-pdf-4-eso-10.1177_23969873211042192 – Supplemental Material for European Stroke Organisation and European Academy of Neurology joint guidelines on post-stroke cognitive impairment Click here for additional data file. Supplemental Material, sj-pdf-4-eso-10.1177_23969873211042192 for European Stroke Organisation and European Academy of Neurology joint guidelines on post-stroke cognitive impairment by Terence J Quinn, Edo Richard, Yvonne Teuschl, Thomas Gattringer, Melanie Hafdi, John T O’Brien, Niamh Merriman, Celine Gillebert, Hanne Huyglier, Ana Verdelho, Reinhold Schmidt, Emma Ghaziani, Hysse Forchammer, Sarah T Pendlebury, Rose Bruffaerts, Milija Mijajlovic, Bogna A Drozdowska, Emily Ball and Hugh S Markus in European Stroke Journal
