# Supplementary material for: European Stroke Organisation and European Academy of Neurology joint guidelines on post-stroke cognitive impairment
Source: Eur Stroke J. 2021 Oct 8;6(3):I–XXXVIII. doi: 10.1177/23969873211042192 (PMC8564156; doi:10.1177/23969873211042192)
Supplement: sj-pdf-1-eso-10.1177_23969873211042192 – Supplemental Material for European Stroke Organisation and European Academy of Neurology joint guidelines on post-stroke cognitive impairment [file sj-pdf-1-eso-10.1177_23969873211042192.pdf]

Supplementary Material Contents

	Page
**Methods**	3
**Prevention**	
PICO 1 and PICO 3 search syntax	4-5
PICO 1 PRISMA flow diagram	6
PICO 1 Risk of bias assessment	7
PICO 2 and PICO 5 search syntax	8
PICO 2 Risk of bias assessment	9
PICO 2 Summary of findings table	10
PICO 3 PRISMA flow chart	11
PICO 3 Risk of bias assessment	12
PICO 4 search syntax	13
**Diagnosis**	
PICO 6 Search syntax	14-16
PICO 6 PRISMA flow chart	17
PICO 7-9 Search syntax	18-19
PICO 7-10 PRISMA flow diagram	20
PICO 7 Risk of bias assessment	21
PICO 8 Risk of bias assessment	22
PICO 9 Risk of bias assessment	23

PICO 10 Search syntax **24-25**

PICO 10 PRISMA flow diagram **26**

PICO 11 Search syntax **27**

PICO 11 Risk of bias assessment **28**

Treatment

PICO 12-14 search syntax **29**

PICO 12 and PICO 14 risk of bias assessment **30**

PICO 15 search syntax **31-34**

PICO 15 PRISMA flow diagram **35**

PICO 15 risk of bias assessment **36**

Prognosis

PICO 16 search syntax **37-39**

PICO 16 PRISMA flow diagram **40**

PICO 16 Risk of bias assessment **41**

PICO 17 and 18 search syntax **42-45**

PICO 17 and 18 PRISMA flow chart **46**

PICO 17 risk of bias **47**

PICO 18 risk of bias **48**

PICO 17 summary of included studies **49**

PICO 18 summary of included studies **50**

References 51-52

Methods

Methods for the review of literature and synthesis are described in the main body of the text.

Some PICO questions used a single search and then extracted papers relevant to separate PICOS, the search is described only once.

Search syntax given is for Medline (Ovid). All searches used Embase (Ovid), Embase (Ovid), CINAHL (EBSCO) and PsychInfo (EBSCO) databases.

Where a PICO used the search strategy for a relevant contemporary review, the citation for the original review is given.

Risk of bias assessment table were created using the RobVis app.[1]

PICO 1 and 3 search syntax

PICO 1. In people with a history of stroke, do **monitored lifestyle based (exercise, dietary change, alcohol moderation, weight loss, smoking cessation) interventions, alone or in combination** compared to care as usual: prevent future cognitive decline? prevent future dementia?

PICO 3. In people with a history of stroke, do monitored multicomponent interventions (lifestyle and pharmacological), compared to usual care, prevent: future cognitive decline or dementia?

Search strategy modified from the protocol of a Cochrane review

1. Hafdi M, Hoevenaar-Blom MP, Richard E. Multi-domain interventions for the prevention of dementia and cognitive decline. Cochrane Database of Systematic Reviews 2020, Issue 4. Art. No.: CD013572. DOI: 10.1002/14651858.CD013572

1 exp Dementia/
2 Delirium/
3 Wernicke Encephalopathy/
4 Delirium, Dementia, Amnestic, Cognitive Disorders/ or Cognitive Dysfunction/
5 dement*.mp.
6 alzheimer*.mp.
7 (lewy* adj2 bod*).mp.
8 (chronic adj2 cerebrovascular).mp.
9 ("organic brain disease" or "organic brain syndrome").mp.
10 "benign senescent forgetfulness".mp.
11 (cerebr* adj2 deteriorat*).mp.
12 (cerebral* adj2 insuEicient*).mp.
13 ("major neurocognitive disorder*" or "Cognitive Impairment and Disability").ti,ab.
14 or/1-13 1007787
15 multidomain.ti,ab.
16 "multi-domain".ti,ab.
17 "Multi-component".ti,ab.
18 "Multicomponent".ti,ab.
19 exp Combined Modality Therapy/
20 (multi* adj3 domain*).ti,ab.
21 (Multi* adj3 component*).ti,ab.
22 exp Exercise/
23 exp Exercise Therapy/

24 "Interval Train*".ti,ab.
25 "Physical activit*".ti,ab.
26 "Physical train*".ti,ab.
27 "Physical Therap*".ti,ab.
28 Exercis*.ti,ab.
29 "physical fitness".ti,ab.
30 exp Diet/
31 exp Vitamins/
32 exp Minerals/
33 exp Dietary Supplements/
34 Calcium Carbonate/
35 vitamin*.ti,ab.
36 diet*.ti,ab.
37 or/15-36
38 14 and 37
39 exp Secondary Prevention/
40 exp Primary Prevention/
41 "delay onset".ti,ab.
42 ((cognit* or cognition or memory or mental or brain) adj3 (impair* or decline* or deficit* or loss or lose or stop* or reduc*)).ti,ab.
43 prevent*.ti,ab.
44 taper*.ti,ab.
45 avoid*.ti,ab.
46 "cut* down".ti,ab.
47 or/39-46
48 38 and 47
49 cerebrovascular disorders/ or exp basal ganglia cerebrovascular disease/ or exp brain ischemia/ or exp carotid artery diseases/ or exp intracranial arterial diseases/ or exp "intracranial embolism and thrombosis"/ or exp intracranial hemorrhages/ or stroke/ or exp brain infarction/ or exp vertebral artery dissection/ 1413693
50 (stroke or cerebrovasc$ or brain vasc$ or cerebral vasc$ or cva$ or apoplex$).tw.
51 (stroke or cerebrovasc$ or brain vasc$ or cerebral vasc$ or cva$ or apoplex$).tw.
52 or/49-51
53 48 and 52
54 (randomized controlled trial or controlled clinical trial or randomized or placebo or drug therapy or randomly or trial or groups).ti,ab.
55 53 and 54
56 limit 55 to human
57 remove duplicates from 56

PICO 1 PRISMA Flow chart

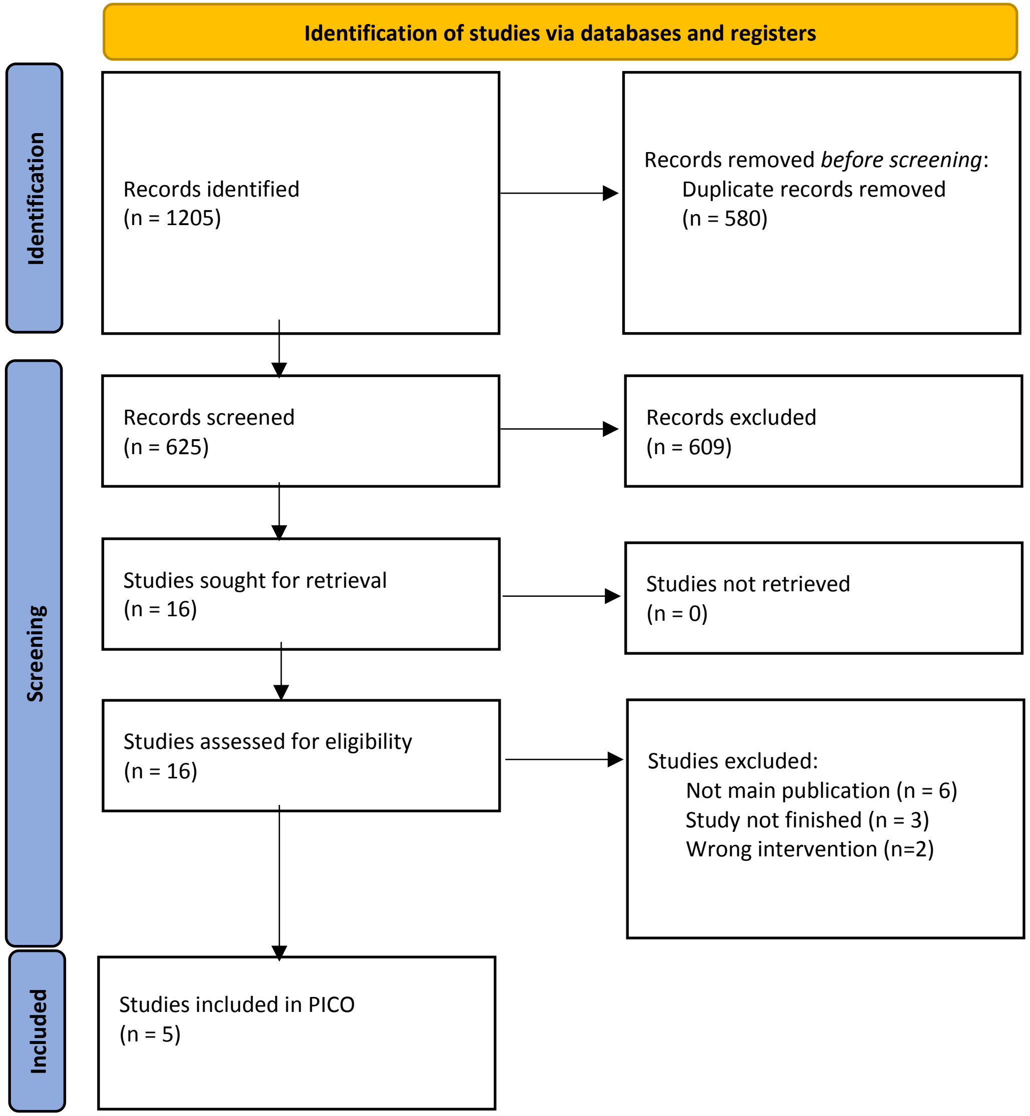

PICO 1 Risk of bias assessment:

	Random sequence generation (selection bias)	Allocation concealment (selection bias)	Blinding of participants and personnel (performance bias)	Blinding of outcome assessment (detection bias)	Incomplete outcome data (attrition bias)	Selective reporting (reporting bias)	Other bias
ASPIS (2015)	+	+	−	+	+	+	+
Cheng et al. (2018)	+	+	?	?	+	?	+
Ihle-Hansen et al. (2014)	+	+	?	+	+	+	+
LAST (2019)	+	+	−	+	+	+	+
MoveIT (2018)	+	−	−	+	+	+	+

PICO 2 and 5 search syntax

PICO2: In people with a history of stroke, does **monitored intensive management of vascular risk factors**, compared to usual care, prevent: future cognitive decline or dementia?

PICO 5: In people with a history of post-stroke dementia does, **stopping pharmacological management of vascular risk factors** (de-prescribing), compared to continuing these medications prevent: future cognitive decline or improve health related quality of life?

As pre-specified, where a high-quality systematic review had been recently published, We used the existing search strategy, but operated the inclusion/exclusion specific to this review question. We then complemented with a focussed search of the recent literature.

For this PICO we searched the included and excluded studies from the following reviews:

1. Hughes D, Judge C, Murphy R, et al. Association of Blood Pressure Lowering With Incident Dementia or Cognitive Impairment: A Systematic Review and Meta-analysis. JAMA. 2020;323(19):1934–1944.
2. Zonneveld TP, Richard E, Vergouwen MDI, Nederkoorn PJ, de Haan RJ, Roos YBWEM, Kruyt ND. Blood pressure‐lowering treatment for preventing recurrent stroke, major vascular events, and dementia in patients with a history of stroke or transient ischaemic attack. Cochrane Database of Systematic Reviews 2018, Issue 7. Art. No.: CD007858.
3. Cholesterol treatment Trialists Collaboration. Efficacy and safety of statin therapy in older people: a meta-analysis of individual participant data from 28 randomised controlled trials. Lancet. 2019; 393:407-415
4. McGuinness B, Craig D, Bullock R, Passmore P. Statins for the prevention of dementia. Cochrane Database of Systematic Reviews 2016, Issue 1. Art. No.: CD003160.
5. Jordan F, Quinn TJ, McGuinness B, Passmore P, Kelly JP, Tudur Smith C, Murphy K, Devane D. Aspirin and other non‐steroidal anti‐inflammatory drugs for the prevention of dementia. Cochrane Database of Systematic Reviews 2020, Issue 4. Art. No.: CD011459.
6. Areosa Sastre A, Vernooij RWM, González-Colaço Harmand M, Martínez G. Effect of the treatment of Type 2 diabetes mellitus on the development of cognitive impairment and dementia. Cochrane Database of Systematic Reviews 2017, Issue 6. Art. No.: CD003804.

PICO 2 Risk of bias assessment:

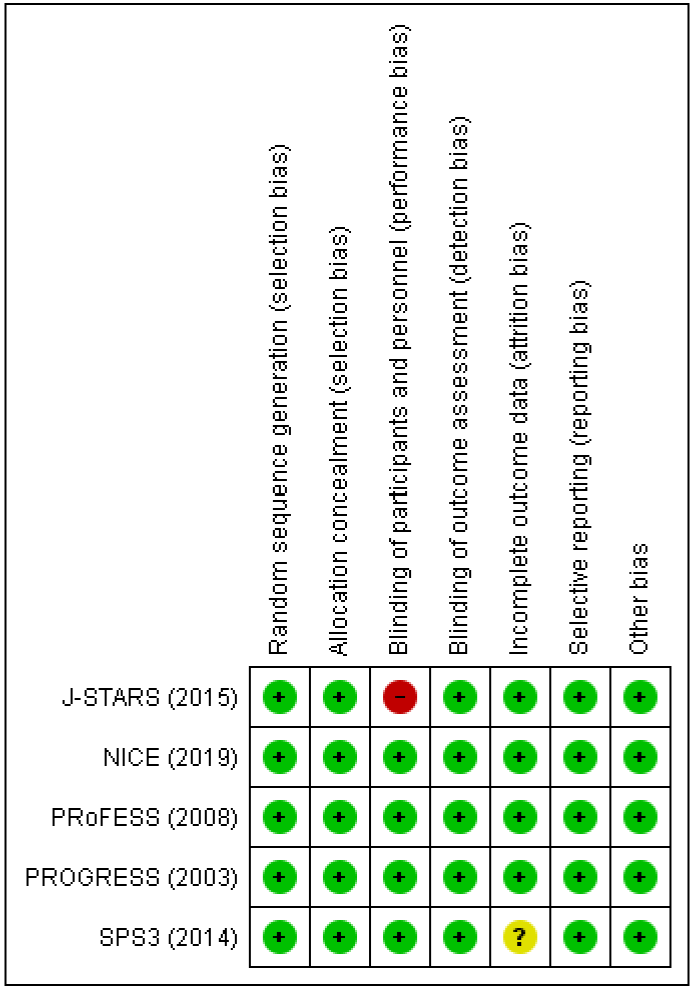

NB. The J-STARS trial was focused on statin treatment, NICE, PRoFESS and PROGRESS trials on antihypertensive medication and the SPS3 trial on both antihypertensive as antiplatelet therapy.

PICO 2 Summary of findings

Monitored intensive management of vascular risk factors compared to usual care for the prevention of post-stroke cognitive decline or dementia

Certainty assessment							Number of patients		Effect		Quality of evidence	Importance
Number of studies	Study design	Risk of bias	Inconsistency	Indirectness	Imprecision	Other	Intensive treatment	Usual care	Relative (95% CI)	Absolute (95% CI)		
**Dementia**												
3	RCT	not serious	serious [a]	serious [b]	serious [c]	none	633/12455 (5.1%)	659/12485 (5.3%)	**OR 0.96** (0.86 to 1.07)	**2 fewer per 1.000** (from 7 fewer to 3 more)	⊕◯◯◯ VERY LOW	Important
**Cognitive decline (assessed with: various tools)**												
5	RCT	not serious	serious [a]	serious [b]	serious [c]	none	The heterogeneity in interventions and outcomes precluded quantitative meta-analysis.				⊕◯◯◯ VERY LOW	Important

CI: confidence interval; OR: Odds ratio; RCT: Randomsied controlled trial

a. Inconsistency of dementia, mild cognitive impairment and cognitive decline outcomes

b. Heterogeneous interventions with differing pathophysiologic effects

c. Confidence intervals include both benefit and harm

d. Very heterogeneous operationalisation of cognitive decline

Summary of findings for PICO 2 Monitored intensive management of vascular risk factors compared to usual care for the prevention of post-stroke cognitive decline or dementia

PICO 3 PRISMA Flow chart

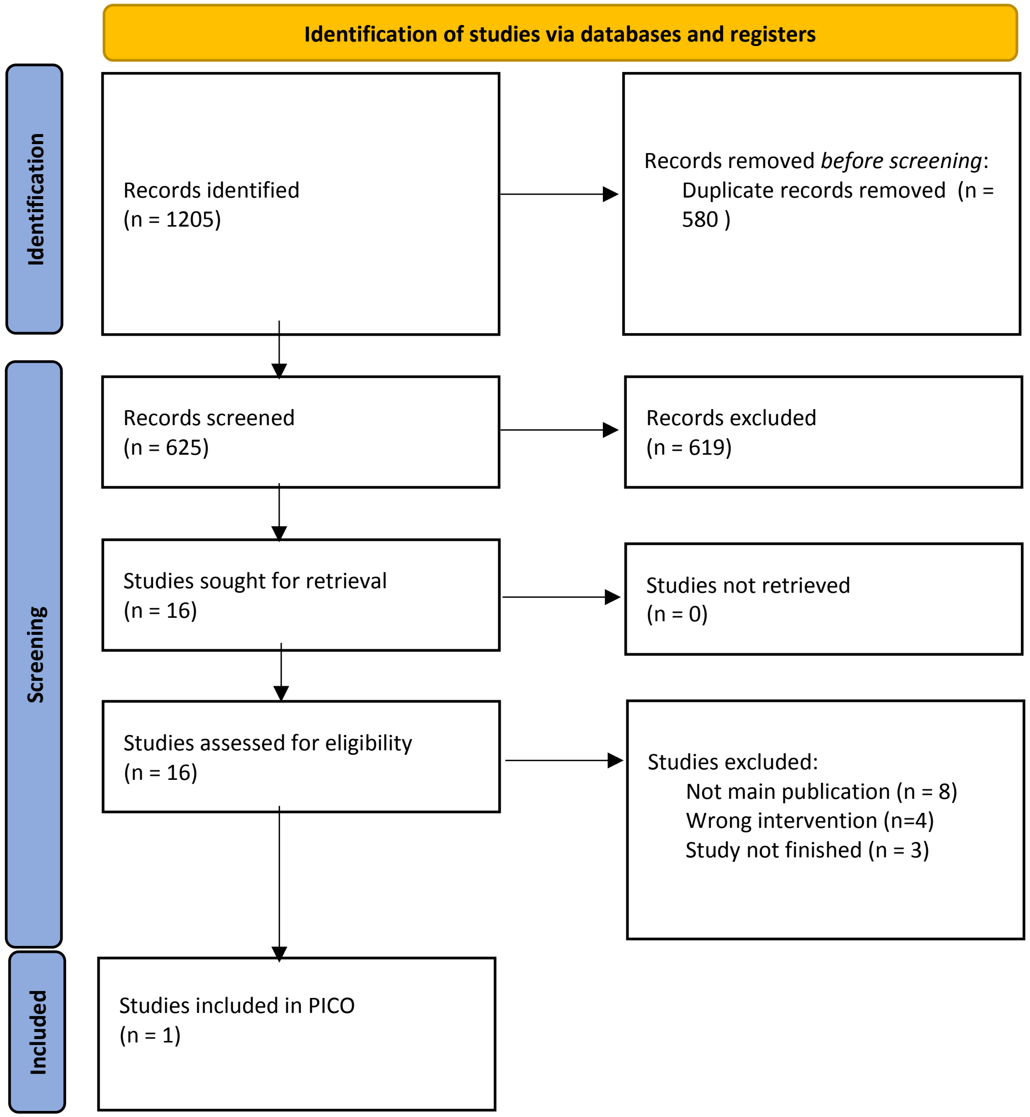

PICO 3 Risk of bias assessment

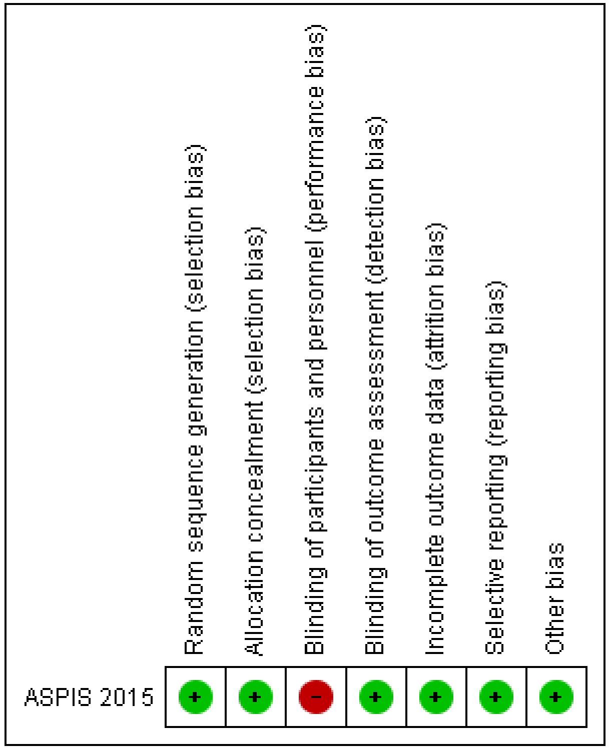

PICO 4 search syntax
In people with a history of stroke, **does cognitive training**, compared to usual care prevent: future cognitive decline or future dementia?

As pre-specified, where a high-quality systematic review had been recently published, We used the existing search strategy, but operated the inclusion/exclusion specific to this review question.
For this PICO we searched the included and excluded studies from the following reviews:

1. Gates NJ, Vernooij RWM, Di Nisio M, Karim S, March E, Martínez G, Rutjes AWS. Computerised cognitive training for preventing dementia in people with mild cognitive impairment. Cochrane Database of Systematic Reviews 2019, Issue 3. Art. No.: CD012279.
2. Gates NJ, Rutjes AWS, Di Nisio M, Karim S, Chong LY, March E, Martínez G, Vernooij RWM. Computerised cognitive training for 12 or more weeks for maintaining cognitive function in cognitively healthy people in late life. Cochrane Database of Systematic Reviews 2020, Issue 2. Art. No.: CD012277.
3. Lampit A, Hallock H, Valenzuela M. Computerized cognitive training in cognitively healthy older adults: a systematic review and meta-analysis of effect modifiers. PLoS Medicine 2014;11(11):e1001756.
4. Shao Y, Mang J, Li P, Wang J, Deng T, Xu Z. Computer-based cognitive programs for improvement of memory, processing speed and executive function during age-related cognitive decline: a meta-analysis. PLoS One 2015;10(6):e0130831.

PICO 6 Search syntax

In patients with stroke, **does routine use of cognitive assessment**, compared to no routine screening, improve stroke care.

1. cerebrovascular disorders/ OR exp basal ganglia cerebrovascular disease/ OR exp brain ischemia/ OR exp intracranial arterial diseases/ OR exp "intracranial embolism and thrombosis"/ OR exp intracranial hemorrhages/ OR stroke/ OR exp brain infarction/ OR vasospasm, intracranial/
2. (stroke OR post?stroke OR cerebrovasc$ OR brain vasc$ OR cerebral vasc$ OR cva$ OR apoplex$ OR SAH).tw.
3. ((brain$ OR cerebr$ OR cerebell$ OR intracran$ OR intracerebral) adj5 (isch?emi$ OR infarct$ OR thrombo$ OR emboli$ OR occlus$)).tw.
4. ((brain$ OR cerebr$ OR cerebell$ OR intracerebral OR intracranial OR subarachnoid) adj5 (h?emorrhage$ OR h?ematoma$ OR bleed$)).tw.
5. ((transi$ adj3 isch?em$ adj3 attack$) OR TIA$1).tw.
6. Or/1-5
7. exp Dementia
8. Delirium, Dementia, Amnestic, Cognitive Disorders/ or Cognitive Dysfunction
9. dement*.mp.
10. alzheimer*.mp.
11. (chronic adj2 cerebrovascular).mp.
12. ("organic brain disease" or "organic brain syndrome").mp.
13. 1"benign senescent forgetfulness".mp.
14. (cerebr* adj2 deteriorat*).mp.
15. (cerebral* adj2 insuEicient*).mp.
16. ("major neurocognitive disorder*" or "Cognitive Impairment and Disability").ti,ab.
17. Or/7-16
18. 6 And 17
19. Randomized Controlled Trials as Topic/
20. randomized controlled trial/
21. Random Allocation/

22. Double Blind Method/
23. Single Blind Method/
24. clinical trial/
25. clinical trial, phase i.pt
26. clinical trial, phase ii.pt
27. clinical trial, phase iii.pt
28. clinical trial, phase iv.pt
29. controlled clinical trial.pt
30. randomized controlled trial.pt
31. multicenter study.pt
32. clinical trial.pt
33. exp Clinical Trials as topic/
34. or/19-33
35. (clinical adj trial$).tw
36. ((singl$ or doubl$ or treb$ or tripl$) adj (blind$3 or mask$3)).tw
37. PLACEBOS/
38. placebo$.tw
39. randomly allocated.tw
40. (allocated adj2 random$).tw
41. or/35-40
42. 34 or 41
43. case report.tw
44. letter/
45. historical article/
46. or/43-45
47. 41 not 46

48. 18 AND 47
49. (predict* adj3 (dement* or AD or alzheimer*)).ti,ab.
50. (identif* adj3 (dement* or AD or alzheimer*)).ti,ab.
51. (discriminat* adj3 (dement* or AD or alzheimer*)).ti,ab.
52. (distinguish* adj3 (dement* or AD or alzheimer*)).ti,ab.
53. (differenti* adj3 (dement* or AD or alzheimer*)).ti,ab.
54. diagnos*.ti.
55. di.fs.
56. (detect* adj3 (dement* or AD or alzheimer*)).ti,ab.
57. accura*.ti,ab.
58. Or/48-56
59. 48 AND 58

PICO 6 PRISMA Flow chart

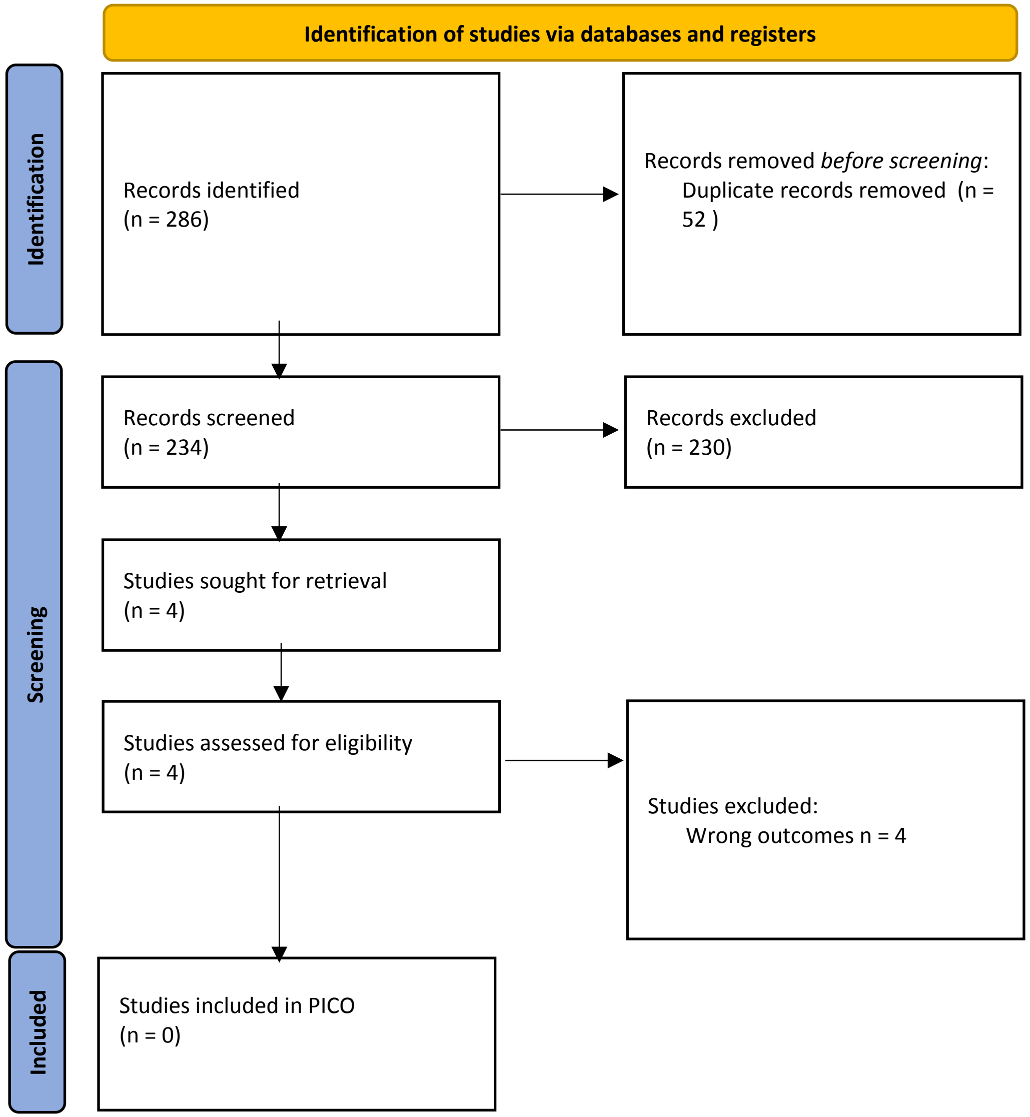

PICO 7-9 search syntax

PICO 7: In patients with stroke (acute or post-acute), what is the accuracy of **Montreal Cognitive Assessment** for contemporaneous diagnosis of post-stroke cognitive impairment or dementia?

PICO 8: In patients with stroke (acute or post-acute), what is the accuracy of **Folstein's Mini-Mental State Examination** for contemporaneous diagnosis of dementia?

PICO 9: In patients with stroke (acute or post-acute), what is the accuracy of **Addenbrooke's Cognitive Examination (ACE)** for contemporaneous diagnosis of dementia?

<u>Stroke</u>

1. cerebrovascular disorders/ OR exp basal ganglia cerebrovascular disease/ OR exp brain ischemia/ OR exp intracranial arterial diseases/ OR exp "intracranial embolism and thrombosis"/ OR exp intracranial hemorrhages/ OR stroke/ OR exp brain infarction/ OR vasospasm, intracranial/
2. (stroke OR post?stroke OR cerebrovasc$ OR brain vasc$ OR cerebral vasc$ OR cva$ OR apoplex$ OR SAH).tw.
3. ((brain$ OR cerebr$ OR cerebell$ OR intracran$ OR intracerebral) adj5 (isch?emi$ OR infarct$ OR thrombo$ OR emboli$ OR occlus$)).tw.
4. ((brain$ OR cerebr$ OR cerebell$ OR intracerebral OR intracranial OR subarachnoid) adj5 (h?emorrhage$ OR h?ematoma$ OR bleed$)).tw.
5. ((transi$ adj3 isch?em$ adj3 attack$) OR TIA$1).tw.
6. Or/1-5

<u>Test accuracy studies</u>

1. Exp "sensitivity and specificity"/
2. (sensitivity or specificity).tw.
3. (predictive adj3 value$).tw.
4. Exp Diagnostic errors/
5. ((false positive$) or (false negative$)).tw.
6. (observer variation$).tw.
7. (roc curve$).tw.
8. (likelihood adj3 ratio$).tw.
9. Likelihood functions/
10. Or/1-9

<u>MoCA</u>

1. “montreal cognitive assessment$”.mp.
2. MoCA.mp.
3. 1 or 2

<u>MMSE</u>

1. MMSE$.mp.
2. sMMSE.mp.
3. Folstein$.mp.
4. MiniMental.mp.
5. “mini mental stat$”.mp.
6. 3MS.mp.
7. Mini mental state examination/
8. Or/1-7

<u>Addenbrooke’s Cognitive Examination</u>

1. "Addenbrooke$ Cognitive Exam$".mp.
2. “Mini-Addenbrooke$ Cognitive Exam$”.mp.
3. ACE.mp.
4. ACE-r.mp.
5. Mini-ACE.mp.
6. ACE-III.mp.
7. Or/1-6

PICO 7-9 PRISMA flow diagram

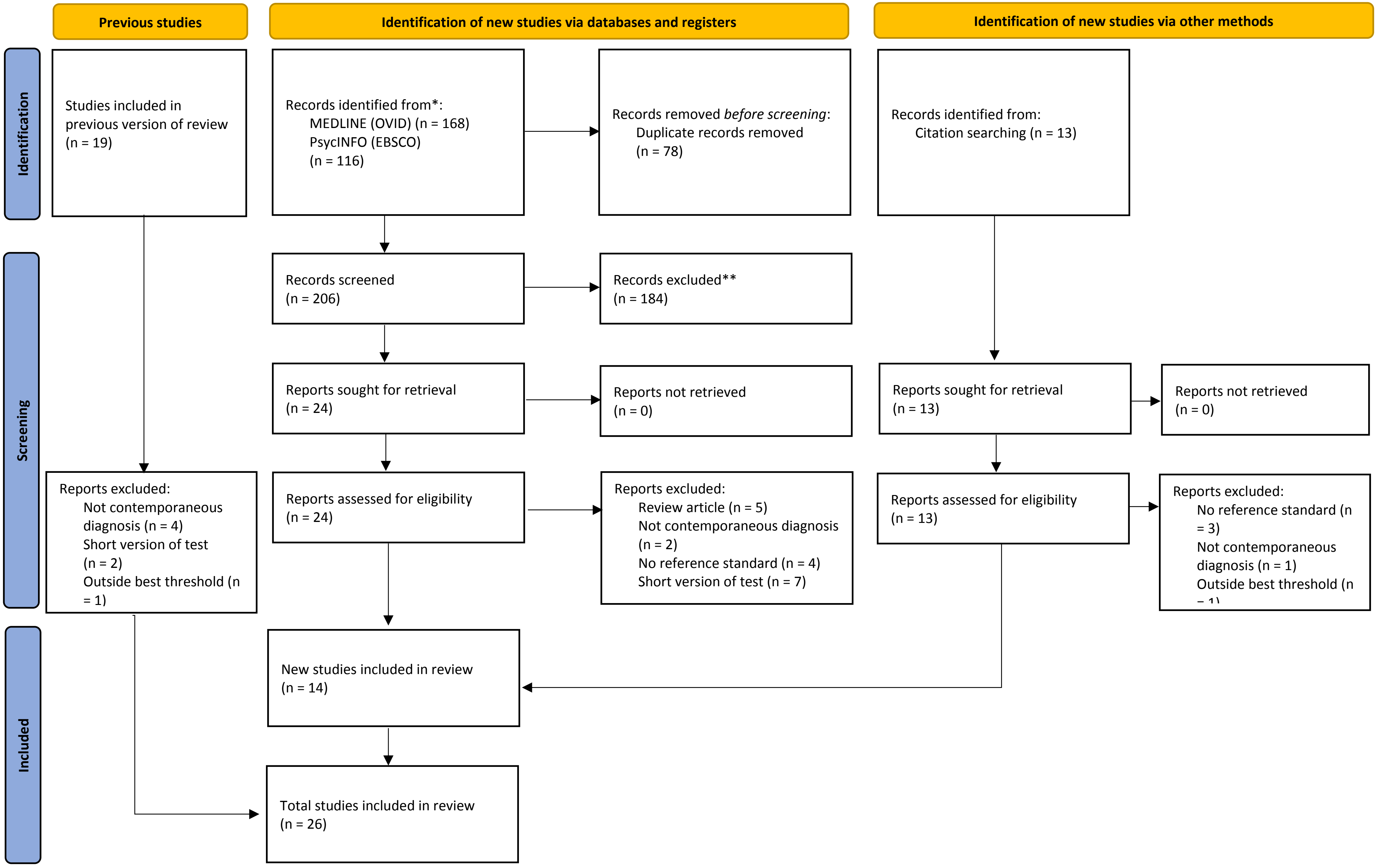

PICO 7 Risk of bias assessment

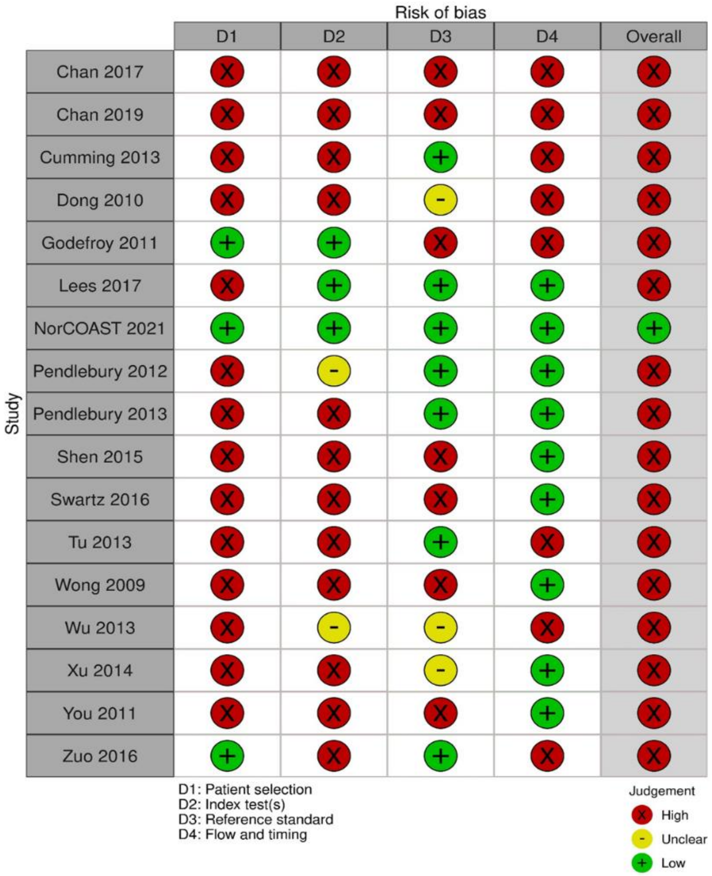

PICO 8 Risk of bias assessment

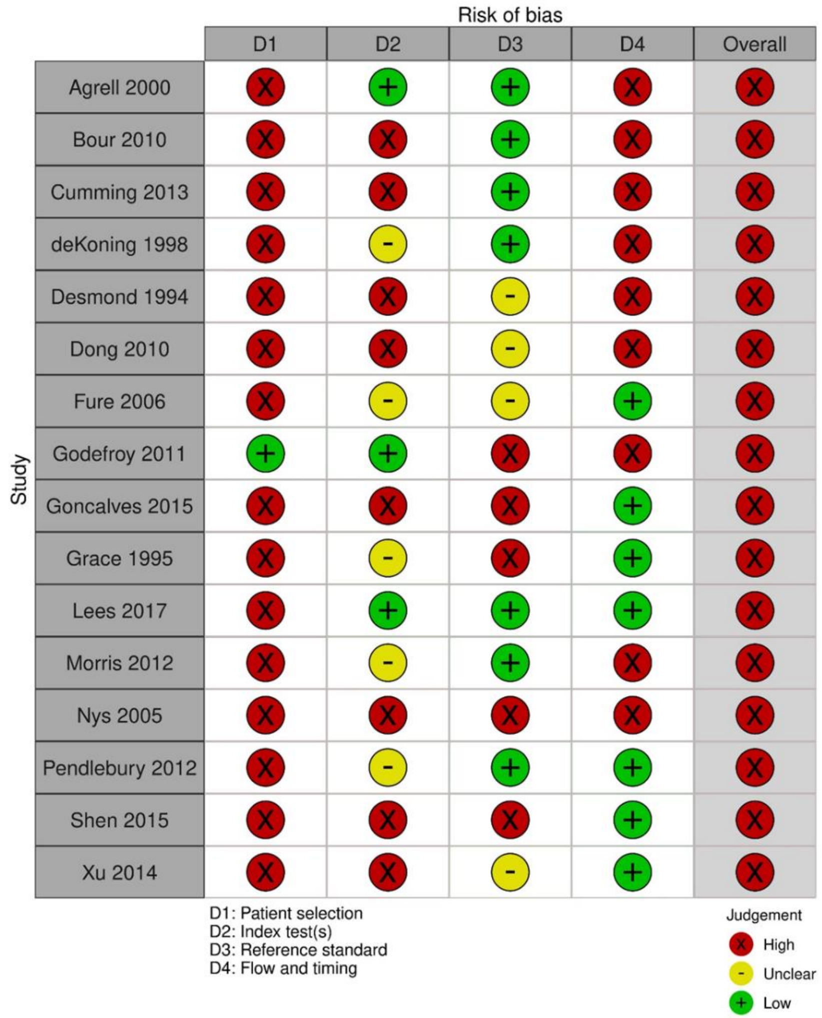

PICO 9 Risk of bias assessment

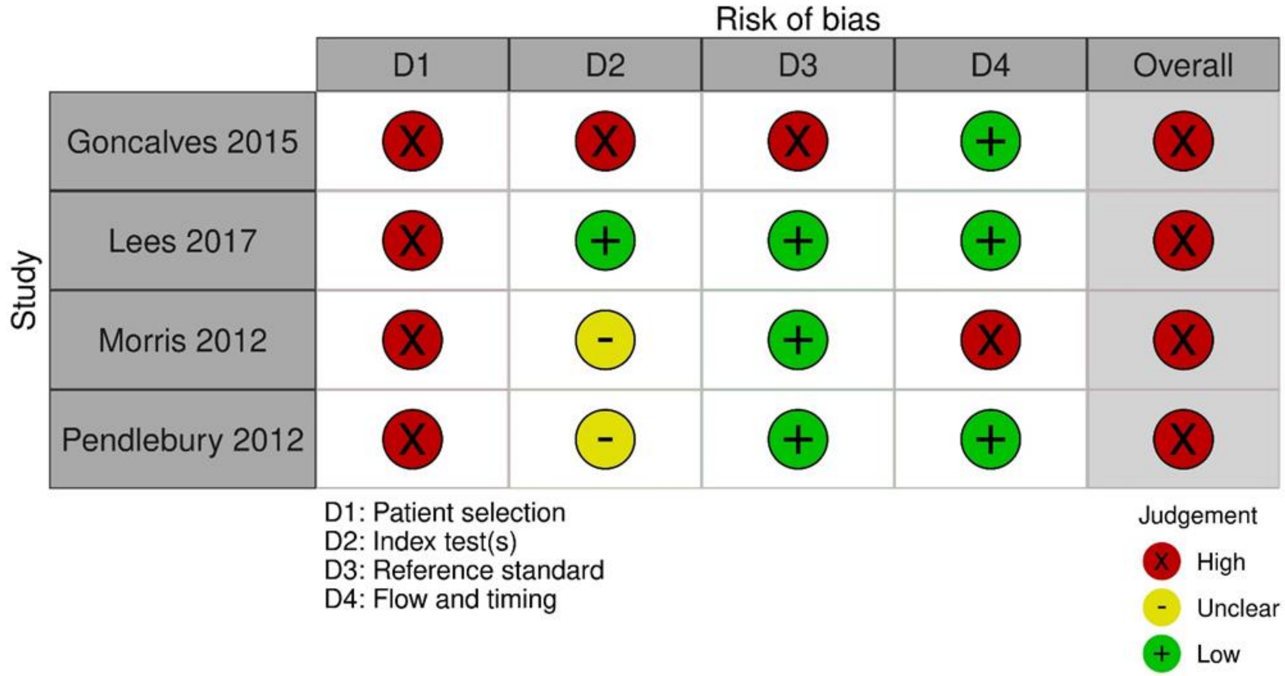

PICO 10 Search syntax

In patients with stroke (acute or post-acute), what is the accuracy of the **Oxford Cognitive Screen (OCS)** for contemporaneous diagnosis of dementia?

Stroke

1. cerebrovascular disorders/ OR exp basal ganglia cerebrovascular disease/ OR exp brain ischemia/ OR exp intracranial arterial diseases/ OR exp "intracranial embolism and thrombosis"/ OR exp intracranial hemorrhages/ OR stroke/ OR exp brain infarction/ OR vasospasm, intracranial/
2. (stroke OR post?stroke OR cerebrovasc$ OR brain vasc$ OR cerebral vasc$ OR cva$ OR apoplex$ OR SAH).tw.
3. ((brain$ OR cerebr$ OR cerebell$ OR intracran$ OR intracerebral) adj5 (isch?emi$ OR infarct$ OR thrombo$ OR emboli$ OR occlus$)).tw.
4. ((brain$ OR cerebr$ OR cerebell$ OR intracerebral OR intracranial OR subarachnoid) adj5 (h?emorrhage$ OR h?ematoma$ OR bleed$)).tw.
5. ((transi$ adj3 isch?em$ adj3 attack$) OR TIA$1).tw.
6. Or/1-5

Test accuracy studies

1. Exp “sensitivity and specificity”/
2. (sensitivity or specificity).tw.
3. (predictive adj3 value$).tw.
4. Exp Diagnostic errors/
5. ((false positive$) or (false negative$)).tw.
6. (observer variation$).tw.
7. (roc curve$).tw.
8. (likelihood adj3 ratio$).tw.
9. Likelihood functions/

10. Or/1-9

Oxford Cognitive Screen

1. “Oxford Cognitive Screen”
2. OCS.ti,ab
3. 1 or 2

PICO 10 PRISMA Flow chart

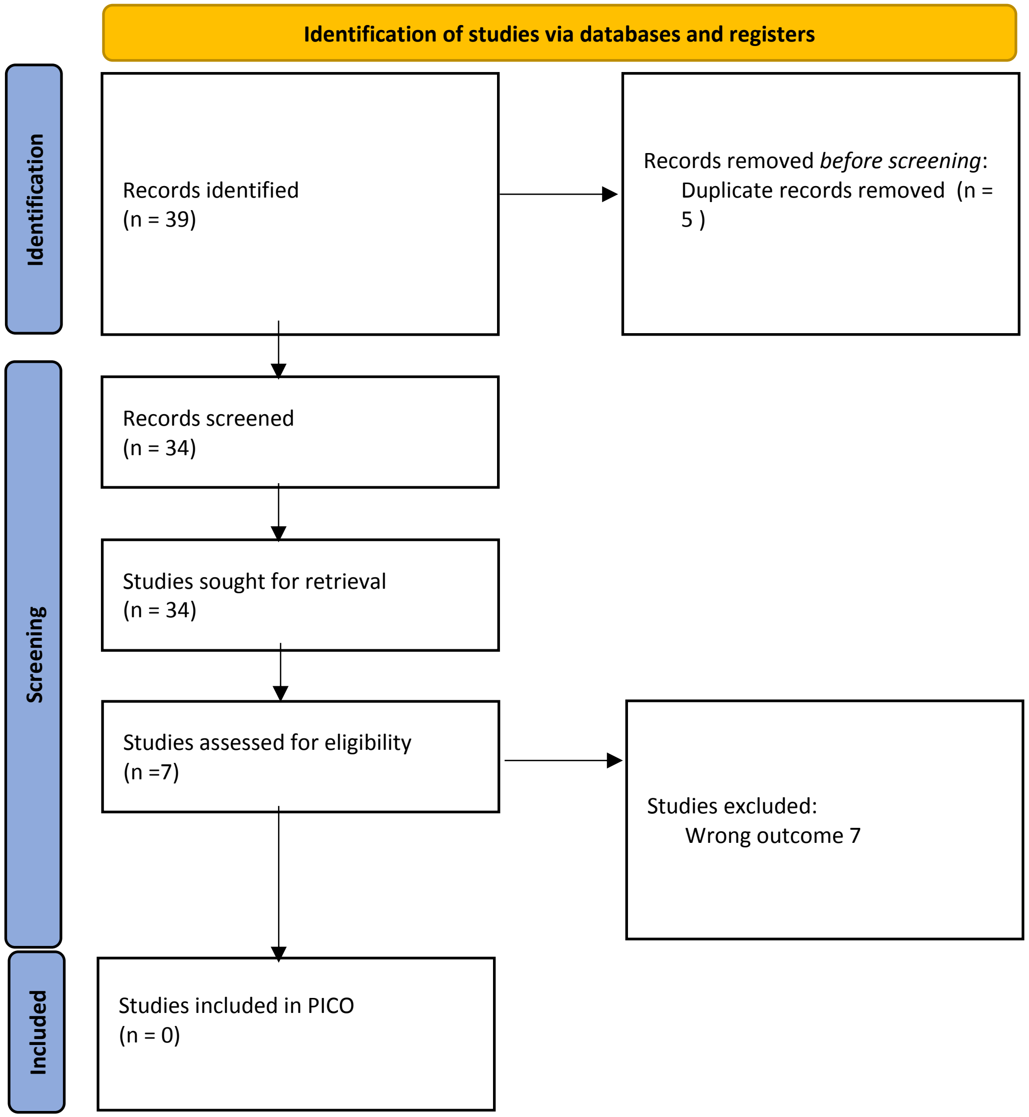

PICO 11 search syntax

In patients with stroke (acute or post-acute), what is the accuracy of the **remote assessment** for contemporaneous diagnosis of dementia?

As pre-specified, where a high-quality systematic review had been recently published, we used the existing search strategy, but operated the inclusion/exclusion specific to this review question.
For this PICO we searched the included and excluded studies from the following review:

Elliott E, Green C, llewellyn DJ, Quinn TJ. Accuracy of Telephone-Based Cognitive Screening Tests: Systematic Review and Meta-Analysis. Curr Alzheimer res.2020;17:460-471.

PICO 11 Risk of bias assessment

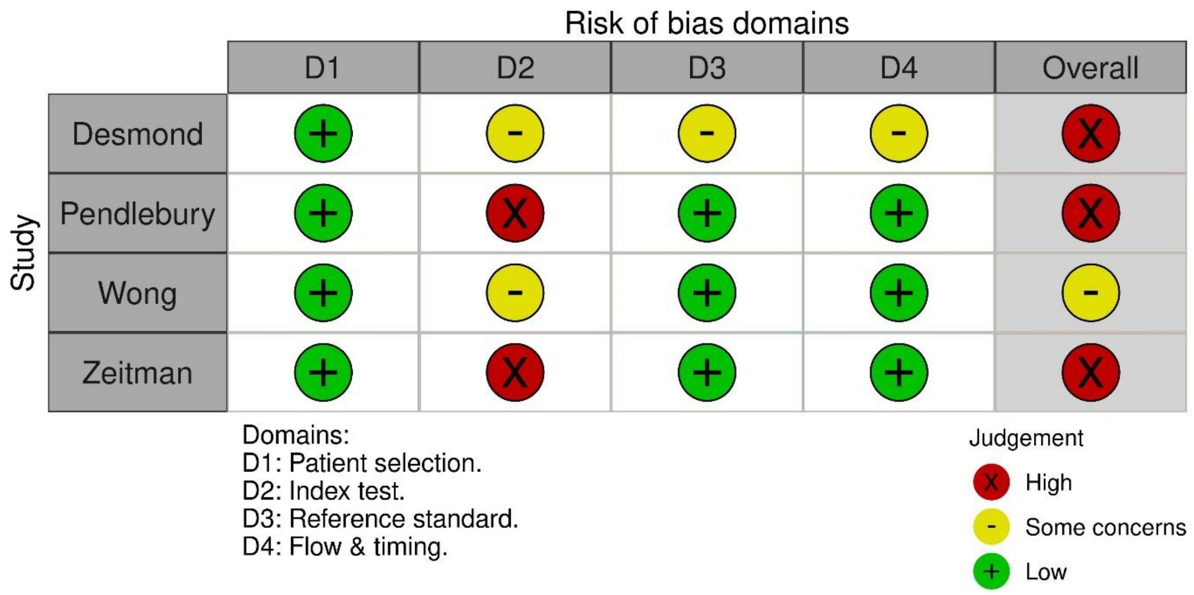

PICO 12-14 search syntax

PICO 12: In people with post-stroke cognitive impairments, do **cholinesterase inhibitors**, compared to placebo, delay cognitive decline or progression to dementia; improve behavioural and psychological symptoms, decrease caregiver burden and/or cause adverse effects?

PICO 13: In people with post-stroke cognitive impairments, does **memantine** compared to placebo, delay cognitive decline or progression to dementia, improve behavioural and psychological symptoms, decrease caregiver burden and/or cause adverse effects?

PICO 14: In people with post-stroke cognitive impairments, do the nootropics actovegin or cerebrolysin, compared to placebo improve cognitive decline, improve behavioural and psychological symptoms, reduce caregiver burden and/or increase adverse events.

As pre-specified, where a high-quality systematic review had been recently published, we used the existing search strategy, but operated the inclusion/exclusion specific to this review question.
For this PICO we searched the included and excluded studies from the following reviews:

Battle CE, Abdul-Rahim AH, Shenkin SD, Hewitt J, Quinn TJ. Cholinesterase inhibitors for vascular dementia and other vascular cognitive impairments: a network meta-analysis. Cochrane Database of Systematic Reviews 2021, Issue 2. Art. No.: CD013306.

McShane R, Westby MJ, Roberts E, Minakaran N, Schneider L, Farrimond LE, Maayan N, Ware J, Debarros J. Memantine for dementia. Cochrane Database of Systematic Reviews 2019, Issue 3. Art. No.: CD003154.

Li Y, Hai S, Zhou Y, Dong BR. Cholinesterase inhibitors for rarer dementias associated with neurological conditions. Cochrane Database of Systematic Reviews 2015, Issue 3. Art. No.: CD009444.

Alsulaimani RA, Quinn TJ. The efficacy and safety of animal-derived nootropics in cognitive disorders: Systematic review and meta-analysis. Cerebral Circulation - Cognition and Behavior. 2021; 2:100012.

Cui S, Chen N, Yang M, Guo J, Zhou M, Zhu C, He L. Cerebrolysin for vascular dementia. Cochrane Database of Systematic Reviews 2019, Issue 11. Art. No.: CD008900.

PICO 12 Risk of bias assessment

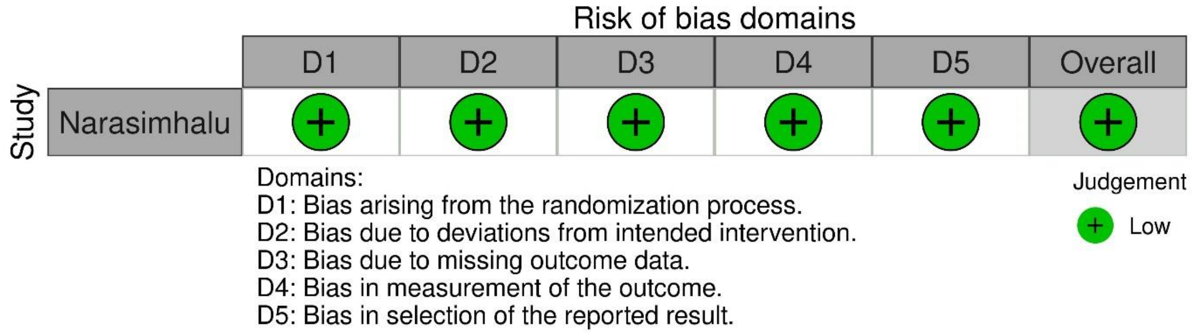

PICO 14 Risk of bias assessment

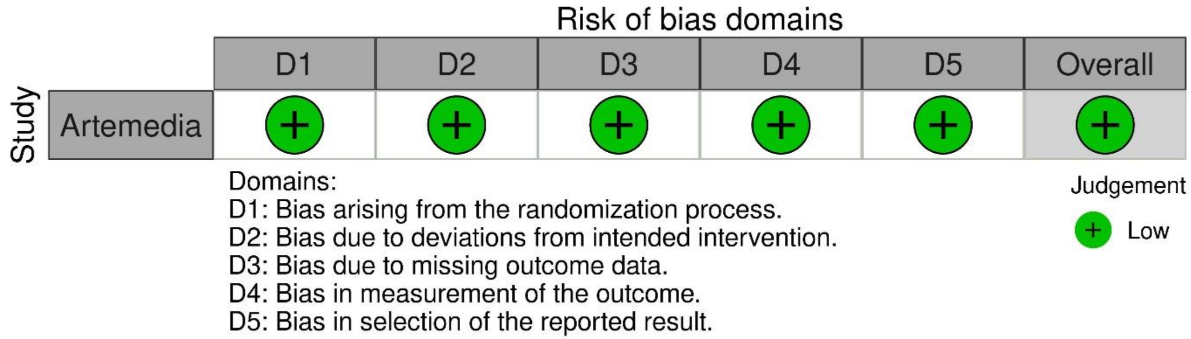

PICO 15 Search Syntax

In people with post-stroke cognitive impairments, does **cognitive rehabilitation (cognitive skill training or compensation strategies)** compared to no rehabilitation, delay cognitive decline or progression to dementia, improve behavioural and psychological symptoms, improve performance in activities of daily living or decrease caregiver burden?

We ran a bespoke search but cross referenced our results with the following reviews:

1. Rogers JM, Foord R, Stolwyk RJ, Wong D, Wilson PH. General and Domain-Specific Effectiveness of Cognitive Remediation after Stroke: Systematic Literature Review and Meta-Analysis. Neuropsychology review. 2018;28(3):285-309.
2. Ye M, Zhao B, Liu Z, Weng Y, Zhou L. Effectiveness of computer-based training on post-stroke cognitive rehabilitation: A systematic review and meta-analysis. Neuropsychological Rehabilitation. 2020.
3. Loetscher T, Potter KJ, Wong D, das Nair R. Cognitive rehabilitation for attention deficits following stroke. Cochrane Database of Systematic Reviews. 2019;11(11):10.

Stroke

S1: exp Brain Ischemia/px, th or (((brain or cerebral) adj2 (ischemia* or ischaemia* or infarct*)) or ((ischemic or ischaemic) adj2 stroke*)).ab,ti.

S2: exp "Intracranial Embolism and Thrombosis"/px, th or ((brain or cerebral or intracranial) adj2 (embol* or thromb*)).ab,ti.

S3: exp Cerebral Hemorrhage/px, th or (((brain or cerebral or cerebrum or intracerebral or intracortical) adj2 (hemorrhag* or haemorrhag*or bleeding or bleed)) or ((haemorrhagic or hemorrhagic) adj2 (apoplexy or stroke))).ab,ti.

S4: exp Stroke/px, th or (((cerebrovascular or cerebro vascular or cerebral) adj2 (accident* or apoplexy or apoplexia or stroke* or arrest* or failure* or injur* or insufficienc*)) or (brain adj2 vascular adj2 accident*) or apoplexy or CVA* stroke*).ab,ti.

S5: 1 or 2 or 3 or 4

Cognitive rehabilitation methods

S6: exp Cognitive Remediation/mt or ((cognitive or neuropsychological) adj2 (restitut* remediat* or restor* or train* or retrain* or re-train* or enhance* or rehabilitat* or intervention* or treat* or therap* or therapeutic* or practice or recover* or program* or (skill adj2 training) or technique*)).ab,ti.

S7: exp Cognitive Behavioral Therapy/mt or ((behavior* or behavior or cognitive or behavioural or behavioral) adj2 (therap* or psychotherapy or treatment*)).ab,ti.

S8: exp Therapy, Computer-Assisted/mt or ((computer assisted or computer-assisted) adj2 therap*).ab,ti.

S9: ((environmental adj2 (modification* or adaptation* or aid*)) or (compensatory adj2 (strateg* or mean*))).ab,ti.

S10: exp Stroke Rehabilitation/mt or (stroke adj2 rehabilitation).ab,ti.

S11: S6 - S10 / OR

Cognitive impairments (global + domain-specific)

S12: ((exp Cognition/ not exp Cognition/de) or (cognition* or awareness or ((brain or cognitive or mental) adj2 (function* or skill* or abilit* or reserve* or task* or capacity* or performance*)) or comprehension or understanding or consciousness* or meta cognition or meta-cognition or metacognition or meta emotion* or metaemotion* or meta-emotion* or meta memor* or meta-memor* or metamemor* or ((awareness* or knowledge* or control* or monitoring*) adj2 (meta-cognitive or meta cognitive or metacognitive))).ab,ti.) adj4 (decline* or dysfunction* or impair* or deterioration* or disorder* or deficit* or problem* or difficult* or disabilit* or disturbance* or disruption* or diminish* or reduc* or decreas* or limit* or distortion* or defect* or loss*).ab,ti.

S13: exp Cognitive Dysfunction/px, th or ((cognition or cognitive or mental or neurocognitive) adj2 (decline* or dysfunction* or impair* or deterioration* or disorder* or deficit* or problem* or difficult* or disabilit* or disturbance* or disruption* or diminish* or reduc* or decreas* or limit* or distortion* or defect*or loss*)).ab,ti.

S14: (((exp Arousal/ not exp Arousal/de) or (arousal* or (cortical adj2 vigilance) or attention or attentional or concentration or (information adj2 speed adj2 processing) or alertness or awareness or consciousness).ab,ti.) adj4 (decline* or dysfunction* or impair* or deterioration* or disorder* or deficit* or problem* or difficult* or disabilit* or disturbance* or disruption* or impair* or diminish* or reduc* or decrease* diminish* or reduc* or decreas* or limit* or distortion* or defect* or loss*).ab,ti.) or (inattention or distractibility or distracted or (mental adj2 slowness) or hemi-neglect or hemineglect or hemi-

inattention or neglect or hemiagnosia or hemispatialagnosia or ((unilateral or spatial or hemi-spatial or hemispatial or visuo-spatial or visuospatial or visual-spatial or contralateral or syndrome) adj2 (neglect or agnosia))).ab,ti.

S15: (((exp Executive Function/ not exp Executive Function/de) or (executive adj2 (control* or function* or processing or skill* or functioning)).ab,ti.) adj3 (decline* or dysfunction* or impair* or deterioration* or disorder* or deficit* or problem* or difficult* or disabilit* or disturbance* or disruption* or diminish* or reduc* or decreas* or limit* or distortion* or defect* or loss*).ab,ti.) or (dysexecutive adj2 (syndrome*or symptom* or function*)).ab,ti.

S16: (((exp intention/ or (intention* or intent).ab,ti.) OR exp Goals/ OR (((goal* or objective*) adj2 (setting or articulation or management or selection)) or planning or plan* or (goal-directed adj2 (behaviour or behavior or activit*)) or initiation or initiating or sequencing or (sequence adj2 steps)).ab,ti.) adj4 (decline* or dysfunction* or impair* or deterioration* or disorder* or deficit* or problem* or difficult* or disabilit* or disturbance* or disruption* or diminish* or reduc* or decreas* or limit* or distortion* or defect* or loss*).ab,ti.) or (aspontaneity or (action adj2 (disorganization OR disorganization))).ab,ti.

S17: (((inhibition or control or self-restraint) adj2 (decline* or dysfunction* or impair* or deterioration* or disorder* or deficit* or problem* or difficult* or disabilit* or disturbance* or disruption* or diminish* or reduc* or decreas* or limit* or distortion* or defect* or loss*)) or ((utilization adj2 (behavior or behaviour)) or distractibility or distracted or impulsive or impulsiveness or impulsivity or perseveration or disinhibition or aggression or aggressive or restlessness)).ab,ti.

S18: ((exp Problem Solving/ not exp Problem Solving/de) or (exp Thinking/ not exp Thinking/de) or (thinking or (decision adj2 making) or goal-setting or objective-setting or ((goal or objective) adj2 setting) or (problem adj2 solving) or judgement* or reasoning or self-instruction or self-regulation or self-regulatory or self-awareness or self-monitoring or monitoring or self-evaluation).ab,ti.) adj4 (decline* or dysfunction* or impair* or deterioration* or disorder* or deficit* or problem* or difficult* or disabilit* or disturbance* or disruption* or diminish* or reduc* or decreas* or limit* or distortion* or defect*).ab,ti.

S19: ((exp Memory/ not exp Memory/de, pd) or (memory or memories or recall* or retention or remembering).ab,ti.) adj2 (decline* or dysfunction* or impair* or deterioration* or disorder* or deficit* or problem* or difficult* or disabilit* or disturbance* or disruption* or diminish* or reduc* or decreas* or limit* or distortion* or defect* or loss*).ab,ti.

S20: exp Memory Disorders/px, th or (amnesia* or (amnestic adj2 state) or (memory adj2 (decline* or dysfunction* or impair* or deterioration* or disorder* or deficit* or problem* or difficult* or disabilit* or disturbance* or disruption* or diminish* or reduc* or decreas* or limit* or distortion* or defect* or loss*))).ab,ti.

S21: ((exp Perception/ not exp Perception/de, pd) or (perception* or perceptual or (perceptual adj2 (performance or processing or task* or skill* or ability* or function*)) or (body adj2 (image* or representation* or schema*)) or stereops* or (stereoscopic adj2 vision*) or stereognos*).ab,ti.) adj4 (decline* or dysfunction* or impair* or deterioration* or disorder* or deficit* or problem* or difficult* or disabilit* or disturbance* or disruption* or diminish* or reduc* or decreas* or limit* or distortion* or defect* or loss*).ab,ti.

S22: ((exp Spatial Navigation/ not exp Spatial Navigation/de) or ((space or spatial or visual) adj2 (perception or abilit* or discrimination or orientation or navigation or visualization or visualization or localization or localisation)).ab,ti.) adj4 (decline* or dysfunction* or impair* or deterioration* or disorder* or deficit* or problem* or difficult* or disabilit* or disturbance* or disruption* or diminish* or reduc* or decreas* or limit* or distortion* or defect* or loss*).ab,ti.

S23: exp Perceptual Disorders/px, th or (((perceptual or perceptive or perception) adj2 (decline* or dysfunction* or impair* or deterioration* or disorder* or deficit* or problem* or difficult* or disabilit* or disturbance* or disruption* or diminish* or reduc* or decreas* or limit* or distortion* or defect* or loss*)) or agnosia or neglect or anosognosia or disorientation or disorientation).ab,ti.

S24: exp Apraxias/th or (apraxia* or dyspraxia* or (awkward adj2 movement*) or clumsiness or perseveration or ((alien or anarchic) adj2 hand adj2 syndrome*)).ab,ti.

S25: S12 – S24 / OR

Study type

S26: exp adaptive clinical trial/ or exp clinical trial, phase i/ or exp clinical trial, phase ii/ or exp clinical trial, phase iii/ or exp clinical trial, phase iv/ or exp controlled clinical trial/ or exp randomized controlled trial/ or exp equivalence trial/ or exp pragmatic clinical trial/ or (((controlled OR clinical OR intervention OR therapeutic OR single-blind or double-blind OR triple-blind OR preventive or prophylactic or randomized or randomized or non-inferiority or noninferiority or non-superiority or nonsuperiority or superiority or equivalence or community or pragmatic or adaptive) adj3 (trial* or stud* or experiment* or comparison*)).ab,ti.)

S27: (adaptive clinical trial or clinical trial phase i or clinical trial phase ii or clinical trial phase iii or clinical trial phase iv or controlled clinical trial or equivalence trial or multicenter study or pragmatic clinical trial or randomized controlled trial).pt.

S28: S26 or S27

PICO 15 PRISMA Flow chart

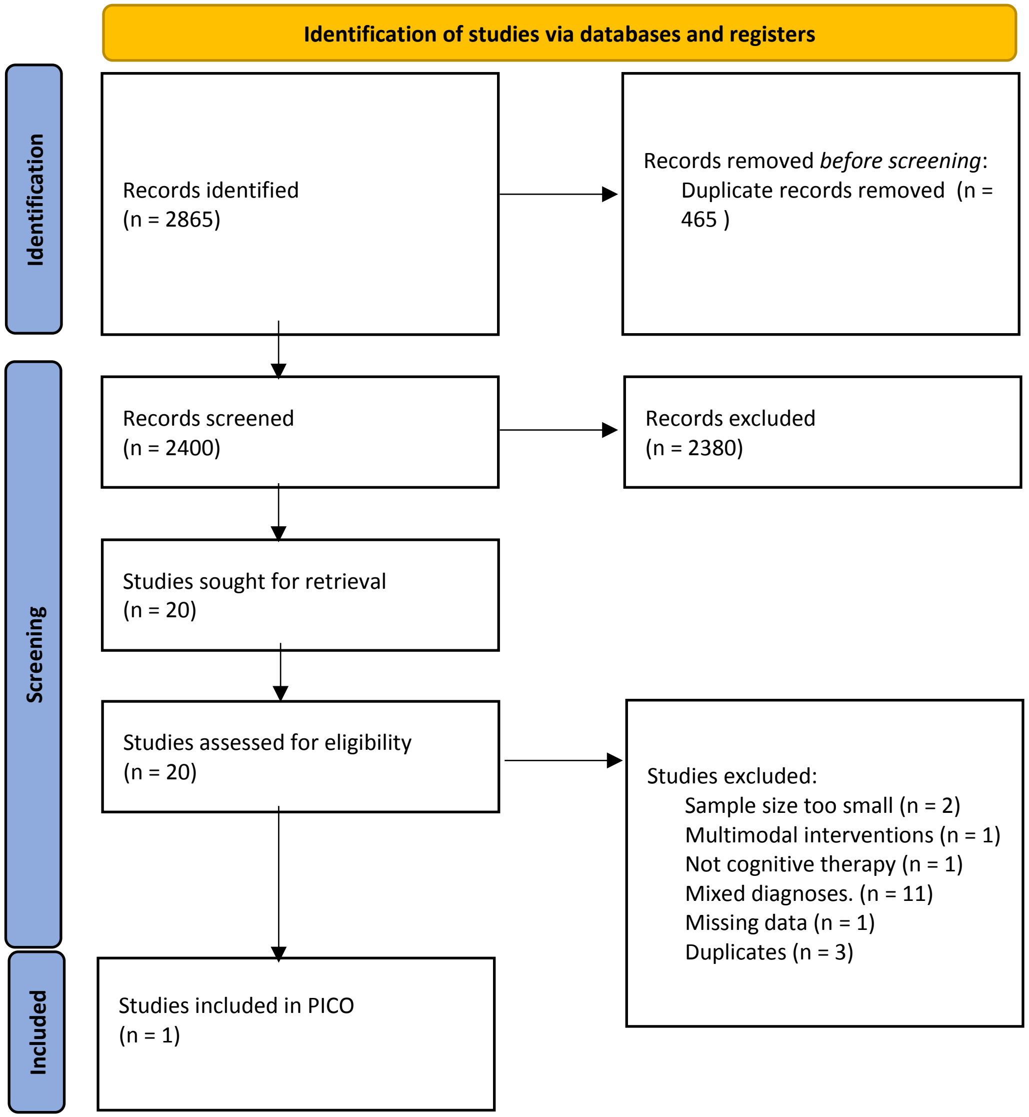

PICO 15 Risk of bias assessment

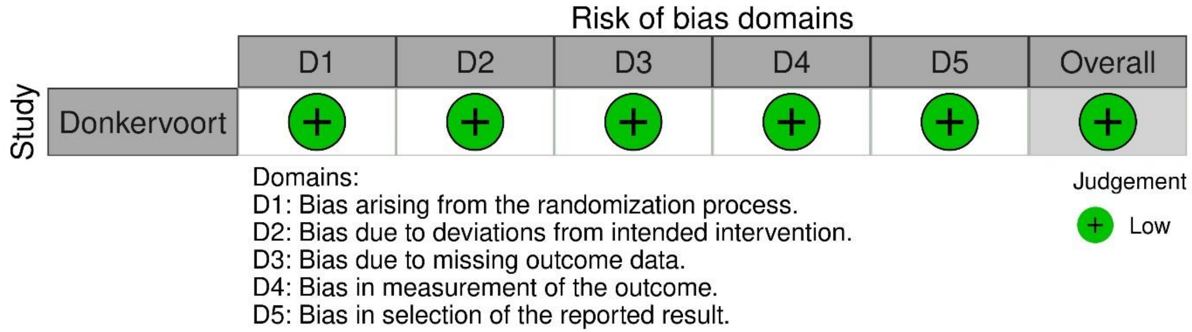

PICO 16. Search Syntax

In people with a history of stroke, do **multi-item prognostic tools** performed soon after stroke, predict future cognitive decline or dementia.

1. cerebrovascular disorders/ OR exp basal ganglia cerebrovascular disease/ OR exp brain ischemia/ OR exp intracranial arterial diseases/ OR exp "intracranial embolism and thrombosis"/ OR exp intracranial hemorrhages/ OR stroke/ OR exp brain infarction/ OR vasospasm, intracranial/
2. (stroke OR post?stroke OR cerebrovasc$ OR brain vasc$ OR cerebral vasc$ OR cva$ OR apoplex$ OR SAH).ti,ab.
3. ((brain$ OR cerebr$ OR cerebell$ OR intracran$ OR intracerebral) adj5 (isch?emi$ OR infarct$ OR thrombo$ OR emboli$ OR occlus$)).ti,ab.
4. ((brain$ OR cerebr$ OR cerebell$ OR intracerebral OR intracranial OR subarachnoid) adj5 (h?emorrhage$ OR h?ematoma$ OR bleed$)).ti,ab.
5. ((transi$ adj3 isch?em$ adj3 attack$) OR TIA$1).ti,ab.
6. 1 OR 2 OR 3 OR 4 OR 5
7. ((validat$ OR predict$ OR prognos$ OR rule$) adj3 (outcome$ OR risk$ OR model$)).ti,ab.
8. (prognos$ AND (method$ OR history OR variable$ OR criteria OR scor$ OR characteristic$ OR finding$ OR factor$ OR model$)).ti,ab.

9. ((history OR variable$ OR criteria OR scor$ OR characteristic$ OR finding$ OR factor$) adj3 (predict$ OR model$ OR decision$ OR identif$ OR prognos$)).ti,ab.

10. (decision$ adj3 (model$ OR clinical$)).ti,ab.

11. (stratification OR discriminat$ OR calibration).ti,ab.

12. ROC curve/

13. (c-statistic OR c statistic OR area under the curve OR AUC).ti,ab.

14. (indices OR algorithm OR multivariable).ti,ab.

15. 7 OR 8 OR 9 OR 10 OR 11 OR 12 OR 13 OR 14

16. exp dementia/

17. delirium/

18. delirium, dementia, amnestic, cognitive disorders/

19. exp cognition disorders/

20. exp cognition/

21. memory/

22. dement$.ti,ab.

23. (Alzheimer$ OR AD).ti,ab.

24. deliri$.ti,ab.

25. ((cognit$ OR memory OR mental OR brain) adj3 (func$ OR perform$ OR abilit$ OR declin$ OR reduc$ OR impair$ OR disorder$ OR fail$ OR los$ OR deficit$ OR stop$ OR

progress$ OR improve$)).ti,ab.

26. mental perform$.ti,ab.

27. (memory adj3 (complain$ or declin$ or function$)).ti,ab.

28. 16 OR 17 OR 18 OR 19 OR 20 OR 21 OR 22 OR 23 OR 24 OR 25 OR 26 OR 27

29. 6 AND 15 AND 28

PICO 16 PRISMA flow diagram

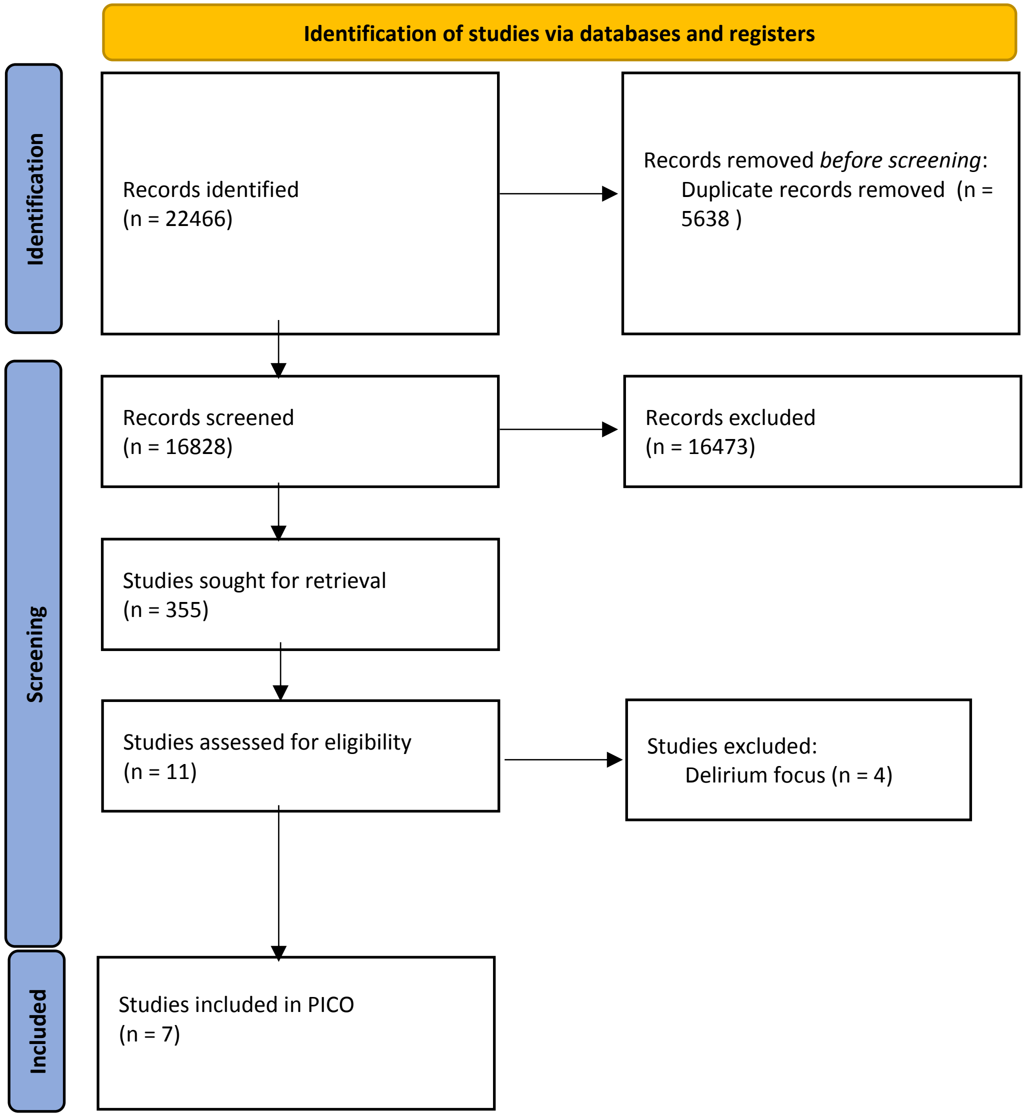

PICO 16 Risk of bias assessment

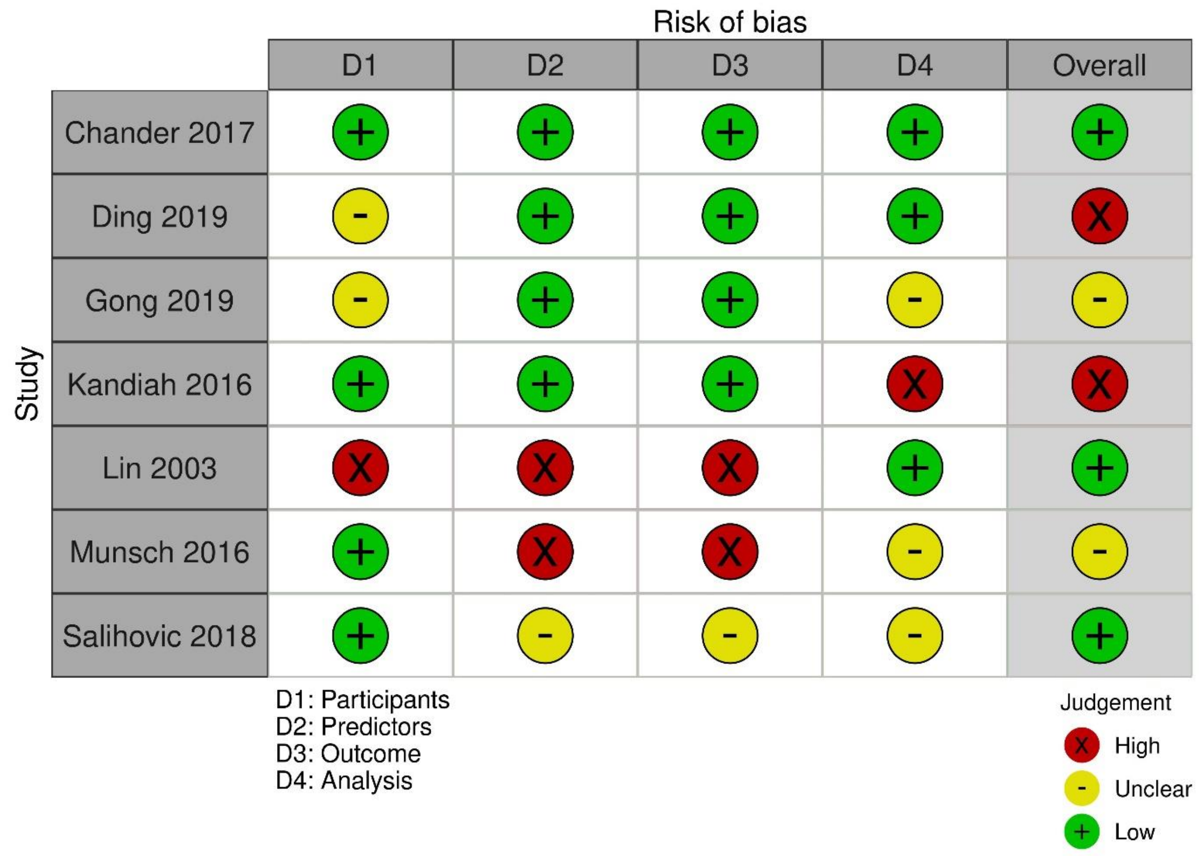

PICO 17 and 18 Search syntax

PICO 17: In people with a history of stroke, do **structural features on acute brain CT imaging,** predict (at least one year from index stroke event) future cognitive decline or dementia.

PICO 18 In people with a history of stroke, do **structural features on acute brain MR imaging**, predict (at least one year from index stroke event) future cognitive decline or dementia.

Stroke

1. cerebrovascular disorders/ or exp basal ganglia cerebrovascular disease/ or brain ischemia/ or hypoxia-ischemia, brain/ or ischemic attack, transient/ or carotid artery diseases/ or carotid artery thrombosis/ or intracranial arterial diseases/ or cerebral arterial diseases/ or exp "intracranial embolism and thrombosis"/ or exp intracranial hemorrhages/ or exp stroke/
2. (stroke$ or apoplex$ or cerebral vasc$ or cerebrovasc$ or cva or transient isch$mic attack$ or tia).tw.
3. ((brain or cerebr$ or cerebell$ or hemispher$ or intracran$ or intracerebral or infratentorial or supratentorial or space-occupying) adj5 (isch?emi$ or infarct$ or thrombo$ or emboli$ or occlus$ or hypoxi$)).tw.
4. ((brain or cerebr$ or cerebell$ or hemispher$ or intracran$ or intracerebral or infratentorial or supratentorial or intraventricular) adj5 (h?emorrhag$ or h?ematoma$ or bleed$)).tw.
5. or/1-4

Dementia and cognitive impairment

6. exp dementia/ or memory disorders/ or neurocognitive disorders/ or cognition disorders/ or cognitive dysfunction/
7. dement$.tw.

8. ((attention or awareness or cognit$ or neuropsych$ or neurocognit$ or neurobehav$ or psycholog$ or memor$ or recall or think$) adj5 (declin$ or impair$ or domain$ or test$ or assess$ or function$ or batter$ or disorder$ or dysfunct$ or deficit$ or declin$ or abilit$ or problem$ or difficult$ or disturbance$ or disabilit$)).tw.
9. mental processes/ or cognition/ or awareness/ or cognitive reserve/ or executive function/ or learning/ or thinking/ or perception/ or memory/
10. exp psychological tests/
11. MMSE.tw.
12. or/6-11

Neuroimaging

13. diagnostic imaging/ or neuroimaging/ or multimodal imaging/
14. tomography, x-ray computed/
15. ((compute$ or cerebral or CAT or CT or brain) adj5 (imag$ or scan$ or neuroimag$ or tomogra$ or marker$ or feature$ or x-ray)).tw.
16. Magnetic resonance imaging/
17. (MRI or MRi or NMRI or NMRi).tw.
18. ((magn$ or resonance or MR or MTC or MT or NMR) adj5 (imag$ or scan$ or neuroimag$ or tomogra$ or marker$ or feature$)).tw.
19. or/13-18

Study type

20. prognosis/
21. observational study/
22. randomized controlled trial.pt.
23. controlled clinical trial.pt.
24. Epidemiologic Studies/
25. exp Case-Control Studies/
26. exp Cohort Studies/
27. (epidemiologic adj (study or studies)).ab,ti.

28. case control.ab,ti.
29. (cohort adj (study or studies)).ab,ti.
30. cohort analy$.ab,ti.
31. (follow up adj (study or studies)).ab,ti.
32. longitudinal.ab,ti.
33. retrospective$.ab,ti.
34. prospective$.ab,ti.
35. (observ$ adj3 (study or studies)).ab,ti.
36. or/20-35

Running the search

37. 5 and 12 and 19 and 36

PICO 17 and 18 PRISMA Flow chart

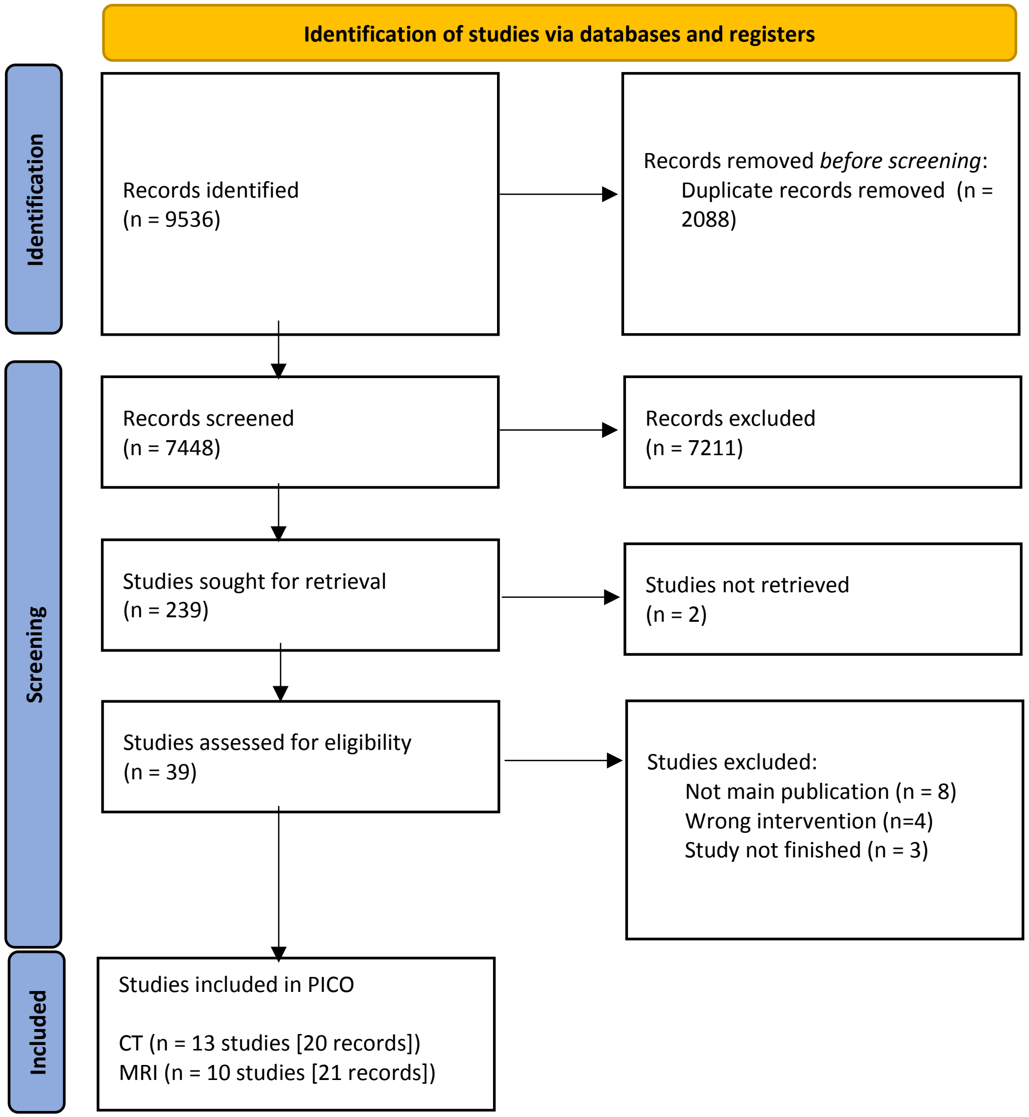

PICO 17 Risk of bias assessment

Risk of bias

Study	D1	D2	D3	D4	D5	D6	Overall
Alexandrova (2016)	-	?	+	+	X	-	-
Andersen (1996)	-	?	+	+	-	-	-
Biffi (2016)	+	-	+	-	+	+	-
Bornstein (1996)	-	+	+	+	X	-	-
Chausson (2010)	-	?	+	+	X	-	-
Cordoliani-Mackowiak (2003)	-	?	+	-	+	+	-
House (1990)	-	?	+	+	X	+	-
Loeb (1992)	X	+	+	+	X	-	-
Mehrabian (2015)	-	?	+	+	X	+	-
Miyao (1992)	X	+	+	+	X	+	-
Moulin (2016)	+	?	+	-	+	+	+
Rasquin (2004)	-	?	X	+	+	+	-
Renjen (2015)	-	?	+	+	X	X	-

D1: Study participation
D2: Study attrition
D3: Prognostic factor measurement
D4: Outcome measurement
D5: Adjustment for other prognostic factors
D6: Statistical analysis and reporting

Judgement
X High
- Moderate
+ Low
? No information

PICO 18 Risk of bias assessment

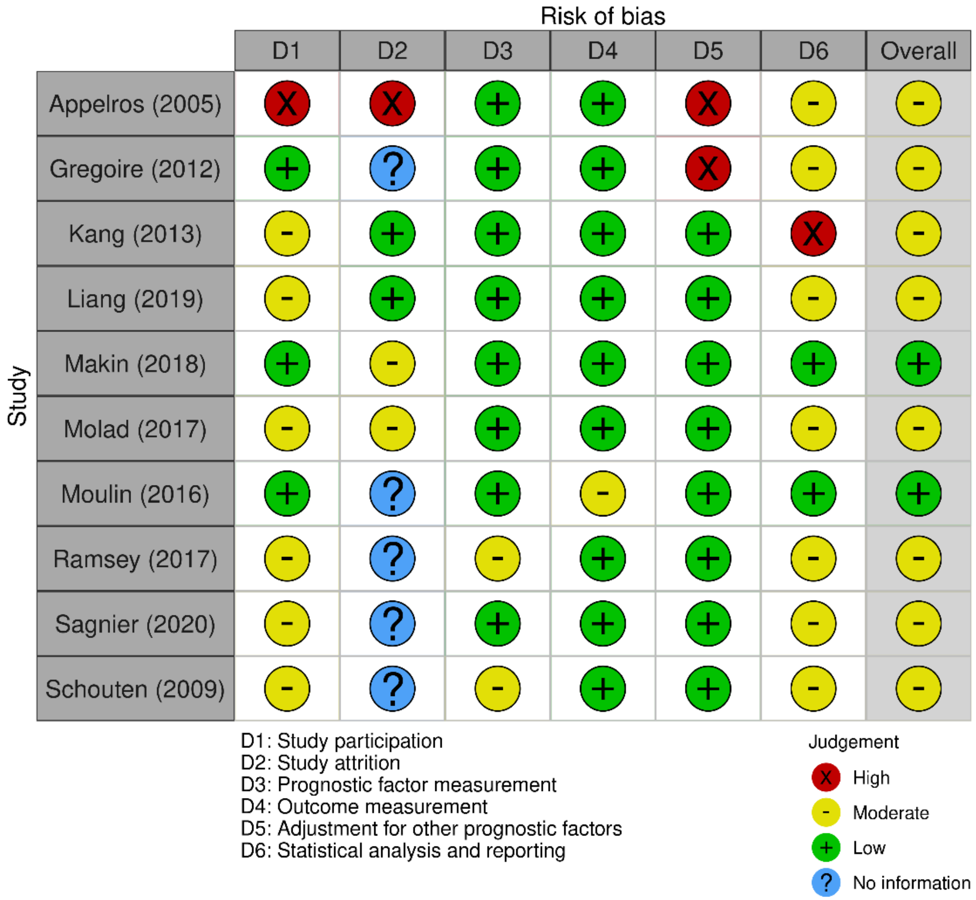

PICO 17 Summary of included studies

Study	Study N	Stroke type	Follow-up	Excluded prior dementia/CI	Excluded prior stroke	Criteria for PSD	Criteria for PSCI	CT-imaging features
**Alexandrova (2016)**	47	IS	12 months	dementia	no	-	MMSE<24	Acute stroke features
**Andersen (1996)**	127	IS + H	12 months	dementia	no	-	MDRS<27	Acute stroke features, atrophy
**Biffi (2016)**	445	H	Median 47.4 months	dementia	no	ICD-9 codes in electronic medical records and/or TICS-m > 20	-	Acute stroke features, WML
**Bornstein (1996)**	158	IS	biannual follow-up to 5 years	cognitive impairment	yes	DSM-III-R	-	SBI
**Chausson (2010)**	229	IS + H	5 years	no	no	-	MMSE<11	Acute stroke features, WML
**Cordoliani-Mackowiak (2003)**	144	IS + H	6, 12, 24, 36 months	dementia	no	ICD-10	-	Acute stroke features, atrophy, SBI, WML
**House (1990)**	115	IS	12 months	no	yes	-	MMSE<24	Acute stroke features, atrophy, WML
**Loeb (1992)**	106	lacunar	Avg 4 years	dementia	no	DSM-III-R and MMSE<24	-	Acute stroke features, Atrophy, WML
**Mehrabian (2015)**	74	IS	12 months	cognitive impairment	yes	-	Winblad et al. criteria and impairment in one or more cognitive domains.	Acute stroke features, Atrophy, WML
**Miyao (1992)**	215	lacunar	Average 25-29 months	dementia	yes	DSM-III-R and MMSE < 20	-	WML
**Moulin (2016)**	218	H	Median 6 years	Cognitive impairment	no	NIAA	-	Acute stroke features, atrophy*, WML*
**Rasquin (2004)**	144	IS	12 months	dementia or MMSE greater than or equal to 15	yes	DSM-IV	at least one cognitive deficit	Acute stroke features, atrophy, SBI, WML
**Renjen (2015)**	50	IS+H	12 months	PGI-BBD>18	no	-	PGI-BBD≥18, DSM used to diagnose dementia	Acute stroke features, atrophy, SBI, WML

PICO 18 Summary of included studies

Study	Study n	Age, years+	Stroke type	Excluded prior stroke	Excluded prior dementia/MCI	Cognitive assessment
Appelros (2005)	81	Mean=66.4 years	LACS	Y	N	MMSE
Gregoire (2012)	55	With microbleeds 65 (44-86) Without microbleeds 62 (33-75)	IS	N	Y, "non cerebrovascular diseases known to impair cognitive function"	Neuropsychological battery
Kang (2013)	408	Mean 64.7 years	IS	N	Y	MMSE
Liang (2019)	1821 451 at 15 months	Mean/SD=66.0/10.3 years	IS	Y	Y, dementia	MMSE
Makin (2018)	208	Median (IQR)=66 (56-74)	LACS	N	Y, dementia and MCI	ACE-R
Molad (2017)	572 266 at 12 months	Mean/SD=66.4/9.4 years	IS and TIA	Y	N	Neuropsychological battery
Moulin (2016)	218	Median/IQR=67.5/55.0-76.0 years	H	N	Y, cognitive impairment	NIAA for all-cause dementia
Ramsey (2017)	132	Mean/SD=54/11 years	IS + H	Y	Y, dementia	Neuropsychological battery
Sagnier (2020)	207	Mean/SD=66/13 years	IS	N	Y, dementia	MoCA, Isaacs set test, Zazzo cancellation task
Schouten (2009)	105	Mean/SD=63.0/14.2 years	IS	Y	Y, dementia	Rey auditory verbal learning test Doors test recognition

References

1. McGuinness, LA, Higgins, JPT. Risk-of-bias VISualization (robvis): An R package and Shiny web app for visualizing risk-of-bias assessments. Res Syn Meth. 2020; 1- 7. https://doi.org/10.1002/jrsm.1411
2. Hafdi M, Hoevenaar-Blom MP, Richard E. Multi-domain interventions for the prevention of dementia and cognitive decline. Cochrane Database of Systematic Reviews 2020, Issue 4. Art. No.: CD013572. DOI: 10.1002/14651858.CD013572
3. Hughes D, Judge C, Murphy R, et al. Association of Blood Pressure Lowering With Incident Dementia or Cognitive Impairment: A Systematic Review and Meta-analysis. JAMA. 2020;323(19):1934–1944.
4. Zonneveld TP, Richard E, Vergouwen MDI, Nederkoorn PJ, de Haan RJ, Roos YBWEM, Kruyt ND. Blood pressure-lowering treatment for preventing recurrent stroke, major vascular events, and dementia in patients with a history of stroke or transient ischaemic attack. Cochrane Database of Systematic Reviews 2018, Issue 7. Art. No.: CD007858.
5. Cholesterol treatment Trialists Collaboration. Efficacy and safety of statin therapy in older people: a meta-analysis of individual participant data from 28 randomised controlled trials. Lancet. 2019; 393:407-415
6. McGuinness B, Craig D, Bullock R, Passmore P. Statins for the prevention of dementia. Cochrane Database of Systematic Reviews 2016, Issue 1. Art. No.: CD003160.
7. Jordan F, Quinn TJ, McGuinness B, Passmore P, Kelly JP, Tudur Smith C, Murphy K, Devane D. Aspirin and other non-steroidal anti-inflammatory drugs for the prevention of dementia. Cochrane Database of Systematic Reviews 2020, Issue 4. Art. No.: CD011459.
8. Areosa Sastre A, Vernooij RWM, González-Colaço Harmand M, Martínez G. Effect of the treatment of Type 2 diabetes mellitus on the development of cognitive impairment and dementia. Cochrane Database of Systematic Reviews 2017, Issue 6. Art. No.: CD003804.
9. Gates NJ, Vernooij RWM, Di Nisio M, Karim S, March E, Martínez G, Rutjes AWS. Computerised cognitive training for preventing dementia in people with mild cognitive impairment. Cochrane Database of Systematic Reviews 2019, Issue 3. Art. No.: CD012279.
10. Gates NJ, Rutjes AWS, Di Nisio M, Karim S, Chong LY, March E, Martínez G, Vernooij RWM. Computerised cognitive training for 12 or more weeks for maintaining cognitive function in cognitively healthy people in late life. Cochrane Database of Systematic Reviews 2020, Issue 2. Art. No.: CD012277.
11. Lampit A, Hallock H, Valenzuela M. Computerized cognitive training in cognitively healthy older adults: a systematic review and meta-analysis of effect modifiers. PLoS Medicine 2014;11(11):e1001756.
12. Shao Y, Mang J, Li P, Wang J, Deng T, Xu Z. Computer-based cognitive programs for improvement of memory, processing speed and executive function during age-related cognitive decline: a meta-analysis. PLoS One 2015;10(6):e0130831.

13. Elliott E, Green C, llewellyn DJ, Quinn TJ. Accuracy of Telephone-Based Cognitive Screening Tests: Systematic Review and Meta-Analysis. Curr Alzheimer res.2020;17:460-471.
14. Battle CE, Abdul-Rahim AH, Shenkin SD, Hewitt J, Quinn TJ. Cholinesterase inhibitors for vascular dementia and other vascular cognitive impairments: a network meta-analysis. Cochrane Database of Systematic Reviews 2021, Issue 2. Art. No.: CD013306.
15. McShane R, Westby MJ, Roberts E, Minakaran N, Schneider L, Farrimond LE, Maayan N, Ware J, Debarros J. Memantine for dementia. Cochrane Database of Systematic Reviews 2019, Issue 3. Art. No.: CD003154.
16. Li Y, Hai S, Zhou Y, Dong BR. Cholinesterase inhibitors for rarer dementias associated with neurological conditions. Cochrane Database of Systematic Reviews 2015, Issue 3. Art. No.: CD009444.
17. Alsulaimani RA, Quinn TJ. The efficacy and safety of animal-derived nootropics in cognitive disorders: Systematic review and meta-analysis. Cerebral Circulation - Cognition and Behavior. 2021; 2:100012.
18. Cui S, Chen N, Yang M, Guo J, Zhou M, Zhu C, He L. Cerebrolysin for vascular dementia. Cochrane Database of Systematic Reviews 2019, Issue 11. Art. No.: CD008900.
19. Rogers JM, Foord R, Stolwyk RJ, Wong D, Wilson PH. General and Domain-Specific Effectiveness of Cognitive Remediation after Stroke: Systematic Literature Review and Meta-Analysis. Neuropsychology review. 2018;28(3):285-309.
20. Ye M, Zhao B, Liu Z, Weng Y, Zhou L. Effectiveness of computer-based training on post-stroke cognitive rehabilitation: A systematic review and meta-analysis. Neuropsychological Rehabilitation. 2020.
21. Loetscher T, Potter KJ, Wong D, das Nair R. Cognitive rehabilitation for attention deficits following stroke. Cochrane Database of Systematic Reviews. 2019;11(11):10.
