# Supplementary material for: European Stroke Organisation and European Academy of Neurology joint guidelines on post-stroke cognitive impairment
Source: Eur Stroke J. 2021 Oct 8;6(3):I–XXXVIII. doi: 10.1177/23969873211042192 (PMC8564156; doi:10.1177/23969873211042192)
Supplement: sj-pdf-2-eso-10.1177_23969873211042192 – Supplemental Material for European Stroke Organisation and European Academy of Neurology joint guidelines on post-stroke cognitive impairment [file sj-pdf-2-eso-10.1177_23969873211042192.pdf]

**PICO 1:** In people with a history of stroke, do **monitored lifestyle-based interventions (exercise, dietary change, alcohol moderation, weight loss, smoking cessation), alone or in combination,** compared to care as usual, prevent: future cognitive decline or dementia?
**Recommendation**<br><br>We cannot recommend monitored lifestyle interventions solely for the prevention of post-stroke cognitive decline or dementia.<br><br>Quality of evidence: **Very Low ⨁**<br>Strength of recommendation: No recommendation<br>*(This recommendation only relates to the cognitive effects of lifestyle interventions)*
**Expert Consensus Statement**<br><br>Lifestyle interventions, alone or in combination, should not be used solely for the prevention of post-stroke cognitive decline or dementia. Other benefits, such as a better physical or mental health or the prevention of future cardiovascular disease may warrant recommendations on lifestyle after stroke, but these were not the focus of this guideline.<br><br>There is a need for further, adequately powered trials that assess the effect of monitored lifestyle interventions on cognitive outcomes following stroke.

PICO 2: In people with a history of stroke, does **monitored intensive management of vascular risk factors**, compared to usual care, prevent: future cognitive decline or dementia?

Recommendation

Blood pressure treatment.

We cannot recommend intensive treatment of blood pressure compared to usual care solely for the prevention of post-stroke cognitive decline and dementia.

Quality of evidence: **Very Low** ⊕

Strength of recommendation: No recommendation

(This recommendation only relates to cognitive effects of blood pressure treatment)

Antithrombotic therapy.

We suggest against using dual antiplatelet therapy compared to single antiplatelet therapy for the prevention of cognitive decline in lacunar stroke.

Quality of evidence: **Very Low** ⊕

Strength of recommendation: **Weak against intervention** ↑?

(This recommendation relates to cognitive effects of dual antiplatelet and is applicable to lacunar stroke only)

Statin treatment.

We cannot recommend intensive statin treatment compared to usual care solely for the prevention of post-stroke cognitive decline or dementia.

Quality of evidence: **Very Low** ⊕

Strength of recommendation: No recommendation

(This recommendation only relates to cognitive effects of statin treatment)

Expert Consensus Statement

Given the beneficial effects of vascular risk management on prevention of recurrent stroke and cardiovascular disease, comprehensive risk factor management including blood pressure reduction, antithrombotic and statin is warranted following stroke, even though the cognitive benefits are unclear.

Targets for stroke risk factor management are constantly evolving and approaches that were historically considered 'intensive' are now common practice and recommended in guidelines.

Future trials of secondary prevention in stroke should include cognitive outcome measures.

**PICO 3:** In people with a history of stroke, **do monitored multicomponent interventions (lifestyle and pharmacological)**, compared to usual care, prevent: future cognitive decline or dementia?
**Recommendation**<br><br>We cannot recommend multicomponent interventions (including medications and lifestyle interventions) solely for the prevention of post-stroke cognitive decline or dementia.<br><br>Quality of evidence: **Very Low ⊕**<br>Strength of recommendation: no recommendation<br>*(This recommendation only relates to the cognitive effects of multicomponent interventions)*
**Expert Consensus Statement**<br><br>All but one of the writing group agreed that:<br>Monitored multicomponent interventions, cannot be recommended for the prevention of cognitive decline or dementia following stroke alone, but there are other potential health benefits associated with these lifestyle interventions, such as the prevention of future cardiovascular disease or recurrent stroke.

**PICO 4:** In people with a history of stroke**, does cognitive training,** compared to usual care prevent: future cognitive decline or future dementia?
**Recommendation**<br><br>There is continued uncertainty over the benefits and limitations of cognitive training for the prevention of cognitive decline and dementia in people living with stroke.<br><br>Quality of evidence: **Very low**<br>Strength of recommendation: No recommendation
**Expert Consensus Statement**<br><br>All but one of the writing group agreed that:<br>Cognitive training could be considered following stroke as part of a broader rehabilitation package. However, based on the current available literature, there is no evidence that cognitive training, as a single intervention, has a clinically meaningful or sustained benefit for prevention of cognitive decline or dementia following stroke.

**PICO 5:** In people with a history of post-stroke dementia does**, stopping pharmacological management of vascular risk factors (de-prescribing),** compared to continuing these medications prevent: future cognitive decline or improve health related quality of life
**Recommendation**<br><br>There is continued uncertainty over the benefits and risks of continuing treatment with antihypertensive or statin medications compared to withdrawal of these medications for cognitive or quality of life outcomes in people living with post-stroke dementia.<br><br>Quality of evidence: **Very low**<br>Strength of recommendation: No recommendation
**Expert Consensus Statement**<br><br>Given the beneficial effect on cardiovascular disease/stroke prevention and no clear signal of cognitive harm, pharmacological vascular risk factor management should be continued in patients with mild to moderate post-stroke dementia.<br><br>In people living with more advanced dementia and short life expectancy, where the potential harms and burden of treatment may be greater than any vascular protection, the benefits of continuing stroke secondary prevention medications are unclear.<br><br>Pragmatic trials of deprescribing medications are needed to guide treatment decisions in people living with advanced post-stroke dementia.

**PICO 6:** In patients with stroke, **does routine use of cognitive assessment**, compared to no routine screening, improve stroke care.
**Recommendation**<br><br>Due to a lack of relevant trials in patients with stroke, there is continued uncertainty over the benefits and risks of routine cognitive screening to improve stroke care.<br><br>Quality of evidence: **Very Low ⊕**<br>Strength of recommendation: no recommendation<br>*(this recommendation applies only to routine screening of all patients presenting with stroke, and does not apply to clinician directed assessment)*
Expert Consensus Statement<br><br>Cognitive screening should be considered as part of the comprehensive assessment of stroke survivors.<br><br>However, there are insufficient data to make recommendations around the timing, the content or the potential benefits of cognitive screening to the patient, their care-givers, and to healthcare systems.<br><br>Further studies describing the effects of routine cognitive screening following stroke are required. These studies should include acute stroke settings, should record feasability and acceptability, consider effects on care pathways, and describe care-giver outcomes and health economics.

**PICO 7:** In patients with stroke (acute or post-acute), what is the accuracy of **Montreal Cognitive Assessment** for contemporaneous diagnosis of post-stroke cognitive impairment or dementia?
**Recommendation**<br><br>We suggest that in post-acute stroke settings, screening of cognition using the Montreal Cognitive Assessment (MoCA) is considered<br>MoCA should not be used as a substitute for comprehensive clinical assessment.<br><br>At the conventional threshold for test positivity, MoCA screening will detect most stroke survivors with important cognitive issues but at the cost of substantial false positives.<br>We suggest that a revised (lower) threshold be considered for stroke populations.<br><br>Quality of evidence: **Low** ⨁⨁<br><br>Strength of recommendation: **Weak for intervention** ↑?
**Expert Consensus Statement**<br><br>There are inherent limitations to the Montreal Cognitive Assessment (MoCA), which relies on intact visuospatial and language function for completion.<br><br>While the MoCA has acceptable test properties for use as an initial screening test in a stroke population, consideration should be given to the development of cognition screening tools that are more acceptable and feasible for those with communication difficulties or spatial neglect.<br><br>Those utilising the MoCA cognitive screening test should be fully trained in its administration.<br><br>Further comprehensive cognitive assessment is recommended in the event of a positive MoCA test result and findings should be shared with the stroke care team.

**PICO 8:** In patients with stroke (acute or post-acute), what is the accuracy of **Folstein's Mini-Mental State Examination** for contemporaneous diagnosis of dementia?
**Recommendation**<br><br>We suggest that in acute and post-acute stroke settings, screening of cognition using Folstein's Mini-Mental State Examination (MMSE) be considered.<br>MMSE should not be used as a substitute for comprehensive clinical assessment.<br><br>At the conventional threshold for test positivity, MMSE screening will exclude most stroke survivors with no important cognitive issues, but at the cost of substantial false negatives.<br><br>Quality of evidence: **Low ⨁⨁**<br>Strength of recommendation: **Weak for intervention** ↑?
**Expert Consensus Statement**<br><br>There are inherent limitations to the Mini Mental State Examination (MMSE), which relies on intact visuospatial and language function for completion.<br><br>While the MMSE has acceptable test properties for use as an initial screening test in a stroke population, consideration should be given to the development of cognition screening tools that are more acceptable and feasible for those with communication difficulties or spatial neglect.<br><br>Those utilising the MMSE cognitive screening test should be fully trained in its administration.<br><br>Further comprehensive cognitive assessment is recommended in the event of a positive MMSE test and findings should be shared with the stroke care team.

**PICO 9:** In patients with stroke (acute or post-acute), what is the accuracy of **Addenbrooke's Cognitive Examination (ACE)** for contemporaneous diagnosis of dementia?
**Recommendation**<br><br>We suggest that in acute and post-acute stroke settings, screening of cognition with one of the versions of the Addenbrookes Cognitive Examination (ACE) can be considered.<br>ACE should not be used as a substitute for comprehensive clinical assessment.<br><br>Test properties are sensitive to the threshold used to define test positivity, but there were insufficient data to make recommendations around the optimal cut-off for use in stroke.<br><br>Quality of evidence: **Very Low** ⊕<br>Strength of recommendation: **Weak for intervention** ↑?
**Expert Consensus Statement**<br><br>There are inherent limitations to the various versions of the Addenbrookes Cognitive Examination (ACE), which all rely on intact visuospatial and language function for completion.<br><br>Acceptable test properties for the ACE have not been established for use as an initial screening test in a stroke population and consideration should be given to the development of cognition screening tools that are more acceptable and feasible for those with communication difficulties or spatial neglect.<br><br>Those utilising the ACE cognitive screening test should be trained in its administration.<br><br>Further comprehensive cognitive assessment is recommended in the event of a positive ACE test result and findings should be shared with the stroke care team.

**PICO 10.** In patients with stroke (acute or post-acute), what is the accuracy of the **Oxford Cognitive Screen (OCS)** for contemporaneous diagnosis of dementia?
**Recommendation**<br><br>There is insufficient published evidence to assess the accuracy of the Oxford Cognitive Screen (OCS) for contemporaneous diagnosis of dementia in the stroke setting.<br><br>Future research should assess the diagnostic accuracy and utility of the OCS for post-stroke cognitve syndromes.<br><br>Quality of evidence: **Very Low** ⨁<br>Strength of recommendation: **no recommendation**
**Expert Consensus Statement**<br><br>The Oxford Cognitive Screen (OCS) offers advantages over other screening tools in terms of ease of completion and feasibility for stroke survivors with physical, language or visuospatial impairments.<br><br>Test accuracy studies of the OCS as a screen for post stroke dementia are required.<br><br>Those utilising the OCS should be trained in its administration.<br><br>Further comprehensive cognitive assessment is recommended in the event of a positive OCS and findings should be shared with the stroke care team.

**PICO 11.** In patients with stroke (acute or post-acute), what is the accuracy of **remote assessment** for contemporaneous diagnosis of dementia?
**Recommendation**<br><br>We suggest that in post-acute stroke settings, telephone-based screening of cognition can be considered.<br>Telephone-based cognitive screening is not a substitute for comprehensive clinical assessment.<br><br>At conventional thresholds for test positivity, telephone-based screening will detect most people with important cognitive issues but at the cost of substantial false positives.<br><br>Test properties are sensitive to the threshold used to define test positivity, but there were insufficient data to make recommendations around the optimal cut-off for use in stroke.<br><br>Quality of evidence: **Very Low** ⊕<br>Strength of recommendation: **Weak for intervention** ↑?
**Expert Consensus Statement**<br><br>There are inherent limitations to telephone based cognitive screening, but that telephone screening can be useful in situations where in-person assessment is not practical.<br><br>Video call based cognitive screening shows promise in stroke, but further studies and best practice guidance around application and interpretation of results is needed.<br><br>Consideration should be given to the development and validation of specific telephone or video call cognitive screening tools or protocols.<br><br>Those utilising remote cognitive screening tests should be trained in their administration.<br><br>Further comprehensive cognitive assessment is recommended in the event of a positive screening test result and findings should be shared with the stroke care team.

PICO 12: In people with post-stroke cognitive impairments, do **cholinesterase inhibitors**, compared to placebo, delay cognitive decline or progression to dementia; improve behavioural and psychological symptoms, decrease caregiver burden and/or cause adverse effects?

Recommendation

In people living with post-stroke cognitive impairment there is continued uncertainty over the benefits and risks of cholinesterase inhibitors for cognition, behavioural and psychological symptoms, activities of daily living and caregiver burden.

Quality of evidence: **Very Low ⊕**

Strength of recommendation: No recommendation possible

Expert Consensus Statement

In people living with post stroke dementia, any beneficial effect of cholinesterase inhibitors is likely to be modest, and perhaps not clinically relevant, the risk of adverse events should also be considered.

In a predominantly vascular cognitive impairment the effect of these drugs is minimal, but many older adults with stroke have other neurodegenerative diseases that may benefit from cholinesterase inhibitors.

We recognise that excluding co-existant Alzheimer's disease or other neurodegenerative processes can be difficult in older adults with stroke and if the diagnosis is of probable mixed pathology then cholinesterase inhibitors may be considered.

Stroke should not be a barrier to considering treatment with cholinesterase inhibitors if suspected concomitant Alzheimer's disease and Lewy Body dementia.

**PICO 13:** In people with post-stroke cognitive impairments, does **memantine** compared to placebo, delay cognitive decline or progression to dementia, improve behavioural and psychological symptoms, decrease caregiver burden and/or cause adverse effects?
**Recommendation**<br><br>In people living with post-stroke cognitive impairment there is continued uncertainty over the benefits and risks of memantine for cognition, behavioural and psychological symptoms, activities of daily living and caregiver burden.<br><br>Quality of evidence: **Very Low** ⊕<br>Strength of recommendation: no recommendation
**Expert Consensus Statement**<br><br>In people living with post stroke dementia, any beneficial effect of memantine is likely to be modest, and perhaps not clinically relevant, the risk of adverse events should also be considered.<br><br>In a predominantly vascular cognitive impairment, the effect of memantine is minimal, but many older adults with stroke have other neurodegenerative diseases that may benefit from this drug. We recognise that excluding co-existant Alzheimer's disease can be difficult in older adults with stroke and if the diagnosis is of probable mixed pathology then memantine may be considered.<br><br>Stroke should not be a barrier to considering treatment with memantine if suspected concomitant moderate to severe Alzheimer's disease.

PICO 14: In people with post-stroke cognitive impairments, do the nootropics **actovegin or cerebrolysin**, compared to placebo improve cognitive decline, improve behavioural and psychological symptoms, reduce caregiver burden and/or increase adverse events.

Recommendation

In patients with post-stroke cognitive impairment there is continued uncertainty over the benefits and risks of actovegin.

Quality of evidence: **Very Low** ⊕

Strength of recommendation: No recommendation

In patients with post-stroke cognitive impairment there is continued uncertainty over the benefits and risks of cerebrolysin.

Quality of evidence: **Very Low** ⊕

Strength of recommendation: No recommendation

Expert Consensus Statement

The available evidence suggests that any cognitive benefits of actovegin and cerebrolysin are likely to be modest and there is risk of serious adverse events with treatment. Considering the balance of risks and harms, we suggest against using these agents for post stroke cognitive impairment.

Replication of the single available trial for Actovegin is needed.

Any further trials of actovegin and cerebrolysin should be adequately powered, have longer term follow-up and consider patient reported outcomes and health economic measures.

**PICO 15:** In people with post-stroke cognitive impairments, does **cognitive rehabilitation (cognitive skill training or compensation strategies)** compared to no rehabilitation, delay cognitive decline or progression to dementia, improve behavioural and psychological symptoms, improve performance in activities of daily living or decrease caregiver burden?
**Recommendation**<br><br>Due to a lack of methodologically robust trials, for most cognitive rehabilitation interventions, there is continued uncertainty on the benefits and limitations associated with these interventions for stroke survivors.<br><br>Quality of evidence: Very low ⨁<br>Strength of recommendation: no recommendation
**Expert Consensus Statement**<br><br>Although many of the available studies did not meet our inclusion criteria for this PICO, there is emerging evidence that cognitive rehabilitation, particularly compensatory strategies in the context of individually relevant functional tasks, may be beneficial for people with post-stroke cognitive impairments.<br><br>Methodologically robust trials to support definitive recommendations for clinical practice are needed.

**PICO 16.** In people with a history of stroke, do **multi-item prognostic tools** performed soon after stroke, predict future cognitive decline or dementia.
**Recommendation**<br><br>There is continued uncertainty over the advantages and disadvantages of using multi-item prognostic tools to predict cognitive outcomes following stroke.<br><br>Quality of evidence: **Very Low ⊕**<br>Strength of recommendation: No recommendation
**Expert Consensus Statement**<br><br>The quality of supporting evidence for tools to predict cognitive syndromes (incident delirium or dementia) is not sufficient to recommend their use in routine stroke care.<br><br>Further studies of prognostic tools for post-stroke cognitive syndromes should follow best practice guidance in prognosis methods and pay particular attention to ensuring appropriate sample size, handling missing data and external validation in independent populations.<br><br>Trials that assess the utility of using a prediction tool in clinical practice are also warranted.

**PICO 17:** In people with a history of stroke, **do structural features on acute brain CT imaging**, predict (at least one year from index stroke event) future cognitive decline or dementia.
**Recommendation**<br><br>In patients with acute stroke there is continued uncertainty regarding the value of acute CT-brain imaging findings for predicting cognitive outcomes more than one year after stroke.<br><br>Quality of evidence: **Very low** ⊕<br>Strength of recommendation: no recommendation
**Expert Consensus Statement**<br><br>As CT is the most widely available and commonly used imaging modality in acute stroke, a better understanding of the prognostic value of the imaging findings for future cognitive prognosis would be useful.<br><br>Further studies of the predictive value of CT-based imaging variables should use standardized measurements and validated tools.<br><br>Consideration needs to be given to the population included, with preferably unselected samples and low rates of attrition from cognitive follow-up.<br><br>Results of these studies need appropriate adjustments to distinguish the added prognostic value of CT imaging features over standard clinical factors such as age, sex and stroke severity.

**PICO 18** In people with a history of stroke, **do structural features on acute brain MR imaging**, predict (at least one year from index stroke event) future cognitive decline or dementia.
**Recommendation**<br><br>We suggest that in patients with acute stroke, the presence of substantial white matter hyperintensities of presumed vascular origin on acute MRI brain may help predict cognitive outcomes more than one year after stroke.<br><br>Quality of evidence: **Moderate** ⨁<br>Strength of recommendation: **Weak for intervention** ↑?<br><br>In patients with acute stroke there is continued uncertainty regarding the value of acute MRI brain imaging findings, other than white matter hyperintensities, to predict cognitive outcomes more than one year after stroke.<br><br>Quality of evidence: **Very Low** ⨁<br>Strength of recommendation: no recommendation
**Expert Consensus Statement**<br>At present, the evidence for prognostic utility in predicting future cognitive decline after stroke is most convincing for white matter lesions.<br><br>However, the added predictive value of imaging findings over and above routinely acquired clinical factors remains uncertain.<br><br>Further studies of the predictive value of MRI-based imaging variables should use standardized measurements and validated tools.<br><br>Consideration needs to be given to the population included, with preferably unselected samples and low rates of attrition from cognitive follow-up.<br><br>Results of these studies need appropriate adjustments to distinguish the added prognostic value of MRI imaging features over standard clinical factors such as age, sex and stroke severity.
